# Genome-wide analysis of the basic leucine zipper (bZIP) transcription factor gene family in six legume genomes

**DOI:** 10.1186/s12864-015-2258-x

**Published:** 2015-12-10

**Authors:** Zhihui Wang, Ke Cheng, Liyun Wan, Liying Yan, Huifang Jiang, Shengyi Liu, Yong Lei, Boshou Liao

**Affiliations:** Key Laboratory of Biology and Genetic Improvement of Oil Crops, Ministry of Agriculture, Oil Crops Research Institute of the Chinese Academy of Agricultural Sciences, Wuhan, China

**Keywords:** bZIP gene family, Legume genomes, Evolution, Expression analysis

## Abstract

**Background:**

Plant bZIP proteins characteristically harbor a highly conserved bZIP domain with two structural features: a DNA-binding basic region and a leucine (Leu) zipper dimerization region. They have been shown to be diverse transcriptional regulators, playing crucial roles in plant development, physiological processes, and biotic/abiotic stress responses. Despite the availability of six completely sequenced legume genomes, a comprehensive investigation of bZIP family members in legumes has yet to be presented.

**Results:**

In this study, we identified 428 bZIP genes encoding 585 distinct proteins in six legumes, *Glycine max*, *Medicago truncatula*, *Phaseolus vulgaris*, *Cicer arietinum*, *Cajanus cajan*, and *Lotus japonicus*. The legume bZIP genes were categorized into 11 groups according to their phylogenetic relationships with genes from Arabidopsis. Four kinds of intron patterns (a–d) within the basic and hinge regions were defined and additional conserved motifs were identified, both presenting high group specificity and supporting the group classification. We predicted the DNA-binding patterns and the dimerization properties, based on the characteristic features in the basic and hinge regions and the Leu zipper, respectively, which indicated that some highly conserved amino acid residues existed across each major group. The chromosome distribution and analysis for WGD-derived duplicated blocks revealed that the legume bZIP genes have expanded mainly by segmental duplication rather than tandem duplication. Expression data further revealed that the legume bZIP genes were expressed constitutively or in an organ-specific, development-dependent manner playing roles in multiple seed developmental stages and tissues. We also detected several key legume bZIP genes involved in drought- and salt-responses by comparing fold changes of expression values in drought-stressed or salt-stressed roots and leaves.

**Conclusions:**

In summary, this genome-wide identification, characterization and expression analysis of legume bZIP genes provides valuable information for understanding the molecular functions and evolution of the legume bZIP transcription factor family, and highlights potential legume bZIP genes involved in regulating tissue development and abiotic stress responses.

**Electronic supplementary material:**

The online version of this article (doi:10.1186/s12864-015-2258-x) contains supplementary material, which is available to authorized users.

## Background

A defining feature of transcription factors is that they contain one or more sequence-specific DNA-binding domains that bind to the promoter and/or enhancer regions of target genes to regulate gene expression [1, 2]. The basic leucine (Leu) zipper (bZIP) transcription factor family, one of the most diverse transcription factors families, is characterized by a highly conserved bZIP domain which is 60–80 amino acids in length and composed of two parts: a basic region and a Leu zipper [3]. The basic and Leu zipper regions are structurally and functionally distinct. The basic region comprises approximately 16 amino acid residues with the invariant motif N-x7-R/K-x9 and is responsible for nuclear localization and DNA binding, whereas the Leu zipper is composed of heptad repeats of Leu or other bulky hydrophobic amino acids positioned exactly nine amino acids towards the C-terminus and mediates homo- and/or heterodimerization of bZIP proteins [1, 2]. bZIP transcription factor encoding genes have been identified extensively in plants including Arabidopsis [4], rice [5], sorghum [6], maize [7], grapevine [8], cucumber [9], castor bean [10] and barley [11] with the availability of their whole genome sequences.

Like other transcription factors, members of the bZIP transcription factor family are expressed constitutively or in an organ-specific [12, 13], stimulus-responsive [14], development-dependent [15] or cell cycle-specific [16] manner in plants. It has been reported that bZIP transcription factors are involved in various biological processes including organ and tissue differentiation, embryogenesis, seed maturation and storage protein gene regulation, floral transition and initiation, and vascular development [17–21]. Moreover, bZIP transcription factors are also regarded as important regulators in signaling and responses to abiotic/biotic stimuli, including abscisic acid (ABA) signaling, hypoxia, drought, high salinity, cold stress, hormone and sugar signaling, light responses, osmotic stresses and pathogen defense [7, 12, 22–26]. In soybean, three bZIP genes were found to function as negative regulators of ABA signaling and confer salt and freezing tolerance in transgenic Arabidopsis [27].

In recent years, legume genome sequencing projects have been initiated and completed in *Lotus japonicus* (*Lj*) [28], soybean (*Glycine max*, *Gm*) [12], *Medicago truncatula* (*Mt*) [29], pigeonpea (*Cajanus cajan*, *Cc*) [30], chickpea (*Cicer arietinum*, *Ca*) [31] and common bean (*Phaseolus vulgaris*, *Pv*) [32]. These six legumes belong to the large Papilionoideae subfamily and fall within two sub-clades of the Papilionoid legumes: the Phaseoloids (warm season legumes) and Hologalegina (cool season legumes). The Phaseoloids are mostly tropical and include the crops soybean (*Gm*), pigeonpea (*Cc*) and common bean (*Pv*), while the Hologalegina are mostly temperate and include *Medicago* (*Mt*), chickpea (*Ca*) and *Lotus* (*Lj*). Among the widespread genome duplications throughout the history of flowering plants [14, 33], two recent whole genome duplication (WGD) events have affected the evolution of legume genomes. The older polyploidy event, shared by all legumes, is estimated to have occurred 56–65 million years ago (Mya) [34, 35], while the more recent genome duplication event occurred up to 13 Mya only in the lineage leading to *Glycine* [12]. Genome duplication and subsequent fractionation have played key roles in shaping present-day legume genomes and also the sizes of gene families [36].

With the availability of these legume genome sequences, the members of the bZIP transcription factor family were systematically investigated and analyzed in this study. We identified all legume bZIP genes and analyzed their bZIP domain sequences, gene structure and additional MEME motifs, which was in agreement with and supported the phylogenetic classification. Then, we predicted the DNA-binding-site specificity and dimerization properties of the legume bZIP proteins. We also investigated the impact of the two legume-lineage WGDs and tandem duplication on the expansion of the legume bZIP gene family. By analyzing their expression profiles, legume bZIP genes constitutively or specifically expressed in different tissues and seed developmental stages were identified, as well as candidate legume bZIPs responsive to drought and salt stresses.

## Methods

### Identification of bZIP transcription factors in six legume genomes

All genomic sequences and annotated proteins of the six legumes were downloaded from ftp://ftp.jgi-psf.org/pub/compgen/phytozome/v9.0/Gmax/ (*G. max*, v9), http://jcvi.org/medicago/display.php?pageName=General&section=Download (*M. truncatula*, v4.0), http://genome.jgi.doe.gov/pages/dynamicOrganismDownload.jsf? organism = PhytozomeV10 (*P. vulgaris*, v10), http://cicar.comparative-legumes.org/ (*C. arietinum*, chickpea, v1.0), http://www.icrisat.org/gt-bt/iipg/Genome_Manuscript.html (*C. cajan*, v1.0) and ftp://ftp.kazusa.or.jp/pub/lotus/lotus_r2.5/ (*L. japonicus*, v2.5).

To identify all the possible bZIP proteins in the six legume genomes, both local BLAST and hidden Markov model (HMM, http://hmmer.org/) searches were performed. For BLASTP, the known bZIP proteins from Arabidposis [4], rice [5] and maize [7] were used as queries and the e-value was set to 1e-5. For the HMM search, the profile of bZIP domain was used and the e-value threshold was set at 1. The sequences were further analyzed to confirm the presence and integrity of the bZIP domain through the ExPASy Proteomics Server (http://prosite.expasy.org/) [37] and Interpro (http://www.ebi.ac.uk/interpro/) [38]. All bZIP domain sequences were aligned using MAFFT 7 [39] to manually check and remove sequences with incomplete domains. The nomenclature was based on the exact positions of the bZIP genes on the chromosomes/scaffolds from top to bottom. Distinct transcripts encoded by the same gene locus shared the same gene number with an additional decimal part, such as point 1 or 2 (Additional file 1).

### Sequence alignment and phylogenetic analysis

The bZIP amino acid sequences from *A. thaliana* and the six legume genomes were aligned using ClustalX 2.0 [40] with gap opening and gap extension penalties of 10 and 0.1, respectively. The phylogenetic tree was reconstructed by the maximum likelihood (ML) method using the PhyML 3.0 software [41]. JTT + G was selected as the best model for constructing the phylogenetic tree by the Akaike information criterion implemented in ProtTest 3.0 [42]. Bootstrap values from 100 replicates were indicated at each node. MEGA5 [43] was used to show the tree.

### Structure of bZIP genes

The positional information of both the gene sequence and the corresponding coding sequence were loaded into the gene structure display server v2.0 (http://gsds.cbi.pku.edu.cn/) [44] to obtain information on the intron/exon structure. The coordinates of the bZIP domain in each protein were recalculated into the coordinates in gene sequence and featured in gene structure. We used Genewise [45] to analyze the intron distribution pattern and intron splicing phase within the basic and hinge regions of the bZIP domains in the six legumes.

### Detection of additional conserved motifs

To identify additional conserved motifs outside the bZIP domain of legume bZIP transcription factors, we used the Multiple Em (Expectation Maximization) for the Motif Elicitation tool (MEME version 4.9.1, http://meme.nbcr.net/meme/) [46]. The limits for maximum width, minimum width and maximum number of motifs were specified as 50, 10 and 100, respectively. Fifty motifs were finally confirmed because of their low e-values (<1e-200). The motifs were numbered according to the order displayed in MEME and were considered as group-specific signatures for their presence of high frequency in the given groups.

### Detection of duplicated genes and estimation of synonymous (Ks) and nonsynonymous (Ka) substitutions per site and their ratio

The duplicated gene pairs derived from segmental duplication were identified in the legume genomes based on the method from the Plant Genome Duplication Database [23]. An all-against-all BLASTP comparison (e-value: 1e-5) provided the gene pairs for syntenic clustering determined by MCScan (using default settings: MATCH_SCORE: 50, MATCH_SIZE: 5, GAP_SCORE:–3, E_VALUE: 1E–05) (http://chibba.agtec.uga.edu/duplication/mcscan). Tandem duplication arrays were identified using BLASTP with a threshold of e < 10^−20^, and one unrelated gene among cluster members was tolerated, as described in *A. thaliana* [26]. Pairs from segmental or tandem duplications were used to estimate Ka, Ks and their ratio. Amino acid sequences from segmentally or tandemly duplicated pairs were first aligned and then guided and transferred into a cDNA sequences alignment using in-house Perl scripts. Then, the software KaKs_Calculator was used to compute Ka and Ks values for each pair following the YN model [24].

### Expression analysis of legume bZIP genes

For different tissues/organ and seed developmental stages, the normalized counts for bZIP genes from RNA-seq were obtained from the Soyseq (http://www.soybase.org/) [47] and PvGEA (http://plantgrn.noble.org/PvGEA/) databases [48]. Microarray expression values for *Medicago* and *Lotus* were downloaded from http://mtgea.noble.org/v3/ [49, 50] and http://ljgea.noble.org/ v2/ [51]. To identify candidate bZIP genes responsive to drought and/or salt stresses, microarray gene expression data in *Medicago* were downloaded from Zhang et al. [52] and Li et al. [53], respectively. The gene expression changes in drought-stressed roots and shoots (treatment at days 3, 4, 7, 10 and 14) were obtained by comparing with levels in the watered control (drought day 0). Similarly, the fold changes in gene expression were calculated when comparing the salt-stressed roots (treatment with 180 mM NaCl at 6, 24, and 48 h) and control (treatment at 0 h). The corresponding relationships between microarray probes and legume bZIP genes were built using BLAST (best hit under 1e-10). The expression values or normalized counts were log10-transformed and the gplots package was used to make a heatmap in R.

## Results and discussion

### Identification and nomenclature of the legume bZIP transcription factor family

Through sequence similarity and domain searches, 138 *Gm*, 65 *Mt*, 72 *Pv*, 59 *Ca*, 61 *Cc* and 33 *Lj* bZIP genes, encoding 241, 99, 92, 59, 61 and 33 distinct proteins, respectively, were identified in the six legume genomes. Based on their exact positions on chromosomes/scaffolds (from top to bottom), we gave a unique name to each bZIP protein. The related information on bZIP transcription factors are listed in the Additional file 1.

Domain analysis showed that all 585 of the bZIP transcription factors except eight had a typical bZIP domain with an invariant N-x7-R/K motif in the basic region and a heptad repeat of Leu or other bulky hydrophobic amino acids positioned exactly nine amino acids upstream of R/K toward the C-terminus (Additional file 2). Of the remaining eight, in Glyma12g04933.1 (*GmbZIP80*) and Phvul.011G047100.1 (*PvbZIP67*), the conserved Asn (N) in the basic region was replaced by Lys (K). In Glyma03g35101.1 (*GmbZIP21*), Glyma19g37801.1 (*GmbZIP128*), Medtr7g104190.1 (*MtbZIP52*) and Ca_00780 (*CabZIP15*), the conserved Arg/Lys (R/K) in the basic region was substituted by an Ile (I), whereas in Glyma11g28880.1 (*GmbZIP75*), the conserved Arg/Lys (R/K) in the basic region was substituted by a Trp (W). In C. Cajan19144 (*CcbZIP24*), the heptad repeat of Leu was positioned at 23 amino acids toward the C terminus instead of the usual nine. All of these unusual changes in the bZIP domain have been observed in rice bZIP sequences previously [5, 54].

### Phylogenetic analysis and classification of legume bZIP genes

**Fig. 1:**
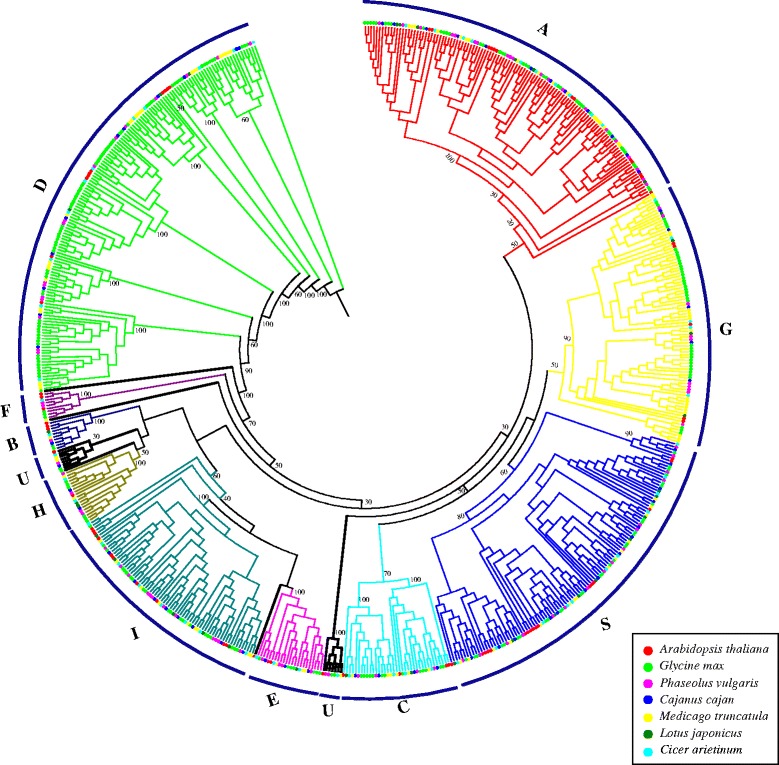
Phylogenetic tree of legume and Arabidopsis bZIP genes. bZIP protein sequences were aligned by Clustal X and the phylogenetic tree was constructed using PhyML by the maximum likelihood method. Bootstrap values are based on 100 replicates. Genes on branch ends from different species are denoted by different colored circles. The legume bZIP proteins were grouped into 11 distinct clades (A–I, S, U), which are indicated by colored branches

To investigate the phylogenetic relationships of the bZIP transcription factors, 585 protein sequences from the six legumes and 71 protein sequences from Arabidopsis (three genes were no longer supported by their updated annotations) [4] were analyzed (Fig. 1 and Additional file 3). In accordance with the bZIP classification in Arabidopsis [4], the phylogenetic tree was subdivided into 10 clades with well-supported bootstrap values. All groups contained legume bZIP proteins clustered together with *AtbZIP* proteins in the same clade, except group F, which included members from only three genomes: *G. max*, *P. vulgaris* and *C. arietinum*. In addition, 15 legume bZIP proteins and 3 *AtbZIP* genes (*AtbZIP60*, *AtbZIP62* and *AtbZIP72*) formed two small unique and several other individual clades (bold black branch in Fig. 1), which were classified into the unclassified group (group U) based on their possible independent evolutionary trajectories from other clades. Group classification was supported by the group-specific sequence characteristics identified in the following analyses of gene structures, intron phases, additional conserved motifs outside the bZIP domain and DNA-binding site specificity in each group. It is evident that the group-specific sequence characteristics of the bZIP members formed before the divergence of Arabidopsis and legumes since conserved sequence characteristics were present in the same group containing both Arabidopsis and legumes bZIP genes. Nevertheless, it seems that intra-species duplication and parallel evolution of the bZIP family in each legume has occurred afterward and contributed to the member variation in each group.

### Gene structure of legume bZIP genes

The intron-exon organization can reflect the evolutionary trajectory of gene families [5, 7, 55, 56]. We examined the gene structures of all 585 legume bZIPs and found that the structural patterns were similar among members within each group but distinct between different groups (Additional file 4). The number of introns in each group was uneven but relatively concentrated (Additional file 5). We detected 29 *GmbZIP* genes (12.03 %), 15 *MtbZIP* genes (15.15 %), 16 *PvbZIP* genes (17.39 %), 12 *CabZIP* genes (20.34 %), 13 *CcbZIP* genes (21.31 %) and 9 *LjbZIP* genes (27.27 %) with no introns. Most of these intronless genes were clustered into groups S and F (Additional files 3 and 4). Among the intron-containing *bZIP* genes, the number of introns within the open reading frame (ORF) varied from 1 to 11 in *GmbZIP*, 15 in *MtbZIP*, 11 in *PvbZIP*, 11 in *CabZIP*, 11 in *CcbZIP*, and 8 in *LjbZIP*, close to the highest number of introns within the ORF reported in Arabidopsis (12) [4], rice (12) [5], sorghum (14) [6], maize (14) [7], castor bean (11) [10], barely (11) [11] and cucumber (12) [9]. The bZIP genes with the most introns were commonly found in groups D and G (Additional file 4).

**Fig. 2:**
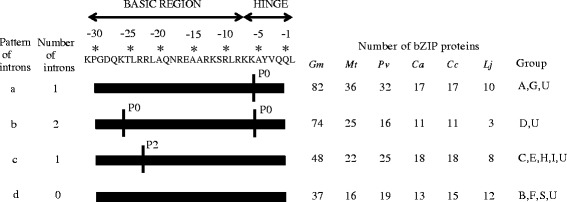
Intron patterns within the basic and hinge regions of the legume bZIP domain. The primary structure of the bZIP domain is shown at the top of the picture. P0 indicates the intron splicing site is between codons, and P2 means the intron splicing site is located between the second and third nucleotides of one codon. Based on the intron incidence and positions, as well as the splicing phase, the legume bZIP genes were divided into four patterns (a–d). Details of the intron positions within the bZIP domain in the legume bZIP proteins are shown in Additional file 2

The intron positions within the ORF were diverse. The phases of the splicing sites within the ORFs also differed, but the positions and phases of introns in the basic and hinge regions of the bZIP domain were highly conserved. The legume bZIP genes showed four intron patterns (a–d) based on the intron positions, presence or number and splicing phases within the basic and hinge regions (Fig. 2 and Additional file 2). Pattern a, having one intron in phase 0 (P0 indicates the intron splicing site is between codons) within the hinge region at the −5 position, was seen in all members of groups A and G in the legumes. Pattern b, having two introns each in phase 0: one in the basic region at the −25 position and the other in the hinge region at the −5 position, was seen in all members of group D in the legumes. Pattern c, having a single intron in phase 2 (P2 means the intron splicing site is located between the second and third nucleotides of one codon) at the −20 position in the basic region, was seen in all members of groups C, E and H in the legumes. Pattern c also was seen in all members of group I in *G. max, M. truncatula*, *C. arietinum* and *C. cajan*, and most members (3/5) of group I in *L. japonicus*. Pattern d was no introns in the basic and hinge regions and was seen in groups B, S and F, which contained most of the intronless genes. Among 106 genes showing pattern d, 85 were intronless, while the remaining 22 had introns outside the basic and hinge regions. In summary, the overall pattern of intron position acts as an index for the group classification and phylogenetic relationships in the legume bZIP gene family. The splicing phase has remained well conserved during the course of evolution in legume bZIP genes.

### Identification of additional structural features in the legume bZIP genes

**Fig. 3:**
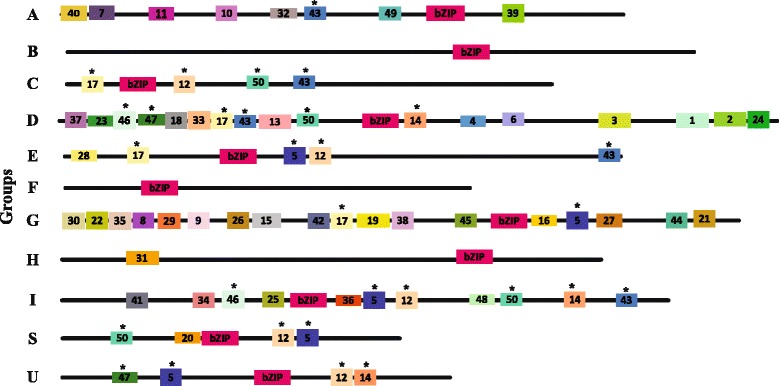
Distribution of additional conserved motifs identified by MEME. Motif compositions based on the position of the bZIP domain and additional conserved motifs outside the bZIP domain in representative legume bZIP proteins for each group are shown. The bZIP domains are shown in pink and different motifs are highlighted in different colored boxes with numbers 1 to 50. The motifs shared by different groups are marked with black stars. Details for the predicted conserved motifs are given in Additional file 6

All legume bZIP protein sequences were loaded into the MEME analysis tool and a total of 50 additional conserved motifs outside the bZIP domain were identified. The multi-level consensus sequences and the amino acid lengths of these conserved motifs are given in Additional file 6. The legume bZIP proteins within the same group had similar motif compositions, suggesting conserved evolution and supporting the group classification (Fig. 3). Additionally, some motifs were shared by different groups, such as motifs 46 and 47 in two groups, motif 14 in three groups, motifs 17 and 50 in four groups, and motifs 5,12 and 43 in five groups. Nevertheless, most of the conserved motifs appeared specific to each group (Additional file 6) and therefore the group-specific motifs could help determine the specific functions of members in each group. Notably, we did not detect any additional conserved motifs outside the bZIP domain in groups B or F.

A few of these motifs in legume bZIPs have been studied for their possible biological functions. A part of motifs 7, 10 and 11 represent potential casein kinase II (CK II) phosphorylation sites (S/TxxD/E), indicated by the motif patterns [TS][AV]E[AE], TLGE(TLED) and TVDE. Motif 11 also contained a phosphorylation site for the Ca^2+^-dependent protein kinase (R/KxxS/T), presented as RQ[GA]S. Motif 31 in group H was group-specific, and contained potential CK II phosphorylation sites (S/TxxD/E), indicated by [SP]CYE. Such motifs were identified in group A exclusively. In addition, all members in group D shared motif 1, which was a DOG1 domain, and motif 4, the function of which was unclear (Additional file 6). Motifs 9, 22 and 29 were observed in group G exclusively and were characterized by a part of the proline-rich domain, which has been shown to have transcriptional activation potential. Interestingly, there were some common motifs among the six legumes, maize and rice [5, 7]. For example, motif 11 in group A was the same as motif 18 in group A in maize. Motifs 1, 2 and 3 in group D are in common with motifs 1, 2 and 5 in group D of maize and motifs 18, 20 and 19 of rice, respectively.

### Prediction of DNA-binding-site specificity in legume bZIP proteins

Experiments of mutant proteins demonstrated that the bZIP TFs binding specificity is independently determined by the core basic region and the hinge region, and the two regions have an additive effect on DNA-binding specificity [57, 58]. To predict the DNA-binding-site specificity of the legume bZIP proteins, the amino acid sequences of the basic and hinge regions of 585 legume bZIP proteins were aligned, revealing some highly conserved amino acid residues within each group (Additional file 7). We can predict the DNA-binding specificity in a group manner, as described in Additional file 8. Furthermore, the amino acids were numbered as previously reported [5, 7, 59], and the first Leu in the Leu heptad repeats was numbered +1. For the two invariant sites asparagine (Asn/N) and arginine (Arg/R), numbered −18 and −10, respectively, new DNA-binding specificities will occur if other amino acids functionally replace these two invariant sites (N and R) [57]. Nevertheless, such replacements were infrequent and occurred only in groups G and U in the legume bZIP proteins. At position −18, the conserved asparagine (N) was replaced with lysine (K) in *GmbZIP80* and *PvbZIP67* in group G (Additional file 7). The same replacement (from N to K) was also observed in four bZIPs in maize [7], two bZIPs in barley [11], and two bZIPs in castor bean [10]. At position −10, nine members in group U had a hydrophobic isoleucine (Ile/I) residue instead of arginine (R) or lysine (K). An identical substitution pattern (from R/K to I) was observed in two bZIPs in maize [7], two in barley [11], one bZIP in grapevine [8], and one bZIP in castor bean [10]. It was demonstrated that an arginine to isoleucine mutation in the basic domain of the yeast bZIP factor GCN4 completely inhibited its affinity for the AP1 site [57]. Additionally, it was reported that OsZIP-2a belonging to group U in rice, because of this replacement, does not recognize G-boxes [54]. This evidence suggests that unusual substitutions in the DNA-binding domain affect the DNA-binding specificity. These predictions were made to facilitate further studies on the DNA-binding patterns of the legume bZIP transcription factors.

### Prediction of dimerization properties in legume bZIP proteins

Studies have demonstrated that the Leu zipper region of the bZIP domain, arranged in the form of heptad repeats, mediates homo- and/or heterodimerization between the parallel coiled-coil structures [60–62]. Within each heptad, the amino acid positions are recognized in order as *g*, *a*, *b*, *c*, *d*, *e*, and *f* [63, 64] (Additional file 9). Leu zipper oligomerization, dimerization stability and specificity are determined mainly by the four amino acids present at the *a*, *d*, *e* and *g* positions because of their special positions near the Leu zipper interface. The *a* and *d* residues are typically hydrophobic on the surface of the helix and create a hydrophobic core that promotes the interaction between two monomers and is essential for dimer stability [65]. The *a* position contains asparagine (Asn/N), which can form a polar pocket in the hydrophobic interface that limits oligomerization in interhelical interactions [66] and produce more stable N–N interactions at *a*↔*a*ʹ (the corresponding position in the opposite helix) than other amino acids [64]. The conserved Leu at the *d* position, one of the most stabilizing aliphatic amino acids [67], is important to maintain dimer stability. However, the *e* and *g* positions that flank the dimerization interface frequently contain charged amino acids including the acidic amino acids glutamic acid (E) and aspartic acid (D), and the basic amino acids arginine (R) and lysine (K), which are thought to form salt bridges between helices in electrostatic interactions [68].

**Fig. 4:**
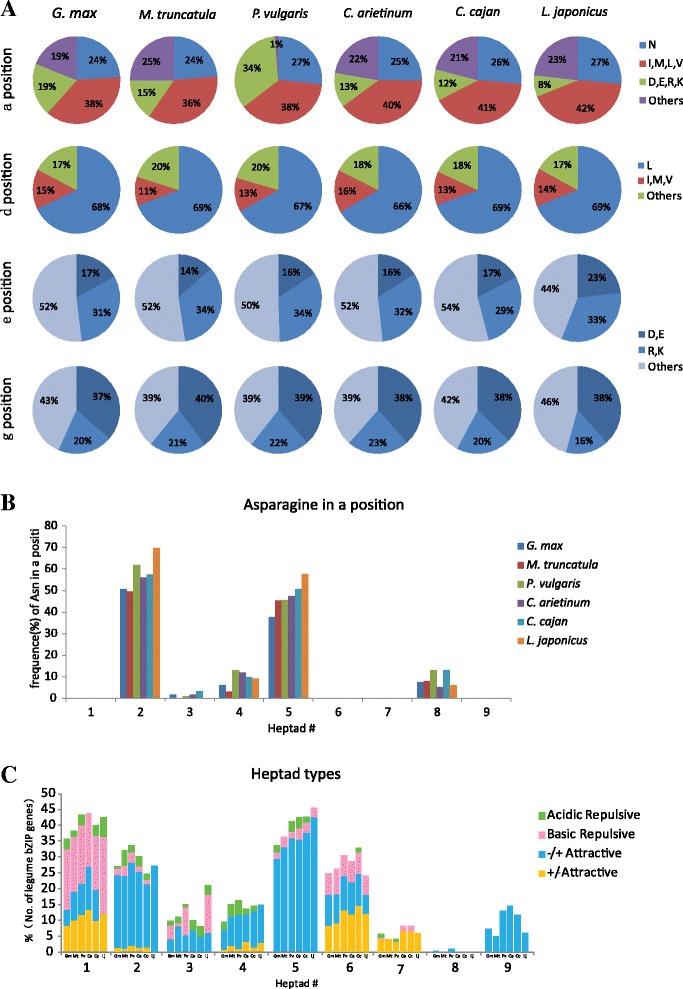
Prediction of dimerization properties of the legume bZIP proteins. **a** Pie charts presenting the frequency of amino acids in all the *a*, *d*, *e* and *g* positions of the Leu zipper in legume bZIP domains. **b** Histogram of the frequency of Asn (N) in the *a* positions of the Leu zippers among legume bZIP proteins. **c** Histogram of the frequency of attractive or repulsive *g*↔*eʹ* pairs per heptad for legume bZIP proteins

To predict the dimerization properties of the legume bZIP transcription factors, a detailed analysis was carried out to characterize the amino acids present at the *a*, *d*, *e* and *g* positions (Additional file 9). Figure 4a shows the composition of different kinds of amino acids found in the *a*, *d*, *e* and *g* positions in the six legume bZIPs, respectively. At the *a* position, about 25 % of the residues were asparagine (Asn/N), suggesting that there will be a greater number of homodimerizing Leu zippers through stable N–N interhelical interaction at the *a*↔*a*ʹ position among legume bZIP proteins. The frequency of asparagine (Asn/N) residues in the *a* position was highest in the second heptad followed by the fifth heptad, accounting for 57.51 % and 47.45 %, respectively (Fig. 4b), similar to earlier observations for *AtbZIP* proteins [64]. At the *d* position, the frequency of Leu, responsible for dimer stability, was about 68 % in the legume bZIPs (Fig. 4a), which is a little less than in *OsbZIPs* (71 %) [5] and *ZmbZIPs* (70 %) [7], but significantly greater than in *AtbZIPs* (56 %) [64]. At the *e* and *g* positions (Fig. 4a), the frequencies of charged amino acids (including acidic amino acids D and E, and basic amino acids R and K) were 49 and 59 %, respectively.

In electrostatic interactions, the amino acids at position *g* and oppositely charged amino acids at position *e*ʹ (the prime means a residue on the opposite helix of the leucine zipper) can form interhelical salt bridges that determine the dimerization specificity and stability [68]. The dimerization specificity of human, Drosophila, Arabidopsis, rice, maize, barley and castor bean bZIP TFs has been predicted on the basis of attractive or repulsive interhelical *g↔eʹ* electrostatic interactions [5, 7, 10, 11, 64, 69, 70]. To analyze the contribution of charged residues to the dimerization properties of the legume bZIP proteins, we calculated the frequency of attractive and repulsive *g*↔*e*ʹ pairs in each heptad of the bZIP Leu zippers; the corresponding histograms are shown in Fig. 4c. If both the *g* and corresponding *e* position amino acids are charged, they are referred to as complete *g↔eʹ* pairs. This analysis was carried out on the basis of the frequency of attractive and repulsive *g*↔*e*ʹ pairs, which were classified into four groups, attractive basic-acidic pairs (+/−attractive), attractive acidic-basic pairs (−/+ attractive), repulsive basic pairs (basic repulsive) and repulsive acidic pairs (acidic repulsive) in the heptads. Attractive *g*↔*e*ʹ pairs were predominant in the second, fifth and sixth heptads, thereby suggesting the chances of heterodimerization (Fig. 4c). In contrast, few complete *g*↔*e*ʹ pairs were observed in the eighth heptads except two attractive acidic-basic (−/+ attractive) pairs from *GmbZIP102* and *PvbZIP4* (Additional file 9). Moreover, only attractive acidic-basic (−/+ attractive) pairs were present in ninth heptads (Fig. 4c), which was similar to observations in *HvbZIPs* [11], *OsbZIPs* [5] and *ZmbZIPs* [7].

We divided the 585 legume bZIP proteins into 44 subfamilies (BZ1–BZ44) on the basis of the defining properties of dimerization specificity [64, 70]. These subfamilies were divided into three general groups: (i) those that strongly favor homodimerization within eight subfamilies (BZ1–BZ8), (ii) those with both homo- and heterodimerization properties (BZ9–BZ39) and (iii) those that strongly favor heterodimerization within five subfamilies (BZ40–BZ44). The results indicated the complexity and diversity of dimerization patterns in legume bZIP proteins, with the potential to homodimerize with themselves or with members in the same subfamily as well as heterodimerize with other subfamily members, which has been shown in maize [7], rice [5], and Arabidopsis [64]. Based on the criteria used to define the boundaries and natural C-terminus, we observed that the length of the Leu zipper in the bZIP transcription factor family was variable, ranging from two to nine heptads. Among the bZIP proteins, 2.39 % had only two short zippers and belonged to BZ43 and group G, about 28 % had only three short zippers (mainly in BZ40–BZ42 and groups D and E), and about 9 % had no α-helix breakers for 10 or more heptads, were mainly distributed in BZ37–BZ39 and included members of group I.

### The impact of whole genome duplication and tandem duplication on the expansion of the legume bZIP gene family

**Fig. 5:**
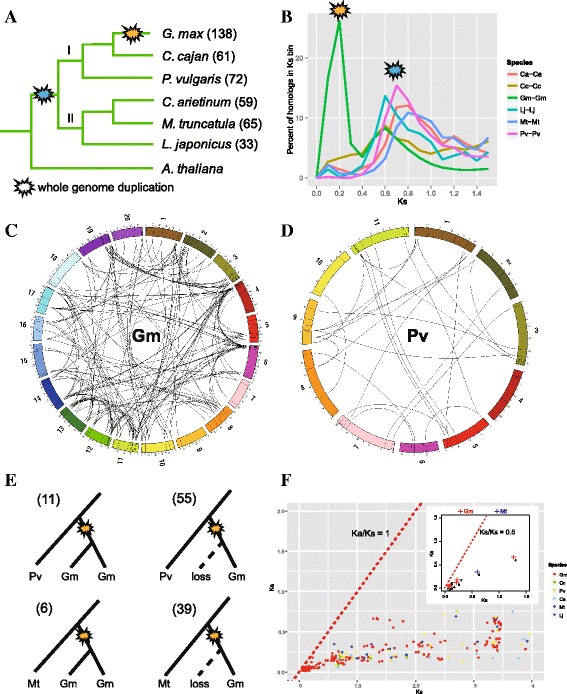
Whole genome duplication (WGD)-derived legume bZIPs genes. **a** The phylogenetic relationships of the six legumes based on the concentrated orthologous sequences of *rbc* and *matk* genes. Two WGD events, the *Glycine*-lineage-specific and early-legume WGD events, are indicated on the corresponding clades. **b** The Ks distribution of paralogs derived from WGD-derived duplicated genomic blocks in each legume. Two obvious peaks correspond to the old and recent legume-lineage WGD events. **c**, **d** The black lines in the ideogram show the chromosomal positions of all identified bZIP genes, and the duplicated bZIP pairs are linked by lines in soybean (**c**) and common bean (**d**). **e** Patterns of gene retention or loss indicated by two different informative tree topologies using common bean and *Medicago* as outgroups, respectively. The numbers for each pattern are shown in parentheses. **f** The Ks (x-axis) and Ka (y-axis) distribution for each duplicated legume bZIP gene pair. The red dashed line means the Ka/Ks ratio is equal to 1. Particularly, eight *Gm* duplicated pairs (red cross) and one *Mt* pair (blue cross), whose Ka/Ks values were greater than 0.5, are indicated in the inner figure (under the dashed line of Ka/Ks = 0.5). The red dashed line in inner figure means the Ka/Ks ratio is equal to 0.5. The numbers (1–9) correspond to duplicated pairs: 1: *GmbZIP23-GmbZIP131*; 2: *GmbZIP49-GmbZIP109*; 3: *GmbZIP55-GmbZIP107*; 4: *GmbZIP8-GmbZIP113*; 5: *GmbZIP4-GmbZIP69*; 6: *GmbZIP26-GmbZIP42;* 7: *GmbZIP87-GmbZIP101;* 8: *GmbZIP65-GmbZIP113;* 9: *MtbZIP2-MtbZIP26*

To explore the expansion mechanism in detail, we analyzed the contribution of WGD –derived segmental duplication and tandem duplication to the expansion of the legume bZIP gene family. First, a phylogenetic tree was constructed based on the concentrated orthologous sequences of *rbc* and *matk* genes from the six legume genomes, which supported the classification of two sub-clades of Papilionoideae legumes: Phaseoloids (clade I, including *Gm*, *Cc* and *Pv*) and Hologalegina (clade II, including *Mt*, *Ca* and *Lj*) (Fig. 5a). We carried out a genome-wide identification of collinear duplicated blocks derived from segmental duplication in each species, and then examined the pairwise synonymous distances (Ks values) of paralogs within duplicated collinear blocks. By plotting their distribution, two distinct peaks were found (Fig. 5b: Ks bin = 0.1): one was specific to *Gm-Gm* paralogues, while the other was observed in all six legumes.

Second, the chromosomal distribution of the legume bZIP genes was plotted and the bZIP gene pairs on duplicated chromosomal collinear segments were connected by lines (Fig. 5c, d, Additional files 10 and 11). Among these genes, some were segmentally duplicated once and some were duplicated twice or thrice. The duplication occurred within a chromosome or between chromosomes. We detected 119 *Gm*, 24 *Mt*, 38 *Pv*, 30 *Ca*, 8 *Cc* and 2 *Lj* bZIP genes involved in segmental duplication, accounting for around 86.2 % (119/138, *Gm*), 36.9 % (24/65, *Mt*), 52.8 % (38/72, *Pv*), 50.8 % (30/59, *Ca*), 13.1 % (8/61, *Cc*), and 6.1 % (2/33, *Lj*) of the bZIP genes in each species. The higher ratio in soybean reflects the preferential gene retention after multiple rounds of WGD, while the different ratios among the other five legumes may be mainly attributable to the genome assembly quality (for example, relatively incomplete in *Cc* and *Lj*) or species-specific evolution in each genome.

Moreover, we roughly identified different WGD event origins for duplicated bZIP gene pairs according to their pairwise synonymous distances using criteria from soybean [12]: Ks values of 0.06–0.39 correspond to the 13-Mya Glycine-lineage-specific WGD, and Ks values of 0.40–0.80 in soybean and 0–1.00 in the other five legumes correspond to the 59-Mya early-legume WGD, while larger Ks values correspond to more ancient WGD events like the ‘gamma’ event [14]. By ordering the Ks values, 55 *Gm* bZIP gene pairs were associated with the 13 Mya *Glycine*-lineage-specific WGD and 28 *Gm*, 4 *Mt*, 8 *Pv*, 6 *Ca*, 2 *Cc*, and 0 *Lj* bZIP gene pairs were associated with the 59 Mya early-legume WGD; the others were associated with more ancient WGDs (Additional file 11). Because only soybean has undergone the *Glycine*-lineage-specific WGD, the other five legume genomes could be considered putative ancestors for investigating gene retention and loss after the recent genome duplication in soybean. *Mt* and *Pv* were chosen, because of their good genome assembly and annotation, to construct phylogenetic trees with *Gm* genes. We identified informative tree models of gene retention: [*Pv*, (*Gm*, *Gm*)] and [*Mt*, (*Gm*, *Gm*)], and gene loss [(*Pv*, *Gm*)] and [(*Mt*, *Gm*)], showing that 83 % (*Pv* as outgroup) and 87 % (*Mt* as outgroup) ancestral loci were retained as paralogs in soybean after the recent WGD (Fig. 5e). Compared with the average 43.4 % of genes retained as paralogs at the whole genome level [12], genes of the bZIP family were highly retained.

To better understand the evolutionary constraints acting on the legume bZIP genes, we calculated the Ka/Ks ratios for all legume duplicated bZIP gene pairs. The resulting pairwise comparison data showed that the Ka/Ks values of only eight *Gm* duplicated pairs and one *Mt* pair were larger than 0.5 (but less than 1) while all of the remaining Ka/Ks ratios were less than 0.5 (Fig. 5f and Additional file 11), suggesting that the bZIP family has mainly undergone strong purifying selection and the legume bZIP genes are slowly evolving at the protein level. We further compared the strength of selection on bZIP paralogs between the recent and old whole-genome duplications in soybean. The average Ka/Ks ratio for the recently duplicated bZIP genes (0.305) was higher than that of the early duplicated bZIP genes (0.249), and there was a significant difference between these ratios (t-test, *P* = 0.025). This indicated that the younger bZIP proteins may be under stronger evolutionary constraints than older proteins, but supported the notion that the legume bZIP gene family is essential for the regulation of cellular processes.

Third, we determined members with tandem duplication in each legume (highlighted in red in Additional file 10). We detected only 1 *Gm*, 3 *Mt*, 0 *Pv*, 0 *Ca*, 0 *Cc* and 0 *Lj* tandem gene pairs, indicating the limited contribution of tandem duplication to the expansion of the gene family. Therefore, it seems that segmental duplication rather than tandem duplication is the major mechanism driving the expansion of this gene family.

### Expression analysis of bZIP transcription factors

**Fig. 6:**
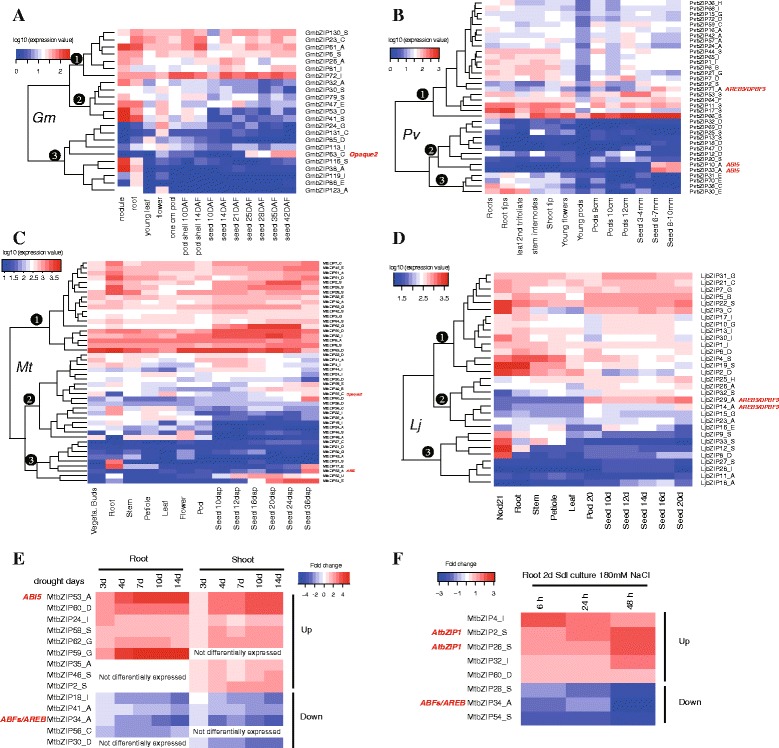
Expression profiles of legume bZIP genes. **a**–**d** Clustering of legume bZIP genes according to their expression profiles in tissues including nodules, roots, stems, flowers, and pods and seeds at different developmental stages in *Gm* (**a**), *Pv* (**b**), *Mt* (**c**) and *Lj* (**d**). The color scale represents log10 of the average signal values. **e**, **f** The up- and down- regulated bZIP genes identified in drought-stressed roots and shoots (**e**) and salt-stressed roots (**f**). The color scale represents the fold change in the gene expression value compared with the control

Expression data from different tissues (nodule, root, stem, leaf, flower, and pod) and seed developmental stages in soybean [47], *Medicago* [49, 50], common bean [48] and *Lotus* [51] were downloaded, and hierarchical clustering was performed to visualize a global transcription profile of the legume bZIP genes. As shown in Fig. 6 (a: *Gm*, b: *Pv*, c: *Mt*, d: *Lj*), the heatmaps were always divided into three recognized clusters, which was similar to the results in rice [5], maize [7] and other plants [8, 11]. The different clusters corresponded to overall differences in expression patterns including expression values and specificity across tissues. The latter was indicated by CV values (coefficient of variation), which were calculated for each gene, and helped to recognize genes expressed in specific tissues or stages (Additional file 12). Cluster I included genes with relatively high expression levels and the least expression variability (lower CV values), indicating an extensive and stable expression pattern relative to the other legume bZIP genes. Cluster II included genes with variable and moderate expression. Cluster III included genes with inconsistent (always higher CV values) but low expression in tissues. The broad expression pattern across various tissues indicated that members of the legume bZIP transcription factor family are either expressed constitutively or in an organ-specific, development-dependent manner and may be involved in organ and tissue differentiation and seed developmental processes. Among the genes that were highly expressed during seed developmental stages, some were identified as homologs/orthologs of well-studied bZIP genes from Arabidopsis [4], rice [5] and maize [7]. The *AtbZIP39/ABI5* gene, a homolog of *MtbZIP53*, *PvbZIP10* and *PvbZIP33* (extracted expression values are shown in Fig. 6), is functionally involved in ABA signaling and mediating embryogenesis in late embryo development [71]. *AtbZIP66/AREB3/DPBF3* and *AtbZIP67*/*DPBF2*, which were homologs of *PvbZIP71*, *LjbZIP29* and *LjbZIP14*, have been confirmed to play important roles in ABA-mediated seed development, germination, and embryo maturation [72]. In addition, *GmbZIP63* and *MtbZIP55* were homologous to the maize bZIP factor *Opaque2*, which controls the transcription of *a-zein*, *b-32* and *b-prolamin* genes and regulates protein accumulation, and amino acid and sugar metabolism in maize seeds [73–76].

As a kind of ubiquitous transcription factor, bZIP proteins regulate the expression of a wide spectrum of stress-related genes. We analyzed the expression values measured in drought-stressed roots and shoots corresponding to 3, 4, 7, 10, and 14 days of drought, and in salt-stressed roots upon 180 mM NaCl treatment. We identified the obvious up- and down-regulated genes (at least 2-fold, with a *P*-value < 0.05) and the log2 (treated/control) ratio values were illustrated by heatmaps (Fig. 6e, f). We detected five genes (*MtbZIP53*_A, *MtbZIP62*_G, *MtbZIP58*_S, *MtbZIP24*_I, and *MtbZIP60*_D) up-regulated both in roots and shoots under drought stress, one gene (*MtbZIP59*_G) up-regulated only in roots, and three genes (*MtbZIP35*_A, *MtbZIP46*_S, and *MtbZIP2*_S) up-regulated only in shoots. Among the genes down-regulated under drought stress, *MtbZIP18*_I, *MtbZIP41*_A and *MtbZIP34*_A were down-regulated in both roots and shoots, *MtbZIP56*_C was only down-regulated in roots, and *MtbZIP30*_D was only down-regulated in shoots. In roots under salt stress, *MtbZIP60*_D, *MtbZIP32*_I, *MtbZIP26*_S, *MtbZIP2*_S and *MtbZIP4*_I were up-regulated and *MtbZIP54*_S, *MtbZIP34*_A and *MtbZIP28*_S down-regulated. Most of the genes showing a response to drought and salt stress were concentrated in groups A and S. bZIP genes from these groups have been frequently reported to be involved in sugar signaling and abiotic stress regulation [4, 77, 78]. Notably, the CKII and Ca^2+^-dependent protein kinase phosphorylation site motifs (motifs 7, 10 and 11) confined to group A in this study have been proposed to be involved in stress and/or ABA signaling, which plays an important the role in the adaptation of plants to various abiotic environmental stress conditions like drought, high salinity, and cold stress [79]. *ABI5* (ABA insensitive 5) and ABFs (ABRE binding factors)/AREB (ABA-responsive element binding protein) have been shown to be key ABA-dependent signal transduction factors involved in abiotic stress tolerance [22, 80]. Among the stress-responsive legume bZIP genes (Fig. 6e, f), *MtbZIP53* and *MtbZIP34* were homologous to ABI5 and ABFs/AREB, respectively. In addition, two genes, *MtbZIP2* and *MtbZIP26*, were responsive to salt stress and homologous to *AtbZIP1*, which has been reported to be transcriptionally induced by salt treatment [81, 82] and leads to enhanced or reduced tolerance to salt stress when overexpressed or knocked out, respectively [83]. Overall, the expression analysis presented here improves our understanding of plant responses to stress at the molecular level and provides candidate legume bZIP genes for future research.

## Conclusions

bZIP transcription factors have been extensively characterized in eukaryotic genomes and have been shown to play crucial roles in plant development, physiological processes, and biotic/abiotic stress responses. Using the six legume genomes available, we performed an extensive study of legume bZIP genes including structure, phylogeny, sequence, and expression analyses. The group classification of legume bZIP genes based on their phylogenetic relationships was supported by subsequent analyses of gene structure, intron phases in the bZIP domain, MEME motif composition, DNA-binding specificity and dimerization patterns, which showed group-specificity. The group-specific sequence characteristics of the bZIP members should have formed before the divergence of Arabidopsis and legumes since conserved sequence characteristics were present in the same group containing both Arabidopsis and legume bZIP genes. The global expression profile supports the role of legume bZIP proteins in performing diverse developmental and physiological functions during tissue differentiation and seed development, as well as drought and salt stresses.

## Availability of supporting data

The data set supporting the results of this article is available in the Dryad Digital Repository, http://dx.doi.org/10.5061/dryad.m0qc8.

## Additional files

Additional file 1:
**The identified legume bZIP proteins and their related information.** (XLS 173 kb) Additional file 2:
**Position and pattern of introns within the basic and hinge regions of bZIP domains of the legume bZIP transcription factors.** (PDF 1398 kb) Additional file 3:
**The phylogenetic analysis of bZIP proteins based on the bZIP protein sequences from Arabidopsis (71 bZIP proteins) and six legumes (585 bZIP proteins) corresponding to Fig.** 1 **.** (PDF 1003 kb) Additional file 4:
**The map of intron-exon arrangement of legume bZIP genes.** (PDF 1488 kb) Additional file 5:
**Histogram of intron number of legume bZIP genes in each group.** (PDF 410 kb) Additional file 6:
**The detailed information of additional conserved motifs in bZIP proteins as predicted by MEME.** (XLS 176 kb) Additional file 7:
**Alignment of basic and hinge regions of 585 legume bZIP proteins.** (PDF 1687 kb) Additional file 8:
**DNA binding specificity prediction of each group.** (DOC 51 kb) Additional file 9:
**Amino acid sequence alignment of the leucine zipper region of 585 legume bZIP proteins.** (PDF 4254 kb) Additional file 10:
**Chromosomal distributions of legume bZIP genes.** (PDF 2418 kb) Additional file 11:
**bZIP genes present on duplicated chromosomal segments.** (XLS 68 kb) Additional file 12:
**Expression values of legume bZIPs in multiple tissues.** (XLS 76 kb)

## Abbreviations

WGD: Whole genome duplication
Mya: Million years ago
HMM: Hidden Markov model
ML: Maximum likelihood
ORF: Open reading frame
MEME: Multiple expectation maximization for motif elicitation
ABI5: ABA-insensitive 5
ABRE: ABA response element
ABF: ABRE binding factor
AREB: ABA-responsive element binding protein
