# Supplementary material for: Genome-wide analysis of the basic leucine zipper (bZIP) transcription factor gene family in six legume genomes
Source: BMC Genomics. 2015 Dec 10;16:1053. doi: 10.1186/s12864-015-2258-x (PMC4676100; doi:10.1186/s12864-015-2258-x)
Supplement: Additional file 2: — Position and pattern of introns within the basic and hinge regions of bZIP domains of the legume bZIP transcription factors. (PDF 1398 kb) [file 12864_2015_2258_MOESM2_ESM.pdf]

Additional file 2. Position and pattern of introns within the basic and hinge regions of bZIP domains of the legume bZIP transcription factors

Group	bZIP ID	Basic and hinge regions (before intron insertion site)	(after intron insertion site)	Pattern (a - d) of intron phase in Figure 2
	GmbZIP11	KVVERRQRRMIKNRESAARSRARKQ	AYTFEL	
	GmbZIP15	KTVERRQKRMIKNRESAARSRARKQ	AYTQEL	
	GmbZIP26	TLLDQRHARIIKNRESAVRSRARKQ	AYRKGL	
	GmbZIP29	KVIERRQRRMIKNRESAARSRARKQ	AYTMEL	
	GmbZIP32	KTVERRQKRMIKNRESAARSRARKQ	AYTTEL	
	GmbZIP36	KTVERRQKRMIKNRESAARSRARRQ	AYTQEL	
	GmbZIP38.1	KATLQKLRRMIKNRESAARSRERKQ	AYTSEL	
	GmbZIP38.2	KATLQKLRRMIKNRESAARSRERKQ	AYTSEL	
	GmbZIP38.3	KATLQKLRRMIKNRESAARSRERKQ	AYTSEL	
	GmbZIP38.4	KATLQKLRRMIKNRESAARSRERKQ	AYTSEL	
	GmbZIP42	TLQDQRHTRVIKNRESAVRSRARKQ	AYRKGL	
	GmbZIP44	KVIERRQRRMIKNRESAARSRARKQ	AYTMEL	
	GmbZIP48.1	KTVERRQKRMIKNRESAARSRARKQ	AYTTEL	
	GmbZIP48.2	KTVERRQKRMIKNRESAARSRARKQ	AYTTEL	
	GmbZIP50.1	KVVERRQRRMIKNRESAARSRARKQ	AYTFEL	
	GmbZIP50.2	KVVERRQRRMIKNRESAARSRARKQ	AYTFEL	
	GmbZIP50.3	KVVERRQRRMIKNRESAARSRARKQ	AYTFEL	
	GmbZIP51	KATLQKQRRMIKNRESAARSRERKQ	AYTSEL	
	GmbZIP54	KVTLQKQRRMIKNRESAARSRERKQ	AYTVEL	
	GmbZIP55.1	KTVERRQKRMIKNRESAARSRARKQ	AYTNEL	
	GmbZIP55.2	KTVERRQKRMIKNRESAARSRARKQ	AYTNEL	
	GmbZIP61	KVVERRQRRMIKNRESAARSRARKQ	AYTVEL	
	GmbZIP64	KAAQQRQRRMIKNRESAARSRERKQ	AYQVEL	
	GmbZIP84	VVVERRQRRMLKNRESAARSRARRQ	AYTVEL	
	GmbZIP87	KTIERKQKRMAKNRESVVRSRTKKQ	EHINKL	
	GmbZIP89.1	KTLERRLRRKIKNRESAARSRARKQ	AYHNEL	
	GmbZIP89.2	KTLERRLRRKIKNRESAARSRARKQ	AYHNEL	
A	GmbZIP89.3	KTLERRLRRKIKNRESAARSRARKQ	AYHNEL	a
	GmbZIP89.4	KTLERRLRRKIKNRESAARSRARKQ	AYHNEL	
	GmbZIP91.1	KVVERRQRRMIKNRESAARSRARKQ	AYTVEL	
	GmbZIP91.2	KVVERRQRRMIKNRESAARSRARKQ	AYTVEL	
	GmbZIP96	VVVERRQRRMLKNRESAARSRARRQ	AYTVEL	
	GmbZIP101	RTIERRQKRMAKNRESAGRSRAKKQ	EHINRL	
	GmbZIP106.1	KVTLQKQRRMIKNRESAARSRERKQ	AYTVEL	
	GmbZIP106.2	KVTLQKQRRMIKNRESAARSRERKQ	AYTVEL	
	GmbZIP107	KTVERRQKRMIKNRESAARSRARKQ	AYTNEL	
	GmbZIP123	KTVERRQKRMIKNRESAARSRARRQ	AYTQEL	
	GmbZIP124	KIVERRQKRMIKNRESAARSRARKQ	AYTQEL	
	GmbZIP129	KVVERRQRRMIKNRESAARSRARKQ	AYTVEL	
	GmbZIP135	KTLERRLRRKIKNRESAARSRARKQ	AYHNEL	
	MtbZIP9.1	KAAQQRQRRMIKNRESAARSRERKQ	AYQVEL	
	MtbZIP9.2	KAAQQRQRRMIKNRESAARSRERKQ	AYQVEL	
	MtbZIP9.3	KAAQQRQRRMIKNRESAARSRERKQ	AYQVEL	
	MtbZIP14	VVVERRQRRMLKNRESAARSRARRQ	AYTVEL	
	MtbZIP15.1	VLLDRKQRRMMKNRESAARSRARKQ	AYTIEL	
	MtbZIP15.2	VLLDRKQRRMMKNRESAARSRARKQ	AYTIEL	
	MtbZIP19	RTVERKQKRMIKNRESAARSRARKQ	AYTTEL	
	MtbZIP25.1	KVIERRQRRMIKNRESAARSRARKQ	AYTMEL	
	MtbZIP25.2	KVIERRQRRMIKNRESAARSRARKQ	AYTMEL	
	MtbZIP34	KGVERRQKRMIKNRESAARSRARKQ	AYTVEL	
	MtbZIP35	KAALQKQKRMIKNRESAARSRERKQ	AYTTEL	
	MtbZIP41.1	KALERRLRRKIKNRESAARSRARKQ	AYHNEL	
	MtbZIP41.2	KALERRLRRKIKNRESAARSRARKQ	AYHNEL	
	MtbZIP43	KTVERRQKRMIKNRESAARSRARRQ	AYTQEL	

A

a

MtbZIP48	KTVERRQKRMIKNRESAARSRARKQ AYTQEL
MtbZIP53	RVVERRQRRMIKNRESAARSRARKQ AYTVEL
MtbZIP61	KTVERRQKRMIKNRESAARSRARKQ AYTNEL
PvbZIP5	KTVERRQKRMIKNRESAARSRARKQ AYTHEL
PvbZIP10	KVVERRQRRMIKNRESAARSRARKQ AYTVEL
PvbZIP16	KATLQKQRRMIKNRESAARSRERKQ AYTSEL
PvbZIP22	SRDMRNARLMKNRESAARSRARKQA YLIELK
PvbZIP24.1	KVVERRQRRMIKNRESAARSRARKQ AYTFEL
PvbZIP24.2	KVVERRQRRMIKNRESAARSRARKQ AYTFEL
PvbZIP26	KATIQKQRRMIKNRESAARSRERKQ AYTMEL
PvbZIP27	RTVERRHKRMIKNRESAARSRARRQ AYTQEL
PvbZIP29	KTFDRRQKRMIKNRESAARSRARKQ AYTIEL
PvbZIP33	EVIERRQRRMLKNRESAARSRARRQ AYTVEL
PvbZIP35	KTLERRLRRKIKNRESAARSRARKQ AYHNEL
PvbZIP42	KAAQQRQRRMIKNRESAARSRERKQ AYQVEL
PvbZIP45	KVVERRQRRMIKNRESAARSRARKL AYTVEL
PvbZIP51	KAVERRQKRMAKNRESAARSRAKKQ EHIHRL
PvbZIP54	HHQDQRHVRIMKNRESAVRSRARKQ AYRRGL
PvbZIP57.1	KVIERRQRRMIKNRESAARSRARKQ AYTMEL
PvbZIP57.2	KVIERRQRRMIKNRESAARSRARKQ AYTMEL
PvbZIP71.1	KTVERRQKRMIKNRESAARSRARKQ AYTNEL
PvbZIP71.2	KTVERRQKRMIKNRESAARSRARKQ AYTNEL
PvbZIP71.3	KTVERRQKRMIKNRESAARSRARKQ AYTNEL
CabZIP4	VVAERRQRRMLKNRESAARSRARRQ AYTVEL
CabZIP6	KTVERRQKRMIKNRESAARSRARRQ AYTQEL
CabZIP9	KALERRLRRKIKNRESAARSRARKQ AYHNEL
CabZIP11	KTVERRQKRMIKNRESAARSRARKQ AYTQEL
CabZIP16	RVVERRQRRMIKNRESAARSRARKQ AYTVEL
CabZIP23	KAAQQRQRRMIKNRESAARSRERKQ AYQVEL
CabZIP30	KTVERKQKRMIKNRESAARSRARKQ AYTTEL
CabZIP34	KVIERRQRRMIKNRESAARSRARKQ AYTMEL
CabZIP39	KAALARQKRLIKNRESAAKSRAKKE RYVSGL
CabZIP40	KVVERRQRRMIKNRESAARSRARKQ AYTVEL
CabZIP52	KTVERRQKRMIKNRESAARSRARKQ AYTNEL
CcbZIP10	KVVERRQRRMIKNRESAARSRARKQ AYTVEL
CcbZIP20	KTVERRQKRMIKNRESAARSRARKQ AYTQEL
CcbZIP24	KVIERRQRRMIKNRESAARSRARKQLKNSVSHFYFH AYTMEL
CcbZIP26	RTVERRQKRMIKNRESAARSRARKQ AYTTEL
CcbZIP35	KAAQQRQRRMIKNRESAARSRERKQ AYQVEL
CcbZIP37	KATLQKQRRMIKNRESAARSRERKQ AYTVEL
CcbZIP50	KTLERRLRRKIKNRESAARSRARKQ AYHNEL
CcbZIP52	THQDQRHMRILRNRESAVRSRARKQ AYRKGL
CcbZIP53	KATLQKQRRMIKNRESAARSRERKQ AYTSEL
CcbZIP56	KTVERRQKRMIKNRESAARSRARKQ AYTQEL
CcbZIP59	KVVERRQRRMIKNRESAARSRARKQ AYTFEL
CcbZIP61	KVVERRQRRMIKNRESAARSRARKH AYTVEL
LjbZIP11	GLGDRRNKRMIKNRESAARSRARKQ AYTNEL
LjbZIP14	KTVERRQKRMIKNRESAARSRARKQ AYTNEL
LjbZIP18	KATLQKQRRMIKNRESAARSRERKQ AYTTEL
LjbZIP23	KAAQQRQRRMIKNRESAARSRERKQ AYQVEL
LjbZIP26	KATLQKQRRMIKNRESAARSRERKQ AYTLEL
LjbZIP29	KTVERRQKRMIKNRESAARSRARKQ AYTTEL

Group	Name	Sequence		Label
B	GmbZIP18	DEDEKRKARLMRNRESAQLSRQRKKHYVEEL		d
	GmbZIP125	DEDEKRKARLMRNRESAQLSRQRKKHYVEEL		
	MtbZIP49	DEDEKRKARLMRNRESAQLSRQRKKHYVEEL		
	PvbZIP6	DDDEKRKARLMRNRESAQLSRQRKKHYVEEL		
	CabZIP12	DEDEKRKARLMRNRESAQLSRQRKKHYVEEL		
	CcbZIP21	DDDEKRKARLMRNRESAQLSRQRKKHYVEEL		
	LjbZIP5	DDDEKRKARLMRNRESAQLSRQRKKHYVEEL		
C	GmbZIP23	PVDAKRVRR	MLSNRESARRSRRRKQAHLTEL	c
	GmbZIP31.1	PADMKRLRR	KVSNRDSARRSRRRKQAQLSEL	
	GmbZIP31.2	PADMKRLRR	KVSNRDSARRSRRRKQAQLSEL	
	GmbZIP31.3	PADMKRLRR	KVSNRDSARRSRRRKQAQLSEL	
	GmbZIP46.1	PADMKRLRR	KVSNRDSARRSRRRKQAQLSDL	
	GmbZIP46.2	PADMKRLRR	KVSNRDSARRSRRRKQAQLSDL	
	GmbZIP63	PADVKRVRR	MLSNRESARRSRRRKQAHLTDL	
	GmbZIP115.1	AIDVKRLRR	KVSNRESARRSRRRKQAHLADL	
	GmbZIP115.2	AIDVKRLRR	KVSNRESARRSRRRKQAHLADL	
	GmbZIP131	PVDAKRVRR	MLSNRESARRSRRRKQAHLTEL	
	GmbZIP134	PVDAKRVRR	MLSNRESDRCSRRRKQTHLTEL	
	GmbZIP137.1	PADVKRVRR	MLSNRESARRSRRRKQAHLTDL	
	GmbZIP137.2	PADVKRVRR	MLSNRESARRSRRRKQAHLTDL	
	MtbZIP1.1	PVDMKRLRR	KVSNRESARRSRRRKQAHLADL	
	MtbZIP1.2	PVDMKRLRR	KVSNRESARRSRRRKQAHLADL	
	MtbZIP7	PTDVKRVRR	MLSNRESARRSRRRKQAHLTEL	
	MtbZIP27	PVDMKRQRR	KDSNCESARRSRWRKQAHLSEL	
	MtbZIP55	PTDAKRVRR	MLSNRESARRSRRRKQAHLTEL	
	MtbZIP56	PSDAKRLKR	MLQNRESAKRSRDRKIAKNTEL	
	PvbZIP4	ERNEKAQVK	MRTKNSNRESARRSRRRKQAHL	
	PvbZIP38	PTDAKRVRR	MLSNRESARRSRRRKQAHLSDL	
	PvbZIP43	PADAKRVRR	MLSNRESARRSRRRKQAHLTDL	
	PvbZIP59	AVELKRLRR	KVSNRNSARRSRRRKQAQLADL	
	CabZIP18	RADAKRVRR	MLSNRESARRSRRRKQAHLTDL	
	CabZIP21	PADVKRVRR	MLSNRESARRSRRRKQAHLTEL	
	CabZIP26	PIDMKRLRR	KVSNRESARRSRRRKQAHLTDL	
	CabZIP33	PLHMKRLRR	KVSNRDSARRSRQRKQAHLAEL	
	CcbZIP5	PADAKRVRR	MLSNRESARRSRRRKQAHLTDL	
	CcbZIP7	PLEVKRLKR	MDSNRESARRSRRRKQAHLADL	
	CcbZIP11	PLDAKRVRR	MLSNRESARRSRRRKQAHLTEL	
	CcbZIP55	AIDIKRLRR	KFSNRESARRSRRRKQAHLADL	
	LjbZIP3	PQDVKRLRR	KVSNRESARRSRRRKQAHLAEL	
	LjbZIP21	PSDAKRVRR	MLSNRESARRSRRRKQAHLTDL	

D

GmbZIP3.1	PGDQK	TLRRLAQNREAARKSRLRKK	AYVQQL
GmbZIP3.2	DDIIP	TLRRLAQNREAARKSRLRKK	AYVQQL
GmbZIP9.1	PGDQK	TLRRLAQNREAARKSRLRKK	AYVQQL
GmbZIP9.2	KDATK	TLRRLAQNREAARKSRLRKK	AYVQQL
GmbZIP13.1	TPDPK	TLRRLAQNREAARKSRLRKK	AYVQQL
GmbZIP13.2	TPDPK	TLRRLAQNREAARKSRLRKK	AYVQQL
GmbZIP19.1	KSDQK	TLRRLAQNREAARKSRLRKK	AYVQQL
GmbZIP19.2	KSDQK	TLRRLAQNREAARKSRLRKK	AYVQQL
GmbZIP20	TPDPK	TLRRLAQNREAARKSRLRKK	AYVQQL
GmbZIP34.1	NVENK	MLRRLAQNREAARKSRLRKK	AYVKQL
GmbZIP34.2	NVENK	MLRRLAQNREAARKSRLRKK	AYVKQL
GmbZIP40.1	RLTDK	TQRRLAQNREAARKSRLRKK	AYVQQL
GmbZIP40.2	RLTDK	TQRRLAQNREAARKSRLRKK	AYVQQL
GmbZIP40.3	RLTDK	TQRRLAQNREAARKSRLRKK	AYVQQL
GmbZIP40.4	RLTDK	TQRRLAQNREAARKSRLRKK	AYVQQL
GmbZIP53.1	RLTDK	TQRRLAQNREAARKSRLRKK	AYVQQL
GmbZIP53.2	RLTDK	TQRRLAQNREAARKSRLRKK	AYVQQL
GmbZIP53.3	RLTDK	TQRRLAQNREAARKSRLRKK	AYVQQL
GmbZIP53.4	RLTDK	TQRRLAQNREAARKSRLRKK	AYVQQL
GmbZIP53.5	RLTDK	TQRRLAQNREAARKSRLRKK	AYVQQL
GmbZIP53.6	RLTDK	TQRRLAQNREAARKSRLRKK	AYVQQL
GmbZIP62	TPDPK	TLRRLAQNREAARKSRLRKK	AYVQQL
GmbZIP66	AEDQK	TVRRLAQNREAARKSRLRKK	AYVQQL
GmbZIP67.1	KSDQK	SLRRLAQNREAARKSRLRKK	AYVQQL
GmbZIP67.2	KSDQK	SLRRLAQNREAARKSRLRKK	AYVQQL
GmbZIP67.3	KSDQK	SLRRLAQNREAARKSRLRKK	AYVQQL
GmbZIP74.1	PLDAK	TLRRLAQNREAARKSRLRKK	AYVQQL
GmbZIP74.2	PLDAK	TLRRLAQNREAARKSRLRKK	AYVQQL
GmbZIP77.1	KPTDK	IQRRLAQNREAARKSRLRKK	AYVQQL
GmbZIP77.2	KPTDK	IQRRLAQNREAARKSRLRKK	AYVQQL
GmbZIP77.3	KPTDK	IQRRLAQNREAARKSRLRKK	AYVQQL
GmbZIP77.4	KPTDK	IQRRLAQNREAARKSRLRKK	AYVQQL
GmbZIP77.5	KPTDK	IQRRLAQNREAARKSRLRKK	AYVQQL
GmbZIP82.1	PLDAK	TLRRLAQNREAARKSRLRKK	AYVQQL
GmbZIP82.2	PLDAK	TLRRLAQNREAARKSRLRKK	AYVQQL
GmbZIP82.3	PLDAK	TLRRLAQNREAARKSRLRKK	AYVQQL
GmbZIP82.4	PLDAK	TLRRLAQNREAARKSRLRKK	AYVQQL
GmbZIP82.5	PLDAK	TLRRLAQNREAARKSRLRKK	AYVQQL
GmbZIP82.6	PLDAK	TLRRLAQNREAARKSRLRKK	AYVQQL
GmbZIP85.1	PLDAK	TLRRLAQNREAARKSRLRKK	AYVQQL
GmbZIP85.2	PLDAK	TLRRLAQNREAARKSRLRKK	AYVQQL
GmbZIP85.3	PLDAK	TLRRLAQNREAARKSRLRKK	AYVQQL
GmbZIP85.4	PLDAK	TLRRLAQNREAARKSRLRKK	AYVQQL
GmbZIP85.5	PLDAK	TLRRLAQNREAARKSRLRKK	AYVQQL
GmbZIP85.6	PLDAK	TLRRLAQNREAARKSRLRKK	AYVQQL
GmbZIP88.1	KADTK	ALRRQAQNREAARKCRLRKK	AYVQQL
GmbZIP88.2	KADTK	ALRRQAQNREAARKCRLRKK	AYVQQL
GmbZIP88.3	KADTK	ALRRQAQNREAARKCRLRKK	AYVQQL
GmbZIP88.4	KADTK	ALRRQAQNREAARKCRLRKK	AYVQQL
GmbZIP88.5	KADTK	ALRRQAQNREAARKCRLRKK	AYVQQL
GmbZIP92	KSDQK	TLRRLAQNREAARKSRLRKK	AYVQQL
GmbZIP95.1	PLDAK	ALRRLAQNREAARKSRLRKK	AYVQQL
GmbZIP95.2	PLDAK	ALRRLAQNREAARKSRLRKK	AYVQQL

b

D

GmbZIP95.3	PLDAK	ALRRLAQNREAARKSRLRKK	AYVQQL
GmbZIP100.1	KADTK	ALRRQAQNREAARKCRLRKK	AYVQQL
GmbZIP100.2	KADTK	ALRRQAQNREAARKCRLRKK	AYVQQL
GmbZIP100.3	KADTK	ALRRQAQNREAARKCRLRKK	AYVQQL
GmbZIP100.4	KADTK	ALRRQAQNREAARKCRLRKK	AYVQQL
GmbZIP108.1	KSDQK	TLRRLAQNREAARKSRLRKK	AYVQQL
GmbZIP108.2	KSDQK	TLRRLAQNREAARKSRLRKK	AYVQQL
GmbZIP118.1	KPTDK	IQRRLAQNREAARKSRLRKK	AYVQQL
GmbZIP118.2	KPTDK	IQRRLAQNREAARKSRLRKK	AYVQQL
GmbZIP118.3	KPTDK	IQRRLAQNREAARKSRLRKK	AYVQQL
GmbZIP118.4	KPTDK	IQRRLAQNREAARKSRLRKK	AYVQQL
GmbZIP118.5	KPTDK	IQRRLAQNREAARKSRLRKK	AYVQQL
GmbZIP126.1	KSDQK	TLRRLAQNREAARKSRLRKK	AYVQQL
GmbZIP126.2	KSDQK	TLRRLAQNREAARKSRLRKK	AYVQQL
GmbZIP126.3	KSDQK	TLRRLAQNREAARKSRLRKK	AYVQQL
GmbZIP127	TPDPK	TLRRLAQNREAARKSRLRKK	AYVQQL
GmbZIP136.1	AEDQK	TVRRLAQNREAAKKSRLRKK	AYVQQL
GmbZIP136.2	AEDQK	TVRRLAQNREAAKKSRLRKK	AYVQQL
GmbZIP138.1	KSDQK	TLRRLAQNREAARKSRLRKK	AYVQQL
GmbZIP138.2	KSDQK	TLRRLAQNREAARKSRLRKK	AYVQQL
GmbZIP138.3	KSDQK	TLRRLAQNREAARKSRLRKK	AYVQQL
MtbZIP10	DEHKT	LRRLMQNREAARKSRLRKKA	YVQQL
MtbZIP16.1	PLDAK	TLRRLAQNREAARKSRLRKK	AYVQQL
MtbZIP16.2	PLDAK	TLRRLAQNREAARKSRLRKK	AYVQQL
MtbZIP21	SDEHK	TLRRLMQNREAARKSRLRKK	AYVQQL
MtbZIP23	RPIDK	IQRRLAQNREAARKSRLRKK	AYVQQL
MtbZIP30.1	TLDPK	TLRRLAQNREAAKKSRLRKK	AYVQQL
MtbZIP30.2	TLDPK	TLRRLAQNREAAKKSRLRKK	AYVQQL
MtbZIP30.3	TLDPK	TLRRLAQNREAAKKSRLRKK	AYVQQL
MtbZIP39.1	AEDHK	TLRRLAQNREAARKSRLRKK	AYVQQL
MtbZIP39.2	AEDHK	TLRRLAQNREAARKSRLRKK	AYVQQL
MtbZIP39.3	AEDHK	TLRRLAQNREAARKSRLRKK	AYVQQL
MtbZIP39.4	AEDHK	TLRRLAQNREAARKSRLRKK	AYVQQL
MtbZIP50.1	KSDQK	TLRRLAQNREAARKSRLRKK	AYVQQL
MtbZIP50.2	KSDQK	TLRRLAQNREAARKSRLRKK	AYVQQL
MtbZIP50.3	KSDQK	TLRRLAQNREAARKSRLRKK	AYVQQL
MtbZIP51.1	TPDPK	ILRRLAQNREAARKSRLRKK	AYIQQL
MtbZIP51.2	TPDPK	ILRRLAQNREAARKSRLRKK	AYIQQL
MtbZIP51.3	TPDPK	ILRRLAQNREAARKSRLRKK	AYIQQL
MtbZIP60.1	KSDQK	TLRRLAQNREAARKSRLRKK	AYVQQL
MtbZIP60.2	KSDQK	TLRRLAQNREAARKSRLRKK	AYVQQL
MtbZIP60.3	KSDQK	TLRRLAQNREAARKSRLRKK	AYVQQL
MtbZIP60.4	KSDQK	TLRRLAQNREAARKSRLRKK	AYVQQL
MtbZIP60.5	KSDQK	TLRRLAQNREAARKSRLRKK	AYVQQL
MtbZIP65	TPDPK	TLRRLAQNREAARKSRLRKK	AYVQQL
PvbZIP7	KSDQK	TLRRLAQNREAARKSRLRKK	AYVQQL
PvbZIP8.1	TPDPK	TLRRLAQNREAARKSRLRKK	AYVQQL
PvbZIP8.2	TPDPK	TLRRLAQNREAARKSRLRKK	AYVQQL
PvbZIP12.1	KPTDK	IQRRLAQNREAARKSRLRKK	AYVQQL
PvbZIP12.2	KPTDK	IQRRLAQNREAARKSRLRKK	AYVQQL
PvbZIP18	PEDQK	TLRRLAQNREAAKKSRLRKK	AYVQQL
PvbZIP32	PLDAK	TLRRLAQNREAARKSRLRKK	AYVQQL
PvbZIP40	KSDQK	TLRRLAQNREAARKSRLRKK	AYVQQL
PvbZIP41	PEDQK	TVRRLAQNREAARKSRLRKK	AYVQQL
PvbZIP47.1	TPDPK	TLRRLAQNREAARKSRLRKK	AYVQQL
PvbZIP47.2	TPDPK	TLRRLAQNREAARKSRLRKK	AYVQQL
PvbZIP50	KADSK	VLRRQAQNREAARKCRLRKK	AYVQQL
PvbZIP55	KDVNK	MRRRLAQNREAARKSRLRKK	AYVKQL
PvbZIP69.1	PLDAK	TLRRLAQNREAARKSRLRKK	AYVQQL
PvbZIP69.2	PLDAK	TLRRLAQNREAARKSRLRKK	AYVQQL
PvbZIP72	KSDQK	TLRRLAQNREAARKSRLRKK	AYVQQL
CabZIP3	PLDAK	TLRRLAQNREAARKSRLRKK	AYVQQL
CabZIP13	KSDQK	TLRRLAQNREAARKSRLRKK	AYVQQL
CabZIP14	TPDAK	TLRRLAQNREAARKSRLRKK	AYIQQL
CabZIP25	AEEHK	SLRRLAQNREAARKSRLRKK	AYVQQL
CabZIP27	KSDQK	TLRRLAQNREAARKSRLRKK	AYVQQL
CabZIP35	RPIDK	IQRRLAQNREAARKSRLRKK	AYVQQL
CabZIP37	KLTDK	MQRRLAQNREAARKSRLKKK	AYVQQL
CabZIP43	TLDPK	TLRRLAQNREAAKKSRLRKK	AYVQQL
CabZIP51	KSDQK	TLRRLAQNREAARKSRLRKK	AYVQQL
CabZIP54	KDDSK	VLRRLAQNREAARKSRLRKK	AYVQQL
CabZIP56	AEDHK	TLRRLAQNREAARKSRLRKK	AYVQQL
CcbZIP1	KPTDK	IQRRLAQNREAARKSRLRKK	AYVQQL
CcbZIP4	TPDPK	TLRRLAQNREAARKSRLRKK	AYVQQL
CcbZIP6	AEDQK	TIRRLAQNREAARKSRLRKK	AYVQQL
CcbZIP8	KSDQK	TLRRLAQNREAARKSRLRKK	AYVQQL
CcbZIP9	TPDPK	TLRRLAQNREAARKSRLRKK	AYVQQL
CcbZIP15	QLDAK	ALRRLAQNREAARKSRLRKK	AYVQQL
CcbZIP22	KSDQK	TLRRLAQNREAARKSRLRKK	AYVQQL
CcbZIP23	KSDQK	TLRRLAQNREAARKSRLKKK	AYVQQL
CcbZIP38	KADRK	AQRRLAQNREAARKCRLRKK	AYVQQL
CcbZIP48	PLDAK	TLRRLAQNREAARKSRLRKK	AYVQQL
CcbZIP54	VEDQK	TLRRLAQNREAARKSRLRKK	AYVQQL
LjbZIP2	RPIDK	IQRRLAQNREAARKSRLRKK	AYVQQL
LjbZIP6	KTDQK	TLRRLAQNREAARKSRLRKK	AYVQQL
LjbZIP8	PEDQK	ILRRLAQNREAAKKSRLRKK	AYVQQL

b

E	GmbZIP47.1	ITDPKRVKR ILANRQSAQRSRVRKLQYISEL	c
	GmbZIP47.2	ITDPKRVKR ILANRQSAQRSRVRKLQYISEL	
	GmbZIP83	ITDPKRVKR ILANRQSAQRSRVRKLQYISEL	
	GmbZIP86	ITDPKRVKR ILANRQSAQRSRVRKLQYISEL	
	GmbZIP93	VVDPKRVKR ILANRQSAQRSRVRKLQYISEL	
	GmbZIP94.1	DPKRVKR ILANRQSAQRSRVRKLQYISEL	
	GmbZIP94.2	ITDPKRVKR ILANRQSAQRSRVRKLQYISEL	
	MtbZIP17.1	ITDPKRVKR ILANRQSAQRSRVRKLQYISEL	
	MtbZIP17.2	ITDPKRVKR ILANRQSAQRSRVRKLQYISEL	
	MtbZIP33	ITDPKRVKR ILANRQSAQRSRVRKLQYISEL	
	PvbZIP30	VVDPKRVKR ILANRQSAQRSRVRKLQYISEL	
	PvbZIP31	ITDPKRVKR ILANRQSAQRSRVRKLQYISEL	
	PvbZIP70.1	ITDPKRVKR ILANRQSAQRSRVRKLQYISEL	
	PvbZIP70.2	ITDPKRVKR ILANRQSAQRSRVRKLQYISEL	
	CabZIP2	ITDPKRVKR ILANRQSAQRSRVRKLQYISEL	
	CabZIP5	VVDPKRVKR ILANRQSAQRSRVRKLQYISEL	
	CabZIP41	ITDPKRVKR ILANRQSAQRSRVRKLQYISEL	
	CcbZIP16	ITDPKRVKR ILANRQSAQRSRVRKLQYISEL	
	CcbZIP34	ITDPKRVKR ILANRQSAQRSRVRKLQYISEL	
	CcbZIP42	VVDPKRVKR ILANRQSAQRSRVRKLQYISEL	
	LjbZIP16	ITDPKRVKR ILANRQSAQRSRVRKLQYISEL	
F	GmbZIP73.1	KHSNSKQKRPSGNREAVRKYREKKKAHTAYL	d
	GmbZIP73.2	KHSNSKQKRPSGNREAVRKYREKKKAHTAYL	
	GmbZIP73.3	KHSNSKQKRPSGNREAVRKYREKKKAHTAYL	
	PvbZIP64.1	SAEKKSKKRPLGNKEAVRKYREKKKARAASL	
	PvbZIP64.2	SAEKKSKKRPLGNKEAVRKYREKKKARAASL	
	CabZIP42	SAEKKSKKRPTGNKEAVRKYREKKKARAASL	

G

GmbZIP1	ERELKRQRRKQSNRESARRSRLRKQ	AECDEL
GmbZIP4.1	DRELKKQKRKQSNRESARRSRLRKQ	AECEEL
GmbZIP4.2	DRELKKQKRKQSNRESARRSRLRKQ	AECEEL
GmbZIP4.3	DRELKKQKRKQSNRESARRSRLRKQ	AECEEL
GmbZIP4.4	DRELKKQKRKQSNRESARRSRLRKQ	AECEEL
GmbZIP4.5	DRELKKQKRKQSNRESARRSRLRKQ	AECEEL
GmbZIP4.6	DRELKKQKRKQSNRESARRSRLRKQ	AECEEL
GmbZIP7.1	ERELKRQKRKQSNRESARRSRLRKQ	AECEEL
GmbZIP7.2	ERELKRQKRKQSNRESARRSRLRKQ	AECEEL
GmbZIP7.3	ERELKRQKRKQSNRESARRSRLRKQ	AECEEL
GmbZIP7.4	ERELKRQKRKQSNRESARRSRLRKQ	AECEEL
GmbZIP24.1	ERELKRERRKQSNRESARRSRLRKQ	AETEEL
GmbZIP24.2	ERELKRERRKQSNRESARRSRLRKQ	AETEEL
GmbZIP24.3	ERELKRERRKQSNRESARRSRLRKQ	AETEEL
GmbZIP24.4	ERELKRERRKQSNRESARRSRLRKQ	AETEEL
GmbZIP24.5	ERELKRERRKQSNRESARRSRLRKQ	AETEEL
GmbZIP24.6	ERELKRERRKQSNRESARRSRLRKQ	AETEEL
GmbZIP24.7	ERELKRERRKQSNRESARRSRLRKQ	AETEEL
GmbZIP49.1	ERELKRERRKQSNRESARRSRLRKQ	AETEEL
GmbZIP49.2	ERELKRERRKQSNRESARRSRLRKQ	AETEEL
GmbZIP57.1	ERELKRQRRKQSNRESARRSRLRKQ	AECDEL
GmbZIP57.2	ERELKRQRRKQSNRESARRSRLRKQ	AECDEL
GmbZIP57.3	ERELKRQRRKQSNRESARRSRLRKQ	AECDEL
GmbZIP59	ERELKRQRRKQSNRESARRSRLRKQ	AECDEL
GmbZIP69.1	ERELKKQKRKQSNRESARRSRLRKQ	AECEEL
GmbZIP69.2	ERELKKQKRKQSNRESARRSRLRKQ	AECEEL
GmbZIP69.3	ERELKKQKRKQSNRESARRSRLRKQ	AECEEL
GmbZIP69.4	ERELKKQKRKQSNRESARRSRLRKQ	AECEEL
GmbZIP80	TAEEKEQRRKQQKKIASKRSRMKIK	MEREKL
GmbZIP109	ERELKRERRKQSNRESARRSRLRKQ	AETEEL
GmbZIP111	ERELKRQRRKQSNRESARRSRLRKQ	AECDEL
GmbZIP112.1	ERELKRQKRKQSNRESARRSRLRKQ	AECEEL
GmbZIP112.2	ERELKRQKRKQSNRESARRSRLRKQ	AECEEL
GmbZIP112.3	ERELKRQKRKQSNRESARRSRLRKQ	AECEEL
GmbZIP112.4	ERELKRQKRKQSNRESARRSRLRKQ	AECEEL
GmbZIP112.5	ERELKRQKRKQSNRESARRSRLRKQ	AECEEL
GmbZIP132.1	ERELKRERRKQSNRESARRSRLRKQ	AETEEL
GmbZIP132.2	ERELKRERRKQSNRESARRSRLRKQ	AETEEL
GmbZIP133.1	KDEIRRERKRQSNRESARRSRMRKE	KECEEL
GmbZIP133.2	KDEIRRERKRQSNRESARRSRMRKE	KECEEL
MtbZIP5	GDDIRKERKRLSNRKSAKRSKIKKQ	QECEEL
MtbZIP12	DDERRKERKRLSNRKSAKRSKIKKQ	KEYEEQ
MtbZIP13	GDEIRKERKRLSNRKSAKRSKIKKQ	KECEEL
MtbZIP38.1	ERELKRQRRKQSNRESARRSRLRKQ	AECDEL
MtbZIP38.2	ERELKRQRRKQSNRESARRSRLRKQ	AECDEL
MtbZIP57.1	ERELKRERRKQSNRESARRSRLRKQ	AEAEEL
MtbZIP57.2	ERELKRERRKQSNRESARRSRLRKQ	AEAEEL
MtbZIP57.3	ERELKRERRKQSNRESARRSRLRKQ	AEAEEL
MtbZIP57.4	ERELKRERRKQSNRESARRSRLRKQ	AEAEEL
MtbZIP57.5	ERELKRERRKQSNRESARRSRLRKQ	AEAEEL
MtbZIP57.6	ERELKRERRKQSNRESARRSRLRKQ	AEAEEL
MtbZIP59	ERELKRERRKQSNRESARRSRLRKQ	AEAEEL
MtbZIP62.1	ERELKRQRRKQSNRESARRSRLRKQ	AECDEL

a

G

GmbZIP1	ERELKRQRRKQSNRESARRSRLRKQ AECDEL
GmbZIP4.1	DRELKKQKRKQSNRESARRSRLRKQ AECEEL
GmbZIP4.2	DRELKKQKRKQSNRESARRSRLRKQ AECEEL
GmbZIP4.3	DRELKKQKRKQSNRESARRSRLRKQ AECEEL
GmbZIP4.4	DRELKKQKRKQSNRESARRSRLRKQ AECEEL
GmbZIP4.5	DRELKKQKRKQSNRESARRSRLRKQ AECEEL
GmbZIP4.6	DRELKKQKRKQSNRESARRSRLRKQ AECEEL
GmbZIP7.1	ERELKRQKRKQSNRESARRSRLRKQ AECEEL
GmbZIP7.2	ERELKRQKRKQSNRESARRSRLRKQ AECEEL
GmbZIP7.3	ERELKRQKRKQSNRESARRSRLRKQ AECEEL
GmbZIP7.4	ERELKRQKRKQSNRESARRSRLRKQ AECEEL
GmbZIP24.1	ERELKRERRKQSNRESARRSRLRKQ AETEEL
GmbZIP24.2	ERELKRERRKQSNRESARRSRLRKQ AETEEL
GmbZIP24.3	ERELKRERRKQSNRESARRSRLRKQ AETEEL
GmbZIP24.4	ERELKRERRKQSNRESARRSRLRKQ AETEEL
GmbZIP24.5	ERELKRERRKQSNRESARRSRLRKQ AETEEL
GmbZIP24.6	ERELKRERRKQSNRESARRSRLRKQ AETEEL
GmbZIP24.7	ERELKRERRKQSNRESARRSRLRKQ AETEEL
GmbZIP49.1	ERELKRERRKQSNRESARRSRLRKQ AETEEL
GmbZIP49.2	ERELKRERRKQSNRESARRSRLRKQ AETEEL
GmbZIP57.1	ERELKRQRRKQSNRESARRSRLRKQ AECDEL
GmbZIP57.2	ERELKRQRRKQSNRESARRSRLRKQ AECDEL
GmbZIP57.3	ERELKRQRRKQSNRESARRSRLRKQ AECDEL
GmbZIP59	ERELKRQRRKQSNRESARRSRLRKQ AECDEL
GmbZIP69.1	ERELKKQKRKQSNRESARRSRLRKQ AECEEL
GmbZIP69.2	ERELKKQKRKQSNRESARRSRLRKQ AECEEL
GmbZIP69.3	ERELKKQKRKQSNRESARRSRLRKQ AECEEL
GmbZIP69.4	ERELKKQKRKQSNRESARRSRLRKQ AECEEL
GmbZIP80	TAEEKEQRRKQQKKIASKRSRMKIK MEREKL
GmbZIP109	ERELKRERRKQSNRESARRSRLRKQ AETEEL
GmbZIP111	ERELKRQRRKQSNRESARRSRLRKQ AECDEL
GmbZIP112.1	ERELKRQKRKQSNRESARRSRLRKQ AECEEL
GmbZIP112.2	ERELKRQKRKQSNRESARRSRLRKQ AECEEL
GmbZIP112.3	ERELKRQKRKQSNRESARRSRLRKQ AECEEL
GmbZIP112.4	ERELKRQKRKQSNRESARRSRLRKQ AECEEL
GmbZIP112.5	ERELKRQKRKQSNRESARRSRLRKQ AECEEL
GmbZIP132.1	ERELKRERRKQSNRESARRSRLRKQ AETEEL
GmbZIP132.2	ERELKRERRKQSNRESARRSRLRKQ AETEEL
GmbZIP133.1	KDEIRRERKRQSNRESARRSRMRKE KECEEL
GmbZIP133.2	KDEIRRERKRQSNRESARRSRMRKE KECEEL
MtbZIP5	GDDIRKERKRLSNRKSAKRSKIKKQ QECEEL
MtbZIP12	DDERRKERKRLSNRKSAKRSKIKKQ KEYEEQ
MtbZIP13	GDEIRKERKRLSNRKSAKRSKIKKQ KECEEL
MtbZIP38.1	ERELKRQRRKQSNRESARRSRLRKQ AECDEL
MtbZIP38.2	ERELKRQRRKQSNRESARRSRLRKQ AECDEL
MtbZIP57.1	ERELKRERRKQSNRESARRSRLRKQ AEAEEL
MtbZIP57.2	ERELKRERRKQSNRESARRSRLRKQ AEAEEL
MtbZIP57.3	ERELKRERRKQSNRESARRSRLRKQ AEAEEL
MtbZIP57.4	ERELKRERRKQSNRESARRSRLRKQ AEAEEL
MtbZIP57.5	ERELKRERRKQSNRESARRSRLRKQ AEAEEL
MtbZIP57.6	ERELKRERRKQSNRESARRSRLRKQ AEAEEL
MtbZIP59	ERELKRERRKQSNRESARRSRLRKQ AEAEEL
MtbZIP62.1	ERELKRQRRKQSNRESARRSRLRKQ AECDEL

a

G

MtbZIP62.2	ERELKRQRRKQSNRESARRSRLRKQ	AECDEL
MtbZIP63.1	DRELKRQKRKQSNRESARRSRLRKQ	AECEEL
MtbZIP63.2	DRELKRQKRKQSNRESARRSRLRKQ	AECEEL
MtbZIP63.3	DRELKRQKRKQSNRESARRSRLRKQ	AECEEL
MtbZIP63.4	DRELKRQKRKQSNRESARRSRLRKQ	AECEEL
PvbZIP15	EREIKRQRRKQSNRESARRSRLRKQ	AECDEL
PvbZIP21	ERELKRQKRKQSNRESARRSRLRKQ	AECEDL
PvbZIP23	ERELKRQRRKQSNRESARRSRLRKQ	AECDEL
PvbZIP39.1	ERELKRERRKQSNRESARRSRLRKQ	AETEEL
PvbZIP39.2	ERELKRERRKQSNRESARRSRLRKQ	AETEEL
PvbZIP39.3	ERELKRERRKQSNRESARRSRLRKQ	AETEEL
PvbZIP39.4	ERELKRERRKQSNRESARRSRLRKQ	AETEEL
PvbZIP63.1	ERELKRERRKQSNRESARRSRLRKQ	AETEEL
PvbZIP63.2	ERELKRERRKQSNRESARRSRLRKQ	AETEEL
PvbZIP67	SDEAKEQRRRQSKKKSAKRSRLKMK	VERERL
CabZIP19	ERELKRERRKQSNRESARRSRLRKQ	AEAEEL
CabZIP48	DRELKRQKRKQSNRESARRSRLRKQ	AECEEL
CabZIP49	ERELKRQRRKQSNRESARRSRLRKQ	AECDEL
CabZIP50	ERELKRERRKQSNRESARRSRLRKQ	AEAEEL
CabZIP53	ERELKRQRRKQSNRESARRSRLRKQ	AECDEL
CcbZIP12	ERELKRERRKQSNRESARRSRLRKQ	AETEEL
CcbZIP19	ERELKRQRRKQSNRESARRSRLRKQ	AECDEL
CcbZIP29	ERELKRERRKQSNRESARRSRLRKQ	AEAEEL
CcbZIP43	ERELKRQKRKQSNRESARRSRLRKQ	AECEEL
CcbZIP46	ERELKRQRRKQSNRESARRSRLRKQ	AECDEL
LjbZIP7	DRELKRERRKQSNRESARRSRLRKQ	AEAEEL
LjbZIP10	ERELKRQRRKQSNRESARRSRLRKQ	AECDEL
LjbZIP15	DRELKRERRKQSNRESARRSRLRKQ	AETEEL
LjbZIP31	ERELKRQRRKQSNRESARRSRLRKQ	AECDEL

a

H

GmbZIP17.1	DKEHRRLKR	LLRNRVSAQQARERKKVYVNDL
GmbZIP17.2	DKEHRRLKR	LLRNRVSAQQARERKKVYVNDL
GmbZIP58	DKESKRLKR	LLRNRVSAQQARERKKAYLIDL
GmbZIP110	DKEHRRLKR	LLRNRVSAQQARERKKVYVNDL
GmbZIP120.1	DKESKRLKR	LLRNRVSAQQARERKKAYLIDL
GmbZIP120.2	DKESKRLKR	LLRNRVSAQQARERKKAYLIDL
MtbZIP20	DKESKRLKR	LLRNRVSAQQARERKKAYLSDL
MtbZIP47	DKEHRRLKR	LLRNRVSAQQARERKKLYVNDL
PvbZIP36.1	DKESKRLKR	LLRNRVSAQQARERKKAYLIDL
PvbZIP36.2	DKESKRLKR	LLRNRVSAQQARERKKAYLIDL
PvbZIP49	TFDDTNR	LLRNRVSAQQARERKKVYVNEL
PvbZIP62	DKEHRRLKR	LLRNRVSAQQARERKKVYVNDL
CabZIP57	DKEYRRHKR	LLRNRVSAQQARERKKVYVNDL
CabZIP59	DKESKRLKR	LLRNRVSAQQARERKKAYLSDL
CcbZIP25	DKESKRLKR	LLRNRVSAQQARERKKAYLIDL
CcbZIP31	DKEHRRLKR	LLRNRVSAQQARERKKVYVNDL
LjbZIP24	DKESKRLKR	LLRNRVSAQQARERKKAYLTDL
LjbZIP25	DKESKRLKR	LLRNRVSAQQARERKKAYLTDL

c

I

GmbZIP8	LMDPKRAKR	ILANRQSAARSKERKIRYTSEL
GmbZIP14	TIDPKRAKR	ILANRQSAARSKERKARYIQEL
GmbZIP27	STDPKRAKR	ILANRQSAARSKERKMRYIAEL
GmbZIP33.1	TADPKRAKR	ILANRQSAARSKERKACYVLQL
GmbZIP33.2	TADPKRAKR	ILANRQSAARSKERKACYVLQL
GmbZIP65	DPKRVKR	ILCNRKSAAKSKERRVIYEKDL
GmbZIP70	LTDPKRAKR	ILANRQSAARSKERKMRYISEL
GmbZIP72	LVDPKRAKR	IWANRQSAARSKERKMRYISEL
GmbZIP76	NIDPKRAKR	ILANRQSAARSKERKARYIQEL
GmbZIP78.1	LIDPKRAKR	ILANRQSAARSKERKMRYISEL
GmbZIP78.2	LIDPKRAKR	ILANRQSAARSKERKMRYISEL
GmbZIP81	LVDPKRAKR	IWANRQSAARSKERKMRYISEL
GmbZIP97	LIDPKRAKR	IWANRQSAARSKERKMRYIAEL
GmbZIP103	TIDPKRAKR	ILANRQSAARSKERKARYIQEL
GmbZIP104.1	MADPKRAKR	ILANRLSAARSKERKMRYISEL
GmbZIP104.2	MADPKRAKR	ILANRLSAARSKERKMRYISEL
GmbZIP105.1	LIDPKRAKR	IWANRQSAARSKERKMRYIAEL
GmbZIP105.2	LIDPKRAKR	IWANRQSAARSKERKMRYIAEL
GmbZIP113	LTDPKRAKR	MLANRQSAARSKERKIRYTSEL
GmbZIP117	MADPKRAKR	ILANRLSAARSKERKMRYISEL
GmbZIP119	NIDPKRAKR	ILANRQSAARSKERKARYIQEL
MtbZIP4.1	AADPKRAKR	ILANRQSAARSKERKMKYISEL
MtbZIP4.2	AADPKRAKR	ILANRQSAARSKERKMKYISEL
MtbZIP18	LIDPKRAKR	IWANRQSAARSKERKMRYIAEL
MtbZIP22	TIDPKRAKR	ILANRQSAARSKERKARYIQEL
MtbZIP24	TVDPKRAKR	ILANRQSAARSKERKACYVVEL
MtbZIP32.1	MADPKRAKR	ILANRQSAARSKERKMRYISEL
MtbZIP32.2	MADPKRAKR	ILANRQSAARSKERKMRYISEL
MtbZIP40	SVDPKRAKR	ILANRQSAARSKERKARYIHEL
MtbZIP44	LIDPKRAKR	ILANRQSAARSKERKTRYTSEL
PvbZIP1.1	MADPKRAKR	ILANRLSAARSKERKMRYISEL
PvbZIP1.2	MADPKRAKR	ILANRLSAARSKERKMRYISEL
PvbZIP1.3	MADPKRAKR	ILANRLSAARSKERKMRYISEL
PvbZIP28	LIDPKRAKR	ILANRQSAARSKERKIRYTSEL
PvbZIP34	LIDPKRAKR	IWANRQSAARSKERKMRYIAEL
PvbZIP37	NIDPKRAKR	ILANRQSAARSKERKARYIQEL
PvbZIP52	TVDPKRAKR	ILANRQSAARSKERKARYIQEL
PvbZIP60.1	TVDPKRAKR	ILANRQSAARSKERKACYVSEL
PvbZIP60.2	TVDPKRAKR	ILANRQSAARSKERKACYVSEL
PvbZIP60.3	TVDPKRAKR	ILANRQSAARSKERKACYVSEL
PvbZIP65	LTDPKRAKR	ILANRQSAARSKERKMRYISEL
PvbZIP68	LIDPKRAKR	IWANRQSAARSKERKMRYISEL
CabZIP1	LIDPKRAKR	IWANRQSAARSKERKMRYIAEL
CabZIP8	SIDPKRAKR	ILANRQSAARSKERKARYIQEL
CabZIP20	MADPKRAKR	ILANRQSAARSKERKMRYISEL
CabZIP24	DPKKLKR	ILDNRKAAARSKERKKRYQDEL
CabZIP29	MSDPKRAKR	ILANRLSAARSKERKMRYISEL
CabZIP31	TIDPKRAKR	ILANRQSAARSKERKARYIQEL
CabZIP36	TVDPKRAKR	ILANRQSAARSKERKACYVVEL
CabZIP55	LIDPKRAKR	ILANRQSAARSKERKTRYTSEL
CcbZIP3	TIDPKRAKR	ILANRQSAARSKERKARYIQEL
CcbZIP14	LSDPKRAKR	ILANRQSAARSKERKMRYISEL
CcbZIP18	LIDPKRAKR	IWANRQSAARSKERKIRYISEL
CcbZIP30	TVDPKRAKR	ILANRQSAARSKERKACYVSEL

c

Group	Protein	Sequence	
I	CcbZIP32	NIDPKRAKR ILANRQSAARSKERKARYIQEL	c
	CcbZIP33	LIDPKRAKR ILANRQSAARSKERKIRYTSEL	
	CcbZIP49	MADPKRAKR ILANRLSAARSKERKMRYISEL	
	CcbZIP58	STDPKRAKR ILANRQSAARSKERKMRYITEL	
	LjbZIP1	TSDPKRAKR ILANRLSAARSKERKTRYISEL	
	LjbZIP13	LIDPKRAKR IWANRQSAARSKERKMRYIAEL	
	LjbZIP28	RVDPVRARR IVANRESAARSKERKNRYVSEM	
	LjbZIP17	ILANRQSAARSKERKMRYISEL	d
	LjbZIP30	MNRILANRQSAARSKERKARYIQEL	
S	GmbZIP2	IIDERKHRRMISNRESARRSRMRKQKHLDEL	d
	GmbZIP5	IMEERKRRRMISNRESARRSRVRKQRHLENL	
	GmbZIP6	VMYERKRKRMESNRESARRSRMKKQKQLEDL	
	GmbZIP10	IIDERKHRRMISNRESARRSRMRKQKHLDEL	
	GmbZIP16	QMDETNMKRRALNREYARQSRLRKHKRLEDL	
	GmbZIP22.1	IIDERKRKRMLSNRESARRSRMRKQKQLEDL	
	GmbZIP22.2	IIDERKRKRMLSNRESARRSRMRKQKQLEDL	
	GmbZIP25	LMEQRKRKRMISNRESARRSRMRKQKHLDDL	
	GmbZIP28	ITDERKNKRKQSNRESARRSRMRKRNHLDQL	
	GmbZIP30	SPEERKLRRMKSNRESARRSRYRKKQHMENL	
	GmbZIP35	LLDDRKKKRMFSNRESARRSRMRKKQQIEVL	
	GmbZIP37	MMDERKRRRMISNRESARRSRMRKQRHLENL	
	GmbZIP39	IMEQRKRKRMLSNRESARRSRIRKQQHLEGL	
	GmbZIP41	MMDQRKRKRMISNRESARRSRMRKQKHLDDL	
	GmbZIP43	VVDERKKKRKQSNRESARRSRMRKRKHLDEL	
	GmbZIP45	SPEERKLRRMQSNRESARRSRYRKKQHIENL	
	GmbZIP52	IMEQRKRKRMLSNRESARRSRMRKQQHLEGL	
	GmbZIP56	LINERKHRRMISNRESARRSRMRKQKHLDEL	
	GmbZIP60	AMDERKRKRMESNRESARRSRMKKQKLLEDL	
	GmbZIP68	VMEERKRRRMISNRESARRSRIRKQRHLENL	
	GmbZIP71	MEDQRKRKRMISNRESARRSRMRKQKHLDDL	
	GmbZIP79	VMDQRKRKRMISNRESARRSRMRKQKHLDDL	
	GmbZIP90	LINERKHRRMLSNRESARRSRMRKQKHLDEL	
	GmbZIP98	LMEQRKKKRKQSNRESARRSRMRKQKHLDDL	
	GmbZIP99	SSHERKLRRMQSNRESARRSRWRKKRHLENL	
	GmbZIP114	AMEERKRRRMISNRESARRSRMRKQRHLENL	
	GmbZIP116	LMEQRKKKRKQSNRESARRSRMRKQKHLDDL	
	GmbZIP121	LINERKHRRMISNRESARRSRMRKQKHLDEL	
	GmbZIP122	LINERKHRRMISNRESARRSRMRKQKHLDEL	
	GmbZIP130	MIDERKRKRMLSNRESARRSRMRKQKQLEDL	
	MtbZIP2	STRERKIRRMQSNRESARRSRWRKKRHVENL	
	MtbZIP3	LMDQRKRKRKQSNRESARRSRMRKQKHMDDL	
	MtbZIP8	QIDERKRKRMLSNRESARRSRLRKQQQVEDL	
	MtbZIP26	SVKERKLRRMQSNRESAQRSRYKKKKHLETV	
	MtbZIP28	MDQRKNKRKQSNRESAKRCRMRKHKHVDDM	
	MtbZIP29	LMDQRKRKRMISNRESARRSRMRKQKHLDDL	
	MtbZIP31	LMDQRKRKRMISNRESARRSRMRKQKHLDDL	
	MtbZIP36	VVDERKRRRMISNRESARRSRMRKQRHVENL	
	MtbZIP37	NMEDRKRRRMISNRESARRSRMRKQRHLENL	
	MtbZIP42	LINERKHRRMISNRESARRSRMRKQKHLDEL	
	MtbZIP45	LINERKHRRMVSNRESARRSRMRKQKQLDEL	
	MtbZIP46	IIDERKHRRMISNRESARRSRMRKQKHLDEL	
	MtbZIP54	AMDERKRKRMISNRESARRSRERKQKLLEDY	
	MtbZIP58	IIDERKHRRMISNRESARRSRMRKQKHLDEL	
U	GmbZIP21	EKEVRRIRRILANRESARQTIRRRQ ALCEEL	a
	GmbZIP128	EKEARRIRRILANRESARQTIRRRQ ALCEEL	
	MtbZIP52	EKEARRIRRVLANRESARQTIRRRQ ALSEEL	
	PvbZIP9.1	EKEARRIRRVLANRESARQTIRRRQ ALCEEL	
	PvbZIP9.2	EKEARRIRRVLANRESARQTIRRRQ ALCEEL	
	CabZIP15	EKEARRIRRVMANRESARQTIRRRQ ALSEEL	
	MtbZIP11	SDEHK TLRRLMQNREAARKSRLRKK AYVQQL	b
	GmbZIP12	EPMSKKLKR QLRNRDAAVRSRERKKLYVKNL	c
	GmbZIP75	DADLKKLRR MESNRLSSRRSWMKKLIYLTNL	
	GmbZIP102	QEMERKFRR TISNRFSARRSRLKKLAYMAEL	
	MtbZIP6.1	EPVSKKQIR QMRNRDAAVKSRERKKVYVKNL	
	MtbZIP6.2	EPVSKKQIR QMRNRDAAVKSRERKKVYVKNL	
	PvbZIP46	EPTSKKLKR KLRNRDAAVKSRERKKVYVKNL	
	CabZIP58	EPVSKKEIR QIRNRDAAVRSRERKKMYVKNL	
	CcbZIP57	EPMSKKLKR QLRNRDAAVRSRERKKLYVKDL	
