# Supplementary figures and images for: Genome-wide analysis of the basic leucine zipper (bZIP) transcription factor gene family in six legume genomes
Source: BMC Genomics. 2015 Dec 10;16:1053. doi: 10.1186/s12864-015-2258-x (PMC4676100; doi:10.1186/s12864-015-2258-x)

[illegible]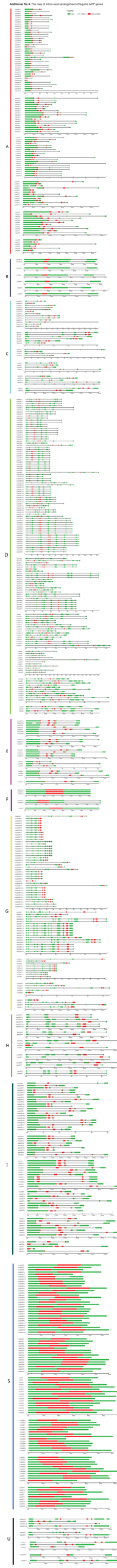

Supplement: Additional file 4: — The map of intron-exon arrangement of legume bZIP genes. (PDF 1488 kb) [file 12864_2015_2258_MOESM4_ESM.pdf]

**Additional file 5.** Histogram of intron number of legume bZIP genes in each group.

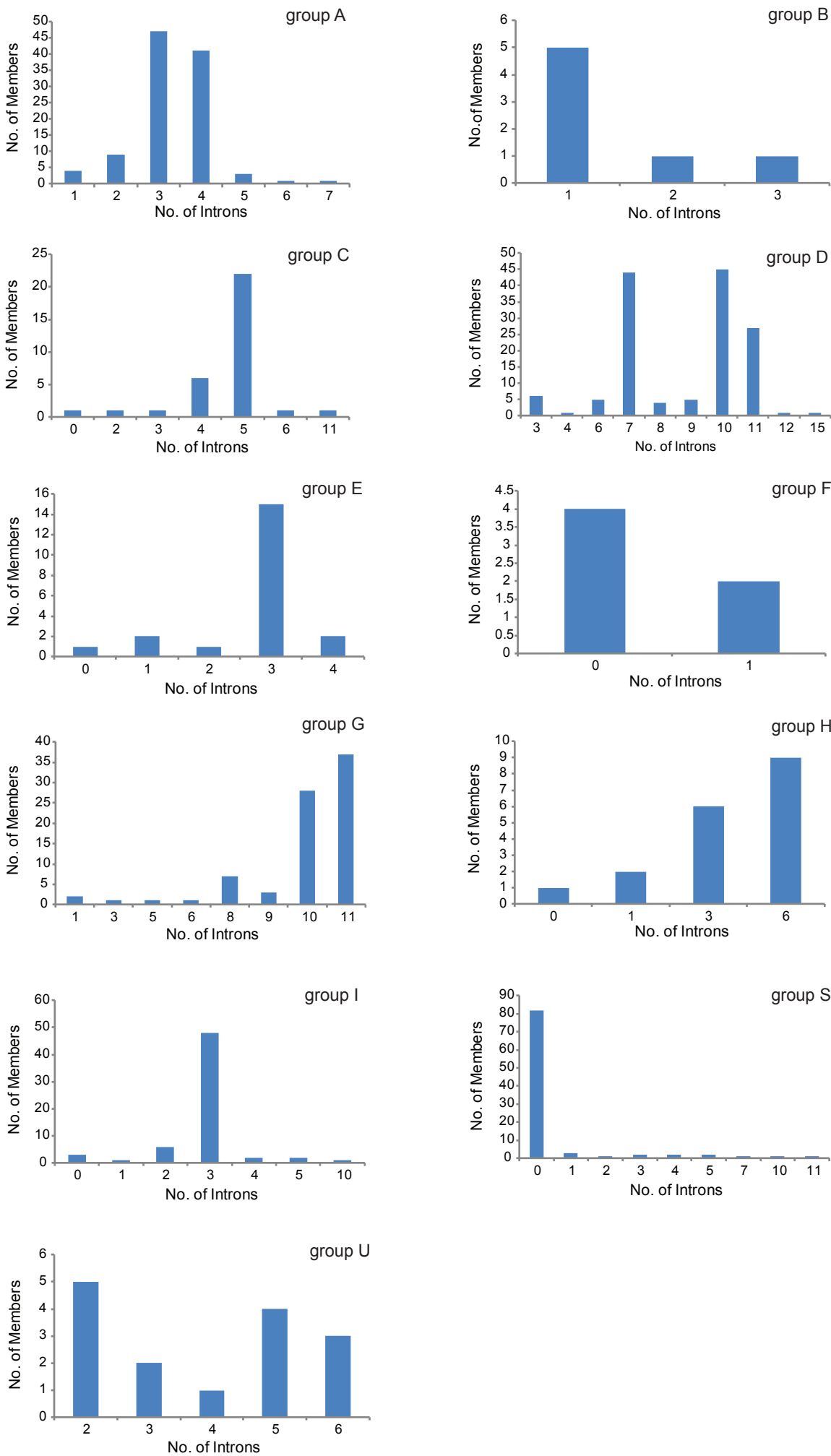

Supplement: Additional file 5: — Histogram of intron number of legume bZIP genes in each group. (PDF 410 kb) [file 12864_2015_2258_MOESM5_ESM.pdf]
