# Supplementary material for: Genome-wide analysis of the basic leucine zipper (bZIP) transcription factor gene family in six legume genomes
Source: BMC Genomics. 2015 Dec 10;16:1053. doi: 10.1186/s12864-015-2258-x (PMC4676100; doi:10.1186/s12864-015-2258-x)
Supplement: Additional file 7: — Alignment of basic and hinge regions of 585 legume bZIP proteins. (PDF 1687 kb) [file 12864_2015_2258_MOESM7_ESM.pdf]

Additional file 7. Alignment of basic and hinge regions of 585 legume bZIP proteins. The legume bZIP proteins are classified into 11 groups (A-I, S, U). Conserved amino acids are boxed in red. Some of the functional annotated bZIP proteins in maize, rice and Arabidopsis sharing similar amino acid sequences in the basic and hinge regions are also shown as reference. The different amino acid residues at -10 and -18 positions like K and I are colored.

GROUP	bZIP NO.	PUBLISHED	BASIC REGION / HINGE
			-40 -25 -20 -15 -10 -5 -1
	AtbZIP12	DPB4	--VERRQKRMIKNRESAARSRARKQAYTHEL
	OsbZIP66	TRAB1	--VERRQRRMIKNRESAARSRARKQAYTMEL
	ZmbZIP23	ZmbZIP72	--VERRRRRMIKNRESAARSRARKQAYTMEL
	GmbZIP11		KVVERRQRRMIKNRESAARSRARKQAYTFEL
	GmbZIP15		KTVERRQKRMIKNRESAARSRARKQAYTQEL
	GmbZIP26		TLLDQRHARIIKNRESAVRSRARKQAYRKGL
	GmbZIP29		KVIERRQRRMIKNRESAARSRARKQAYTMEL
	GmbZIP32		KTVERRQKRMIKNRESAARSRARKQAYTTEL
	GmbZIP36		KTVERRQKRMIKNRESAARSRARRQAYTQEL
	GmbZIP38.1		KATLQKLRRMIKNRESAARSRERKQAYTSEL
	GmbZIP38.2		KATLQKLRRMIKNRESAARSRERKQAYTSEL
	GmbZIP38.3		KATLQKLRRMIKNRESAARSRERKQAYTSEL
	GmbZIP38.4		KATLQKLRRMIKNRESAARSRERKQAYTSEL
	GmbZIP42		TLQDQRHTRVIKNRESAVRSRARKQAYRKGL
	GmbZIP44		KVIERRQRRMIKNRESAARSRARKQAYTMEL
	GmbZIP48.1		KTVERRQKRMIKNRESAARSRARKQAYTTEL
	GmbZIP48.2		KTVERRQKRMIKNRESAARSRARKQAYTTEL
	GmbZIP50.1		KVVERRQRRMIKNRESAARSRARKQAYTFEL
	GmbZIP50.2		KVVERRQRRMIKNRESAARSRARKQAYTFEL
	GmbZIP50.3		KVVERRQRRMIKNRESAARSRARKQAYTFEL
	GmbZIP51		KATLQKQRRMIKNRESAARSRERKQAYTSEL
	GmbZIP54		KVTLQKQRRMIKNRESAARSRERKQAYTVEL
	GmbZIP55.1		KTVERRQKRMIKNRESAARSRARKQAYTNEL
	GmbZIP55.2		KTVERRQKRMIKNRESAARSRARKQAYTNEL
	GmbZIP61		KVVERRQRRMIKNRESAARSRARKQAYTVEL
	GmbZIP64		KAAQQRQRRMIKNRESAARSRERKQAYQVEL
	GmbZIP84		VVVERRQRRMLKNRESAARSRARRQAYTVEL
	GmbZIP87		KTIERKQKRMAKNRESVVRSRTKKQEHINKL
	GmbZIP89.1		KTLERRLRRKIKNRESAARSRARKQAYHNEL
	GmbZIP89.2		KTLERRLRRKIKNRESAARSRARKQAYHNEL
	GmbZIP89.3		KTLERRLRRKIKNRESAARSRARKQAYHNEL
	GmbZIP89.4		KTLERRLRRKIKNRESAARSRARKQAYHNEL
	GmbZIP91.1		KVVERRQRRMIKNRESAARSRARKQAYTVEL
	GmbZIP91.2		KVVERRQRRMIKNRESAARSRARKQAYTVEL
	GmbZIP96		VVVERRQRRMLKNRESAARSRARRQAYTVEL
	GmbZIP101		RTIERRQKRMAKNRESAGRSRAKKQEHINRL
	GmbZIP106.1		KVTLQKQRRMIKNRESAARSRERKQAYTVEL
	GmbZIP106.2		KVTLQKQRRMIKNRESAARSRERKQAYTVEL
	GmbZIP107		KTVERRQKRMIKNRESAARSRARKQAYTNEL
	GmbZIP123		KTVERRQKRMIKNRESAARSRARRQAYTQEL
	GmbZIP124		KIVERRQKRMIKNRESAARSRARKQAYTQEL
	GmbZIP129		KVVERRQRRMIKNRESAARSRARKQAYTVEL
	GmbZIP135		KTLERRLRRKIKNRESAARSRARKQAYHNEL
	MtbZIP9.1		KAAQQRQRRMIKNRESAARSRERKQAYQVEL
	MtbZIP9.2		KAAQQRQRRMIKNRESAARSRERKQAYQVEL
	MtbZIP9.3		KAAQQRQRRMIKNRESAARSRERKQAYQVEL
	MtbZIP14		VVVERRQRRMLKNRESAARSRARRQAYTVEL
	MtbZIP15.1		VLLDRKQRRMMKNRESAARSRARKQAYTIEL
A	MtbZIP15.2		VLLDRKQRRMMKNRESAARSRARKQAYTIEL
	MtbZIP19		RTVERKQKRMIKNRESAARSRARKQAYTTEL
	MtbZIP25.1		KVIERRQRRMIKNRESAARSRARKQAYTMEL
	MtbZIP25.2		KVIERRQRRMIKNRESAARSRARKQAYTMEL
	MtbZIP34		KGVERRQKRMIKNRESAARSRARKQAYTVEL
	MtbZIP35		KAALQKQKRMIKNRESAARSRERKQAYTTEL
	MtbZIP41.1		KALERRLRRKIKNRESAARSRARKQAYHNEL
	MtbZIP41.2		KALERRLRRKIKNRESAARSRARKQAYHNEL
	MtbZIP43		KTVERRQKRMIKNRESAARSRARRQAYTQEL
	MtbZIP48		KTVERRQKRMIKNRESAARSRARKQAYTQEL
	MtbZIP53		RVVERRQRRMIKNRESAARSRARKQAYTVEL
	MtbZIP61		KTVERRQKRMIKNRESAARSRARKQAYTNEL
	PvbZIP5		KTVERRQKRMIKNRESAARSRARKQAYTHEL
	PvbZIP10		KVVERRQRRMIKNRESAARSRARKQAYTVEL
	PvbZIP16		KATLQKQRRMIKNRESAARSRERKQAYTSEL
	PvbZIP22		SRDMRNARLMKNRESAARSRARKQAYLIELK
	PvbZIP24.1		KVVERRQRRMIKNRESAARSRARKQAYTFEL
	PvbZIP24.2		KVVERRQRRMIKNRESAARSRARKQAYTFEL
	PvbZIP26		KATIQKQRRMIKNRESAARSRERKQAYTMEL
	PvbZIP27		RTVERRHKRMIKNRESAARSRARRQAYTQEL
	PvbZIP29		KTFDRRQKRMIKNRESAARSRARKQAYTIEL
	PvbZIP33		EVIERRQRRMLKNRESAARSRARRQAYTVEL
	PvbZIP35		KTLERRLRRKIKNRESAARSRARKQAYHNEL
	PvbZIP42		KAAQQRQRRMIKNRESAARSRERKQAYQVEL
	PvbZIP45		KVVERRQRRMIKNRESAARSRARKLAYTVEL
	PvbZIP51		KAVERRQKRMAKNRESAARSRAKKQEHIHRL
	PvbZIP54		HHQDQRHVRIMKNRESAVRSRARKQAYRRGL
	PvbZIP57.1		KVIERRQRRMIKNRESAARSRARKQAYTMEL
	PvbZIP57.2		KVIERRQRRMIKNRESAARSRARKQAYTMEL
	PvbZIP71.1		KTVERRQKRMIKNRESAARSRARKQAYTNEL
	PvbZIP71.2		KTVERRQKRMIKNRESAARSRARKQAYTNEL
	PvbZIP71.3		KTVERRQKRMIKNRESAARSRARKQAYTNEL
	CabZIP4		VVAERRQRRMLKNRESAARSRARRQAYTVEL
	CabZIP6		KTVERRQKRMIKNRESAARSRARRQAYTQEL
	CabZIP9		KALERRLRRKIKNRESAARSRARKQAYHNEL
	CabZIP11		KTVERRQKRMIKNRESAARSRARKQAYTQEL
	CabZIP16		RVVERRQRRMIKNRESAARSRARKQAYTVEL
	CabZIP23		KAAQQRQRRMIKNRESAARSRERKQAYQVEL
	CabZIP30		KTVERKQKRMIKNRESAARSRARKQAYTTEL
	CabZIP34		KVIERRQRRMIKNRESAARSRARKQAYTMEL
	CabZIP39		KAALARQKRLIKNRESAAKSRAKKERYVSGL
	CabZIP40		KVVERRQRRMIKNRESAARSRARKQAYTVEL
	CabZIP52		KTVERRQKRMIKNRESAARSRARKQAYTNEL
	CcbZIP10		KVVERRQRRMIKNRESAARSRARKQAYTVEL
	CcbZIP20		KTVERRQKRMIKNRESAARSRARKQAYTQEL
	CcbZIP24		KVIERRQRRMIKNRESAARSRARKQLKNSVS
	CcbZIP26		RTVERRQKRMIKNRESAARSRARKQAYTTEL
	CcbZIP35		KAAQQRQRRMIKNRESAARSRERKQAYQVEL
	CcbZIP37		KATLQKQRRMIKNRESAARSRERKQAYTVEL
	CcbZIP50		KTLERRLRRKIKNRESAARSRARKQAYHNEL
	CcbZIP52		THQDQRHMRILRNRESAVRSRARKQAYRKGL
	CcbZIP53		KATLQKQRRMIKNRESAARSRERKQAYTSEL
	CcbZIP56		KTVERRQKRMIKNRESAARSRARKQAYTQEL
	CcbZIP59		KVVERRQRRMIKNRESAARSRARKQAYTFEL
	CcbZIP61		KVVERRQRRMIKNRESAARSRARKHAYTVEL
	LjbZIP11		GLGDRRNKRMIKNRESAARSRARKQAYTNEL
	LjbZIP14		KTVERRQKRMIKNRESAARSRARKQAYTNEL
	LjbZIP18		KATLQKQRRMIKNRESAARSRERKQAYTTEL
	LjbZIP23		KAAQQRQRRMIKNRESAARSRERKQAYQVEL
	LjbZIP26		KATLQKQRRMIKNRESAARSRERKQAYTLEL
	LjbZIP29		KTVERRQKRMIKNRESAARSRARKQAYTTEL

B

AtbZIP17		--DEKKRARLMRNRESAQLSRQRKKHYVEEL
OsbZIP50		--MSKKKRRQMRNRDSAMKSRERKKMYVKDL
ZmbZIP28		--EAKRRARQVRNRESAHLSRQRKKQYVEEL
GmbZIP18		DEDEKRKARLMRNRESAQLSRQRKKHYVEEL
GmbZIP125		DEDEKRKARLMRNRESAQLSRQRKKHYVEEL
MtbZIP49		DEDEKRKARLMRNRESAQLSRQRKKHYVEEL
PvbZIP6		DDDEKRKARLMRNRESAQLSRQRKKHYVEEL
CabZIP12		DEDEKRKARLMRNRESAQLSRQRKKHYVEEL
CcbZIP21		DDDEKRKARLMRNRESAQLSRQRKKHYVEEL
LjbZIP5		DDDEKRKARLMRNRESAQLSRQRKKHYVEEL

C

AtbZIP9	BZO2H2	--DLKRIRRMNSNRESAKRSRRRKQEYLVDL
OsbZIP33	REB	--DQRLQRRKQSNRESARRSRSRKAAHLNEL
ZmbZIP17	OHP1	--QQRLQRRKQSNRESARRSRSRKAAHLNEL
GmbZIP23		PVDAKRVRRMLSNRESARRSRRRKQAHLTEL
GmbZIP31.1		PADMKRLRRKVSNRDSARRSRRRKQAQLSEL
GmbZIP31.2		PADMKRLRRKVSNRDSARRSRRRKQAQLSEL
GmbZIP31.3		PADMKRLRRKVSNRDSARRSRRRKQAQLSEL
GmbZIP46.1		PADMKRLRRKVSNRDSARRSRRRKQAQLSDL
GmbZIP46.2		PADMKRLRRKVSNRDSARRSRRRKQAQLSDL
GmbZIP63		PADVKRVRRMLSNRESARRSRRRKQAHLTDL
GmbZIP115.1		AIDVKRLRRKVSNRESARRSRRRKQAHLADL
GmbZIP115.2		AIDVKRLRRKVSNRESARRSRRRKQAHLADL
GmbZIP131		PVDAKRVRRMLSNRESARRSRRRKQAHLTEL
GmbZIP134		PVDAKRVRRMLSNRESDRCSRRRKQTHLTEL
GmbZIP137.1		PADVKRVRRMLSNRESARRSRRRKQAHLTDL
GmbZIP137.2		PADVKRVRRMLSNRESARRSRRRKQAHLTDL
MtbZIP1.1		PVDMKRLRRKVSNRESARRSRRRKQAHLADL
MtbZIP1.2		PVDMKRLRRKVSNRESARRSRRRKQAHLADL
MtbZIP7		PTDVKRVRRMLSNRESARRSRRRKQAHLTEL
MtbZIP27		PVDMKRQRRKDSNCESARRSRWRKQAHLSEL
MtbZIP55		PTDAKRVRRMLSNRESARRSRRRKQAHLTEL
MtbZIP56		PSDAKRLKRMLQNRESAKRSRDRKIAKNTEL
PvbZIP4		EKAQVKMRTKNSNRESARRSRRRKQAHLADL
PvbZIP38		PTDAKRVRRMLSNRESARRSRRRKQAHLSDL
PvbZIP43		PADAKRVRRMLSNRESARRSRRRKQAHLTDL
PvbZIP59		AVELKRLRRKVSNRNSARRSRRRKQAQLADL
CabZIP18		RADAKRVRRMLSNRESARRSRRRKQAHLTDL
CabZIP21		PADVKRVRRMLSNRESARRSRRRKQAHLTEL
CabZIP26		PIDMKRLRRKVSNRESARRSRRRKQAHLTDL
CabZIP33		PLHMKRLRRKVSNRDSARRSRQRKQAHLAEL
CcbZIP5		PADAKRVRRMLSNRESARRSRRRKQAHLTDL
CcbZIP7		PLEVKRLKRMDSNRESARRSRRRKQAHLADL
CcbZIP11		PLDAKRVRRMLSNRESARRSRRRKQAHLTEL
CcbZIP55		AIDIKRLRRKFSNRESARRSRRRKQAHLADL
LjbZIP3		PQDVKRLRRKVSNRESARRSRRRKQAHLAEL
LjbZIP21		PSDAKRVRRMLSNRESARRSRRRKQAHLTDL

D

AtbZIP20	AHBP-1b/TGA2	--DQKTLRRLAQNREAARKSRLRKKAYVQQL
OsbZIP38.1	LG2	--DAKTERRLAQNREAARKSRLRKKAYVQQL
ZmbZIP4		--DQKVLRRLAQNREAARKSRLRKKAYVQQL
GmbZIP3.1		PGDQKTLRRLAQNREAARKSRLRKKAYVQQL
GmbZIP3.2		DDIIPTLRRLAQNREAARKSRLRKKAYVQQL
GmbZIP9.1		PGDQKTLRRLAQNREAARKSRLRKKAYVQQL
GmbZIP9.2		KDATKTLRRLAQNREAARKSRLRKKAYVQQL
GmbZIP13.1		TPDPKTLRRLAQNREAARKSRLRKKAYVQQL
GmbZIP13.2		TPDPKTLRRLAQNREAARKSRLRKKAYVQQL
GmbZIP19.1		KSDQKTLRRLAQNREAARKSRLRKKAYVQQL
GmbZIP19.2		KSDQKTLRRLAQNREAARKSRLRKKAYVQQL
GmbZIP20		TPDPKTLRRLAQNREAARKSRLRKKAYVQQL
GmbZIP34.1		NVENKMLRRLAQNREAARKSRLRKKAYVKQL
GmbZIP34.2		NVENKMLRRLAQNREAARKSRLRKKAYVKQL
GmbZIP40.1		RLTDKTQRRLAQNREAARKSRLRKKAYVQQL
GmbZIP40.2		RLTDKTQRRLAQNREAARKSRLRKKAYVQQL
GmbZIP40.3		RLTDKTQRRLAQNREAARKSRLRKKAYVQQL
GmbZIP40.4		RLTDKTQRRLAQNREAARKSRLRKKAYVQQL
GmbZIP53.1		RLTDKTQRRLAQNREAARKSRLRKKAYVQQL
GmbZIP53.2		RLTDKTQRRLAQNREAARKSRLRKKAYVQQL
GmbZIP53.3		RLTDKTQRRLAQNREAARKSRLRKKAYVQQL
GmbZIP53.4		RLTDKTQRRLAQNREAARKSRLRKKAYVQQL
GmbZIP53.5		RLTDKTQRRLAQNREAARKSRLRKKAYVQQL
GmbZIP53.6		RLTDKTQRRLAQNREAARKSRLRKKAYVQQL
GmbZIP62		TPDPKTLRRLAQNREAARKSRLRKKAYVQQL
GmbZIP66		AEDQKTVRRLAQNREAARKSRLRKKAYVQQL
GmbZIP67.1		KSDQKSLRRLAQNREAARKSRLRKKAYVQQL
GmbZIP67.2		KSDQKSLRRLAQNREAARKSRLRKKAYVQQL
GmbZIP67.3		KSDQKSLRRLAQNREAARKSRLRKKAYVQQL
GmbZIP74.1		PLDAKTLRRLAQNREAARKSRLRKKAYVQQL
GmbZIP74.2		PLDAKTLRRLAQNREAARKSRLRKKAYVQQL
GmbZIP77.1		KPTDKIQRRLAQNREAARKSRLRKKAYVQQL
GmbZIP77.2		KPTDKIQRRLAQNREAARKSRLRKKAYVQQL
GmbZIP77.3		KPTDKIQRRLAQNREAARKSRLRKKAYVQQL
GmbZIP77.4		KPTDKIQRRLAQNREAARKSRLRKKAYVQQL
GmbZIP77.5		KPTDKIQRRLAQNREAARKSRLRKKAYVQQL
GmbZIP82.1		PLDAKTLRRLAQNREAARKSRLRKKAYVQQL
GmbZIP82.2		PLDAKTLRRLAQNREAARKSRLRKKAYVQQL
GmbZIP82.3		PLDAKTLRRLAQNREAARKSRLRKKAYVQQL
GmbZIP82.4		PLDAKTLRRLAQNREAARKSRLRKKAYVQQL
GmbZIP82.5		PLDAKTLRRLAQNREAARKSRLRKKAYVQQL
GmbZIP82.6		PLDAKTLRRLAQNREAARKSRLRKKAYVQQL
GmbZIP85.1		PLDAKTLRRLAQNREAARKSRLRKKAYVQQL
GmbZIP85.2		PLDAKTLRRLAQNREAARKSRLRKKAYVQQL
GmbZIP85.3		PLDAKTLRRLAQNREAARKSRLRKKAYVQQL
GmbZIP85.4		PLDAKTLRRLAQNREAARKSRLRKKAYVQQL
GmbZIP85.5		PLDAKTLRRLAQNREAARKSRLRKKAYVQQL
GmbZIP85.6		PLDAKTLRRLAQNREAARKSRLRKKAYVQQL
GmbZIP88.1		KADTKALRRQAQNREAARKCRLRKKAYVQQL
GmbZIP88.2		KADTKALRRQAQNREAARKCRLRKKAYVQQL
GmbZIP88.3		KADTKALRRQAQNREAARKCRLRKKAYVQQL
GmbZIP88.4		KADTKALRRQAQNREAARKCRLRKKAYVQQL
GmbZIP88.5		KADTKALRRQAQNREAARKCRLRKKAYVQQL
GmbZIP92		KSDQKTLRRLAQNREAARKSRLRKKAYVQQL
GmbZIP95.1		PLDAKALRRLAQNREAARKSRLRKKAYVQQL
GmbZIP95.2		PLDAKALRRLAQNREAARKSRLRKKAYVQQL
GmbZIP95.3		PLDAKALRRLAQNREAARKSRLRKKAYVQQL
GmbZIP100.1		KADTKALRRQAQNREAARKCRLRKKAYVQQL
GmbZIP100.2		KADTKALRRQAQNREAARKCRLRKKAYVQQL
GmbZIP100.3		KADTKALRRQAQNREAARKCRLRKKAYVQQL
GmbZIP100.4		KADTKALRRQAQNREAARKCRLRKKAYVQQL
GmbZIP108.1		KSDQKTLRRLAQNREAARKSRLRKKAYVQQL
GmbZIP108.2		KSDQKTLRRLAQNREAARKSRLRKKAYVQQL
GmbZIP118.1		KPTDKIQRRLAQNREAARKSRLRKKAYVQQL
GmbZIP118.2		KPTDKIQRRLAQNREAARKSRLRKKAYVQQL
GmbZIP118.3		KPTDKIQRRLAQNREAARKSRLRKKAYVQQL
GmbZIP118.4		KPTDKIQRRLAQNREAARKSRLRKKAYVQQL
GmbZIP118.5		KPTDKIQRRLAQNREAARKSRLRKKAYVQQL
GmbZIP126.1		KSDQKTLRRLAQNREAARKSRLRKKAYVQQL
GmbZIP126.2		KSDQKTLRRLAQNREAARKSRLRKKAYVQQL
GmbZIP126.3		KSDQKTLRRLAQNREAARKSRLRKKAYVQQL
GmbZIP127		TPDPKTLRRLAQNREAARKSRLRKKAYVQQL
GmbZIP136.1		AEDQKTVRRLAQNREAAKKSRLRKKAYVQQL
GmbZIP136.2		AEDQKTVRRLAQNREAAKKSRLRKKAYVQQL
GmbZIP138.1		KSDQKTLRRLAQNREAARKSRLRKKAYVQQL
GmbZIP138.2		KSDQKTLRRLAQNREAARKSRLRKKAYVQQL
GmbZIP138.3		KSDQKTLRRLAQNREAARKSRLRKKAYVQQL
MtbZIP10		-DEHKTLRRLMQNREAARKSRLRKKAYVQQL
MtbZIP16.1		PLDAKTLRRLAQNREAARKSRLRKKAYVQQL
MtbZIP16.2		PLDAKTLRRLAQNREAARKSRLRKKAYVQQL
MtbZIP21		SDEHKTLRRLMQNREAARKSRLRKKAYVQQL
MtbZIP23		RPIDKIQRRLAQNREAARKSRLRKKAYVQQL
MtbZIP30.1		TLDPKTLRRLAQNREAAKKSRLRKKAYVQQL
MtbZIP30.2		TLDPKTLRRLAQNREAAKKSRLRKKAYVQQL
MtbZIP30.3		TLDPKTLRRLAQNREAAKKSRLRKKAYVQQL
MtbZIP39.1		AEDHKTLRRLAQNREAARKSRLRKKAYVQQL
MtbZIP39.2		AEDHKTLRRLAQNREAARKSRLRKKAYVQQL
MtbZIP39.3		AEDHKTLRRLAQNREAARKSRLRKKAYVQQL
MtbZIP39.4		AEDHKTLRRLAQNREAARKSRLRKKAYVQQL
MtbZIP50.1		KSDQKTLRRLAQNREAARKSRLRKKAYVQQL
MtbZIP50.2		KSDQKTLRRLAQNREAARKSRLRKKAYVQQL
MtbZIP50.3		KSDQKTLRRLAQNREAARKSRLRKKAYVQQL
MtbZIP51.1		TPDPKILRRLAQNREAARKSRLRKKAYIQQL
MtbZIP51.2		TPDPKILRRLAQNREAARKSRLRKKAYIQQL
MtbZIP51.3		TPDPKILRRLAQNREAARKSRLRKKAYIQQL
MtbZIP60.1		KSDQKTLRRLAQNREAARKSRLRKKAYVQQL
MtbZIP60.2		KSDQKTLRRLAQNREAARKSRLRKKAYVQQL
MtbZIP60.3		KSDQKTLRRLAQNREAARKSRLRKKAYVQQL
MtbZIP60.4		KSDQKTLRRLAQNREAARKSRLRKKAYVQQL
MtbZIP60.5		KSDQKTLRRLAQNREAARKSRLRKKAYVQQL
MtbZIP65		TPDPKTLRRLAQNREAARKSRLRKKAYVQQL
PvbZIP7		KSDQKTLRRLAQNREAARKSRLRKKAYVQQL
PvbZIP8.1		TPDPKTLRRLAQNREAARKSRLRKKAYVQQL
PvbZIP8.2		TPDPKTLRRLAQNREAARKSRLRKKAYVQQL
PvbZIP12.1		KPTDKIQRRLAQNREAARKSRLRKKAYVQQL
PvbZIP12.2		KPTDKIQRRLAQNREAARKSRLRKKAYVQQL
PvbZIP18		PEDQKTLRRLAQNREAAKKSRLRKKAYVQQL
PvbZIP32		PLDAKTLRRLAQNREAARKSRLRKKAYVQQL
PvbZIP40		KSDQKTLRRLAQNREAARKSRLRKKAYVQQL
PvbZIP41		PEDQKTVRRLAQNREAARKSRLRKKAYVQQL
PvbZIP47.1		TPDPKTLRRLAQNREAARKSRLRKKAYVQQL
PvbZIP47.2		TPDPKTLRRLAQNREAARKSRLRKKAYVQQL
PvbZIP50		KADSKVLRRQAQNREAARKCRLRKKAYVQQL
PvbZIP55		KDVNKMRRRLAQNREAARKSRLRKKAYVKQL
PvbZIP69.1		PLDAKTLRRLAQNREAARKSRLRKKAYVQQL
PvbZIP69.2		PLDAKTLRRLAQNREAARKSRLRKKAYVQQL
PvbZIP72		KSDQKTLRRLAQNREAARKSRLRKKAYVQQL
CabZIP3		PLDAKTLRRLAQNREAARKSRLRKKAYVQQL
CabZIP13		KSDQKTLRRLAQNREAARKSRLRKKAYVQQL
CabZIP14		TPDAKTLRRLAQNREAARKSRLRKKAYIQQL
CabZIP25		AEEHKSLRRLAQNREAARKSRLRKKAYVQQL
CabZIP27		KSDQKTLRRLAQNREAARKSRLRKKAYVQQL
CabZIP35		RPIDKIQRRLAQNREAARKSRLRKKAYVQQL
CabZIP37		KLTDKMQRRLAQNREAARKSRLKKKAYVQQL
CabZIP43		TLDPKTLRRLAQNREAAKKSRLRKKAYVQQL
CabZIP51		KSDQKTLRRLAQNREAARKSRLRKKAYVQQL
CabZIP54		KDDSKVLRRLAQNREAARKSRLRKKAYVQQL
CabZIP56		AEDHKTLRRLAQNREAARKSRLRKKAYVQQL
CcbZIP1		KPTDKIQRRLAQNREAARKSRLRKKAYVQQL
CcbZIP4		TPDPKTLRRLAQNREAARKSRLRKKAYVQQL
CcbZIP6		AEDQKTIRRLAQNREAARKSRLRKKAYVQQL
CcbZIP8		KSDQKTLRRLAQNREAARKSRLRKKAYVQQL
CcbZIP9		TPDPKTLRRLAQNREAARKSRLRKKAYVQQL
CcbZIP15		QLDAKALRRLAQNREAARKSRLRKKAYVQQL
CcbZIP22		KSDQKTLRRLAQNREAARKSRLRKKAYVQQL
CcbZIP23		KSDQKTLRRLAQNREAARKSRLKKKAYVQQL
CcbZIP38		KADRKAQRRLAQNREAARKCRLRKKAYVQQL
CcbZIP48		PLDAKTLRRLAQNREAARKSRLRKKAYVQQL
CcbZIP54		VEDQKTLRRLAQNREAARKSRLRKKAYVQQL
LjbZIP2		RPIDKIQRRLAQNREAARKSRLRKKAYVQQL
LjbZIP6		KTDQKTLRRLAQNREAARKSRLRKKAYVQQL
LjbZIP8		PEDQKILRRLAQNREAAKKSRLRKKAYVQQL

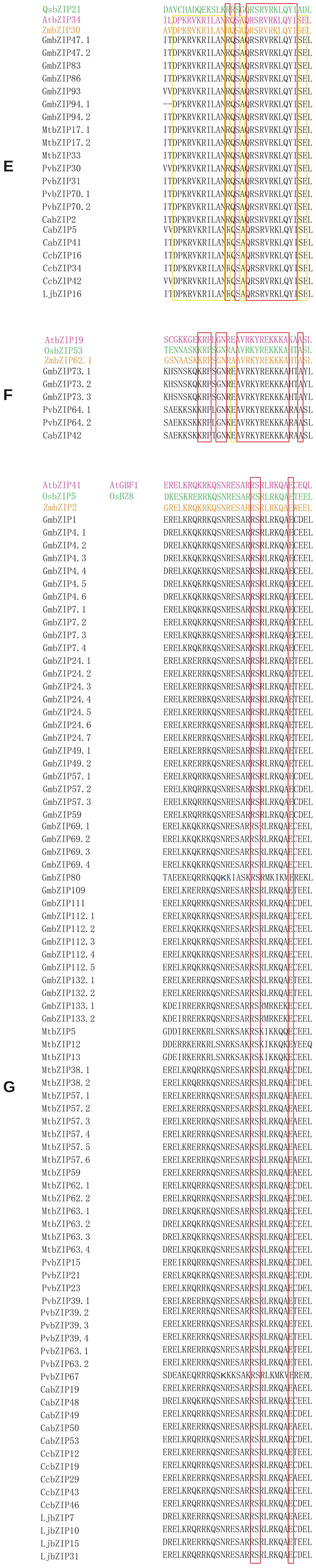

E
QsbZIP21 DAVCHADQEKSLKKRSGQRSRVRKLQYIADL
AtbZIP34 ILDPKRVKRILANRQSAQRSRVRKLQYISEL
ZmbZIP30 AVDPKRVKRILANRQSAQRSRVRKLQYISEL
GmbZIP47.1 ITDPKRVKRILANRQSAQRSRVRKLQYISEL
GmbZIP47.2 ITDPKRVKRILANRQSAQRSRVRKLQYISEL
GmbZIP83 ITDPKRVKRILANRQSAQRSRVRKLQYISEL
GmbZIP86 ITDPKRVKRILANRQSAQRSRVRKLQYISEL
GmbZIP93 VVDPKRVKRILANRQSAQRSRVRKLQYISEL
GmbZIP94.1 --DPKRVKRILANRQSAQRSRVRKLQYISEL
GmbZIP94.2 ITDPKRVKRILANRQSAQRSRVRKLQYISEL
MtbZIP17.1 ITDPKRVKRILANRQSAQRSRVRKLQYISEL
MtbZIP17.2 ITDPKRVKRILANRQSAQRSRVRKLQYISEL
MtbZIP33 ITDPKRVKRILANRQSAQRSRVRKLQYISEL
PvbZIP30 VVDPKRVKRILANRQSAQRSRVRKLQYISEL
PvbZIP31 ITDPKRVKRILANRQSAQRSRVRKLQYISEL
PvbZIP70.1 ITDPKRVKRILANRQSAQRSRVRKLQYISEL
PvbZIP70.2 ITDPKRVKRILANRQSAQRSRVRKLQYISEL
CabZIP2 ITDPKRVKRILANRQSAQRSRVRKLQYISEL
CabZIP5 VVDPKRVKRILANKQSAQRSRVRKLQYISEL
CabZIP41 ITDPKRVKRILANKQSAQRSRVRKLQYISEL
CcbZIP16 ITDPKRVKRILANKQSAQRSRVRKLQYISEL
CcbZIP34 ITDPKRVKRILANKQSAQRSRVRKLQYISEL
CcbZIP42 VVDPKRVKRILANKQSAQRSRVRKLQYISEL
LjbZIP16 ITDPKRVKRILANKQSAQRSRVRKLQYISEL
F
AtbZIP19 SCGKKGEKRPLGNREAVRKYREKKKAKAASL
OsbZIP53 TENNASKKRPSGNRAAVRKYREKKKAHTASL
ZmbZIP62.1 GSNAASKKRPSGNRAAVRKYREKKKAHTASL
GmbZIP73.1 KHSNSKQKRPSGNREAVRKYREKKKAHTAYL
GmbZIP73.2 KHSNSKQKRPSGNREAVRKYREKKKAHTAYL
GmbZIP73.3 KHSNSKQKRPSGNREAVRKYREKKKAHTAYL
PvbZIP64.1 SAEKKSKKRPLGNKEAVRKYREKKKARAASL
PvbZIP64.2 SAEKKSKKRPLGNKEAVRKYREKKKARAASL
CabZIP42 SAEKKSKKRPTGNKEAVRKYREKKKARAASL
G
AtbZIP41 AtGBF1 ERELKRQKRKQSNRESARRSRLRKQAECEQL
OsbZIP5 OsBZ8 DKESKRERRKQSNRESARRSRLRKQAETEEL
ZmbZIP2 GRELKRQKRKQSNRESARRSRLRKQAEWEEL
GmbZIP1 ERELKRQRRKQSNRESARRSRLRKQAECDEL
GmbZIP4.1 DRELKKQKRKQSNRESARRSRLRKQAECEEL
GmbZIP4.2 DRELKKQKRKQSNRESARRSRLRKQAECEEL
GmbZIP4.3 DRELKKQKRKQSNRESARRSRLRKQAECEEL
GmbZIP4.4 DRELKKQKRKQSNRESARRSRLRKQAECEEL
GmbZIP4.5 DRELKKQKRKQSNRESARRSRLRKQAECEEL
GmbZIP4.6 DRELKKQKRKQSNRESARRSRLRKQAECEEL
GmbZIP7.1 ERELKRQKRKQSNRESARRSRLRKQAECEEL
GmbZIP7.2 ERELKRQKRKQSNRESARRSRLRKQAECEEL
GmbZIP7.3 ERELKRQKRKQSNRESARRSRLRKQAECEEL
GmbZIP7.4 ERELKRQKRKQSNRESARRSRLRKQAECEEL
GmbZIP24.1 ERELKRERRKQSNRESARRSRLRKQAETEEL
GmbZIP24.2 ERELKRERRKQSNRESARRSRLRKQAETEEL
GmbZIP24.3 ERELKRERRKQSNRESARRSRLRKQAETEEL
GmbZIP24.4 ERELKRERRKQSNRESARRSRLRKQAETEEL
GmbZIP24.5 ERELKRERRKQSNRESARRSRLRKQAETEEL
GmbZIP24.6 ERELKRERRKQSNRESARRSRLRKQAETEEL
GmbZIP24.7 ERELKRERRKQSNRESARRSRLRKQAETEEL
GmbZIP49.1 ERELKRERRKQSNRESARRSRLRKQAETEEL
GmbZIP49.2 ERELKRERRKQSNRESARRSRLRKQAETEEL
GmbZIP57.1 ERELKRQRRKQSNRESARRSRLRKQAECDEL
GmbZIP57.2 ERELKRQRRKQSNRESARRSRLRKQAECDEL
GmbZIP57.3 ERELKRQRRKQSNRESARRSRLRKQAECDEL
GmbZIP59 ERELKRQRRKQSNRESARRSRLRKQAECDEL
GmbZIP69.1 ERELKKQKRKQSNRESARRSRLRKQAECEEL
GmbZIP69.2 ERELKKQKRKQSNRESARRSRLRKQAECEEL
GmbZIP69.3 ERELKKQKRKQSNRESARRSRLRKQAECEEL
GmbZIP69.4 ERELKKQKRKQSNRESARRSRLRKQAECEEL
GmbZIP80 TAEEKEQRRKQQKKIASKRSRMKIKMEREKL
GmbZIP109 ERELKRERRKQSNRESARRSRLRKQAETEEL
GmbZIP111 ERELKRQRRKQSNRESARRSRLRKQAECDEL
GmbZIP112.1 ERELKRQKRKQSNRESARRSRLRKQAECEEL
GmbZIP112.2 ERELKRQKRKQSNRESARRSRLRKQAECEEL
GmbZIP112.3 ERELKRQKRKQSNRESARRSRLRKQAECEEL
GmbZIP112.4 ERELKRQKRKQSNRESARRSRLRKQAECEEL
GmbZIP112.5 ERELKRQKRKQSNRESARRSRLRKQAECEEL
GmbZIP132.1 ERELKRERRKQSNRESARRSRLRKQAETEEL
GmbZIP132.2 ERELKRERRKQSNRESARRSRLRKQAETEEL
GmbZIP133.1 KDEIRRERKRQSNRESARRSRMRKEKECEEL
GmbZIP133.2 KDEIRRERKRQSNRESARRSRMRKEKECEEL
MtbZIP5 GDDIRKERKRLSNRKSAKRSKIKKQQECEEL
MtbZIP12 DDERRKERKRLSNRKSAKRSKIKKQKEYEEQ
MtbZIP13 GDEIRKERKRLSNRKSAKRSKIKKQKECEEL
MtbZIP38.1 ERELKRQRRKQSNRESARRSRLRKQAECDEL
MtbZIP38.2 ERELKRQRRKQSNRESARRSRLRKQAECDEL
MtbZIP57.1 ERELKRERRKQSNRESARRSRLRKQAEAEEL
MtbZIP57.2 ERELKRERRKQSNRESARRSRLRKQAEAEEL
MtbZIP57.3 ERELKRERRKQSNRESARRSRLRKQAEAEEL
MtbZIP57.4 ERELKRERRKQSNRESARRSRLRKQAEAEEL
MtbZIP57.5 ERELKRERRKQSNRESARRSRLRKQAEAEEL
MtbZIP57.6 ERELKRERRKQSNRESARRSRLRKQAEAEEL
MtbZIP59 ERELKRERRKQSNRESARRSRLRKQAEAEEL
MtbZIP62.1 ERELKRQRRKQSNRESARRSRLRKQAECDEL
MtbZIP62.2 ERELKRQRRKQSNRESARRSRLRKQAECDEL
MtbZIP63.1 DRELKRQKRKQSNRESARRSRLRKQAECEEL
MtbZIP63.2 DRELKRQKRKQSNRESARRSRLRKQAECEEL
MtbZIP63.3 DRELKRQKRKQSNRESARRSRLRKQAECEEL
MtbZIP63.4 DRELKRQKRKQSNRESARRSRLRKQAECEEL
PvbZIP15 EREIKRQRRKQSNRESARRSRLRKQAECDEL
PvbZIP21 ERELKRQRRKQSNRESARRSRLRKQAECEDL
PvbZIP23 ERELKRQRRKQSNRESARRSRLRKQAECDEL
PvbZIP39.1 ERELKRERRKQSNRESARRSRLRKQAETEEL
PvbZIP39.2 ERELKRERRKQSNRESARRSRLRKQAETEEL
PvbZIP39.3 ERELKRERRKQSNRESARRSRLRKQAETEEL
PvbZIP39.4 ERELKRERRKQSNRESARRSRLRKQAETEEL
PvbZIP63.1 ERELKRERRKQSNRESARRSRLRKQAETEEL
PvbZIP63.2 ERELKRERRKQSNRESARRSRLRKQAETEEL
PvbZIP67 SDEAKEQRRRQSKKKSAKRSRLKMKVERERL
CabZIP19 ERELKRERRKQSNRESARRSRLRKQAEAEEL
CabZIP48 DRELKRQKRKQSNRESARRSRLRKQAECEEL
CabZIP49 ERELKRQRRKQSNRESARRSRLRKQAECDEL
CabZIP50 ERELKRERRKQSNRESARRSRLRKQAEAEEL
CabZIP53 ERELKRQRRKQSNRESARRSRLRKQAECDEL
CcbZIP12 ERELKRERRKQSNRESARRSRLRKQAETEEL
CcbZIP19 ERELKRQRRKQSNRESARRSRLRKQAECDEL
CcbZIP29 ERELKRERRKQSNRESARRSRLRKQAEAEEL
CcbZIP43 ERELKRQKRKQSNRESARRSRLRKQAECEEL
CcbZIP46 ERELKRQRRKQSNRESARRSRLRKQAECDEL
LjbZIP7 DRELKRQKRKQSNRESARRSRLRKQAEAEEL
LjbZIP10 DRELKRQRRKQSNRESARRSRLRKQAECDEL
LjbZIP15 DRELKRERRKQSNRESARRSRLRKQAETEEL
LjbZIP31 ERELKRQRRKQSNRESARRSRLRKQAECDEL

	AtbZIP56	HY5	EKENKRLKRLLRNRVSAQQARERKKAYLSEL
	OsbZIP1		DKEQNRLKRLLRNRVSAQQARERKKAYMTEL
	ZmbZIP31		DKEQNRLKRLLRNRVSAQQARERKKAYLTEL
H	GmbZIP17.1		DKEHRRLKRLLRNRVSAQQARERKKVYVNDL
	GmbZIP17.2		DKEHRRLKRLLRNRVSAQQARERKKVYVNDL
	GmbZIP58		DKESKRLKRLLRNRVSAQQARERKKAYLIDL
	GmbZIP110		DKEHRRLKRLLRNRVSAQQARERKKVYVNDL
	GmbZIP120.1		DKESKRLKRLLRNRVSAQQARERKKAYLIDL
	GmbZIP120.2		DKESKRLKRLLRNRVSAQQARERKKAYLIDL
	MtbZIP20		DKESKRLKRLLRNRVSAQQARERKKAYLSDL
	MtbZIP47		DKEHRRLKRLLRNRVSAQQARERKKLYVNDL
	PvbZIP36.1		DKESKRLKRLLRNRVSAQQARERKKAYLIDL
	PvbZIP36.2		DKESKRLKRLLRNRVSAQQARERKKAYLIDL
	PvbZIP49		--TFDDTNRLLRNRVSAQQARERKKVYVNEL
	PvbZIP62		DKEHRRLKRLLRNRVSAQQARERKKVYVNDL
	CabZIP57		DKEYRRHKRLLRNRVSAQQARERKKVYVNDL
	CabZIP59		DKESKRLKRLLRNRVSAQQARERKKAYLSDL
	CcbZIP25		DKESKRLKRLLRNRVSAQQARERKKAYLIDL
	CcbZIP31		DKEHRRLKRLLRNRVSAQQARERKKVYVNDL
	LjbZIP24		DKESKRLKRLLRNRVSAQQARERKKAYLTDL
	LjbZIP25		DKESKRLKRLLRNRVSAQQARERKKAYLTDL

	AtbZIP59	PosF21	LIDPKRAKRIWANRQSAARSKERKTRYIEEL
	OsbZIP30		LMDPKRAKRILANRQSAARSKERKIRYTSEL
	GmbZIP8		-AIDPKRAKRIANRQSAARSKERKARYITEL
I	GmbZIP8		LMDPKRAKRILANRQSAARSKERKIRYTSEL
	GmbZIP14		TIDPKRAKRILANRQSAARSKERKARYIQEL
	GmbZIP27		STDPKRAKRILANRQSAARSKERKMRYIAEL
	GmbZIP33.1		TADPKRAKRILANRQSAARSKERKACYVLQL
	GmbZIP33.2		TADPKRAKRILANRQSAARSKERKACYVLQL
	GmbZIP65		--DPKRVKRILCNRKSAAKSKERRVIYEKDL
	GmbZIP70		LTDPKRAKRILANRQSAARSKERKMRYISEL
	GmbZIP72		LVDPKRAKRIWANRQSAARSKERKMRYISEL
	GmbZIP76		NIDPKRAKRILANRQSAARSKERKARYIQEL
	GmbZIP78.1		LIDPKRAKRILANRQSAARSKERKMRYISEL
	GmbZIP78.2		LIDPKRAKRILANRQSAARSKERKMRYISEL
	GmbZIP81		LVDPKRAKRIWANRQSAARSKERKMRYISEL
	GmbZIP97		LIDPKRAKRIWANRQSAARSKERKMRYIAEL
	GmbZIP103		TIDPKRAKRILANRQSAARSKERKARYIQEL
	GmbZIP104.1		MADPKRAKRILANRLSAARSKERKMRYISEL
	GmbZIP104.2		MADPKRAKRILANRLSAARSKERKMRYISEL
	GmbZIP105.1		LIDPKRAKRIWANRQSAARSKERKMRYIAEL
	GmbZIP105.2		LIDPKRAKRIWANRQSAARSKERKMRYIAEL
	GmbZIP113		LTDPKRAKRMLANRQSAARSKERKIRYTSEL
	GmbZIP117		MADPKRAKRILANRLSAARSKERKMRYISEL
	GmbZIP119		NIDPKRAKRILANRQSAARSKERKARYIQEL
	MtbZIP4.1		AADPKRAKRILANRQSAARSKERKMKYISEL
	MtbZIP4.2		AADPKRAKRILANRQSAARSKERKMKYISEL
	MtbZIP18		LIDPKRAKRIWANRQSAARSKERKMRYIAEL
	MtbZIP22		TIDPKRAKRILANRQSAARSKERKARYIQEL
	MtbZIP24		TVDPKRAKRILANRQSAARSKERKACYVVEL
	MtbZIP32.1		MADPKRAKRILANRQSAARSKERKMRYISEL
	MtbZIP32.2		MADPKRAKRILANRQSAARSKERKMRYISEL
	MtbZIP40		SVDPKRAKRILANRQSAARSKERKARYIHEL
	MtbZIP44		LIDPKRAKRILANRQSAARSKERKTRYTSEL
	PvbZIP1.1		MADPKRAKRILANRLSAARSKERKMRYISEL
	PvbZIP1.2		MADPKRAKRILANRLSAARSKERKMRYISEL
	PvbZIP1.3		MADPKRAKRILANRLSAARSKERKMRYISEL
	PvbZIP28		LIDPKRAKRILANRQSAARSKERKIRYTSEL
	PvbZIP34		LIDPKRAKRIWANRQSAARSKERKMRYIAEL
	PvbZIP37		NIDPKRAKRILANRQSAARSKERKARYIQEL
	PvbZIP52		TVDPKRAKRILANRQSAARSKERKARYIQEL
	PvbZIP60.1		TVDPKRAKRILANRQSAARSKERKACYVSEL
	PvbZIP60.2		TVDPKRAKRILANRQSAARSKERKACYVSEL
	PvbZIP60.3		TVDPKRAKRILANRQSAARSKERKACYVSEL
	PvbZIP65		LTDPKRAKRILANRQSAARSKERKMRYISEL
	PvbZIP68		LIDPKRAKRIWANRQSAARSKERKMRYISEL
	CabZIP1		LIDPKRAKRIWANRQSAARSKERKMRYIAEL
	CabZIP8		SIDPKRAKRILANRQSAARSKERKARYIQEL
	CabZIP20		MADPKRAKRILANRQSAARSKERKMRYISEL
	CabZIP24		--DPKKLKRILDNRKAAARSKERKKRYQDEL
	CabZIP29		MSDPKRAKRILANRLSAARSKERKMRYISEL
	CabZIP31		TIDPKRAKRILANRQSAARSKERKARYIQEL
	CabZIP36		TVDPKRAKRILANRQSAARSKERKACYVVEL
	CabZIP55		LIDPKRAKRILANRQSAARSKERKTRYTSEL
	CcbZIP3		TIDPKRAKRILANRQSAARSKERKARYIQEL
	CcbZIP14		LSDPKRAKRILANRQSAARSKERKMRYISEL
	CcbZIP18		LIDPKRAKRIWANRQSAARSKERKIRYISEL
	CcbZIP30		TVDPKRAKRILANRQSAARSKERKACYVSEL
	CcbZIP32		NIDPKRAKRILANRQSAARSKERKARYIQEL
	CcbZIP33		LIDPKRAKRILANRQSAARSKERKIRYTSEL
	CcbZIP49		MADPKRAKRILANRLSAARSKERKMRYISEL
	CcbZIP58		STDPKRAKRILANRQSAARSKERKMRYITEL
	LjbZIP1		TSDPKRAKRILANRLSAARSKERKTRYISEL
	LjbZIP13		LIDPKRAKRIWANRQSAARSKERKMRYIAEL
	LjbZIP17		---------ILANRQSAARSKERKMRYISEL
	LjbZIP28		RVDPVRARRIVANRESAARSKERKNRYVSEM
	LjbZIP30		------MNRILANRQSAARSKERKARYIQEL

	AtbZIP2	GBF5	TVDERKRKRMLSNRESARRSRMRKQKHVDDL
	OsbZIP38	LIP19	GADERKRKRMLSNRESARRSRARKQQRLEEL
	ZmbZIP3		TEEERRRNRMTSNRLSARKSRMKRQRHVDDL
S	GmbZIP2		IIDERKHRRMISNRESARRSRMRKQKHLDEL
	GmbZIP5		IMEERKRRRMISNRESARRSRVRKQRHLENL
	GmbZIP6		VMYERKRKRMESNRESARRSRMKKQKQLEDL
	GmbZIP10		IIDERKHRRMISNRESARRSRMRKQKHLDEL
	GmbZIP16		QMDETNMKRRALNREYARQSRLRKHKRLEDL
	GmbZIP22.1		IIDERKRKRMLSNRESARRSRMRKQKQLEDL
	GmbZIP22.2		IIDERKRKRMLSNRESARRSRMRKQKQLEDL
	GmbZIP25		LMEQRKRKRMISNRESARRSRMRKQKHLDDL
	GmbZIP28		ITDERKNKRKQSNRESARRSRMRKRNHLDQL
	GmbZIP30		SPEERKLRRMKSNRESARRSRYRKKQHMENL
	GmbZIP35		LLDDRKKKRMFSNRESARRSRMRKKQQIEVL
	GmbZIP37		MMDERKRRRMISNRESARRSRMRKQRHLENL
	GmbZIP39		IMEQRKRKRMLSNRESARRSRIRKQQHLEGL
	GmbZIP41		MMDQRKRKRMISNRESARRSRMRKQKHLDDL
	GmbZIP43		VVDERKKKRKQSNRESARRSRMRKRKHLDEL
	GmbZIP45		SPEERKLRRMQSNRESARRSRYRKKQHIENL
	GmbZIP52		IMEQRKRKRMLSNRESARRSRMRKQQHLEGL
	GmbZIP56		LINERKHRRMISNRESARRSRMRKQKHLDEL
	GmbZIP60		AMDERKRKRMESNRESARRSRMKKQKLLEDL
	GmbZIP68		VMEERKRRRMISNRESARRSRIRKQRHLENL
	GmbZIP71		MEDQRKRKRMISNRESARRSRMRKQKHLDDL
	GmbZIP79		VMDQRKRKRMISNRESARRSRMRKQKHLDDL
	GmbZIP90		LINERKHRRMLSNRESARRSRMRKQKHLDEL
	GmbZIP98		LMEQRKKKRKQSNRESARRSRMRKQKHLDDL
	GmbZIP99		SSHERKLRRMQSNRESARRSRWRKKRHLENL
	GmbZIP114		AMEERKRRRMISNRESARRSRMRKQRHLENL
	GmbZIP116		LMEQRKKKRKQSNRESARRSRMRKQKHLDDL
	GmbZIP121		LINERKHRRMISNRESARRSRMRKQKHLDEL
	GmbZIP122		LINERKHRRMISNRESARRSRMRKQKHLDEL
	GmbZIP130		MIDERKRKRMLSNRESARRSRMRKQKQLEDL
	MtbZIP2		STRERKIRRMQSNRESARRSRWRKKRHVENL
	MtbZIP3		LMDQRKRKRKQSNRESARRSRMRKQKHMDDL
	MtbZIP8		QIDERKRKRMLSNRESARRSRLRKQQQVEDL
	MtbZIP26		SVKERKLRRMQSNRESAQRSRYKKKKHLETV
	MtbZIP28		-MDQRKNKRKQSNRESAKRCRMRKHKHVDDM
	MtbZIP29		LMDQRKRKRMISNRESARRSRMRKQKHLDDL
	MtbZIP31		LMDQRKRKRMISNRESARRSRMRKQKHLDDL
	MtbZIP36		VVDERKRRRMISNRESARRSRMRKQRHVENL
	MtbZIP37		NMEDRKRRRMISNRESARRSRMRKQRHLENL
	MtbZIP42		LINERKHRRMISNRESARRSRMRKQKHLDEL
	MtbZIP45		LINERKHRRMVSNRESARRSRMRKQKQLDEL
	MtbZIP46		IIDERKHRRMISNRESARRSRMRKQKHLDEL
	MtbZIP54		AMDERKRKRMISNRESARRSRERKQKLLEDY
	MtbZIP58		IIDERKHRRMISNRESARRSRMRKQKHLDEL
	MtbZIP64		ITDQKKRKRMQSNRESARRSRMKKQQHMEDL
	PvbZIP2		LMDQKKKKRKQSNRDSARRSRMRKQKHMDDL
	PvbZIP3		STHERKLRRMQSNRESARRSRWRKKRHLENL
	PvbZIP11		QIDERKRKRMLSNRESARRSRMRKQKQLEDL
	PvbZIP13		LMEDRKRRRMISNRDSARRSRMRKQRHLENL
	PvbZIP14		IIDERKHRRMISNRESARRSRMRKQKHLDEL
	PvbZIP17		MVDQRKRKRMQSNRESARRSRMRKQQHLEGL
	PvbZIP19		LLDDRKKKRMFSNRESARRSRMRKKQQIELL
	PvbZIP20		VVDDRKLRRMISNRESARRSRMRKQRHLENL
	PvbZIP25		LINERKHRRMISNRESARRSRMRKQKHLDEL
	PvbZIP44		AVNERKRKRMESNRESARRSRMRKQKQLETL
	PvbZIP48		LINERKHRRMISNRESARRSRMRKQKHLDEL
	PvbZIP53		VMDERKRKRMISNRESARRSRMRKQKHMDDL
	PvbZIP56		TMDERKNKRKQSNRESARRSRMRKRDHLEDL
	PvbZIP58		SSEEKKVRRMQSNRESARRSRYKKKKHLENL
	PvbZIP61		ALLEDRKKRMFSNRESARRSRMQKKQQIESL
	PvbZIP66		VMDQRKRKRMISNRESARRSRMRKQKHLDDL
	CabZIP7		VLDERRHRRMISNRESARRSRMRKQKHLDEL
	CabZIP10		LINERKHRRMISNRESARRSRMRKQKHLDEL
	CabZIP17		TIDERKRKRMISNRESARRSRMRKQKQLEDL
	CabZIP22		LIDERKRKRMLSNRESARRSRMRKQKQIEDL
	CabZIP28		SIPERKLRRMQSNRESARRSRWRKKRHVENL
	CabZIP32		MMDQRKRKRMISNRESARRSRMRKQKHLDDL
	CabZIP38		VMDQKKRKRMQSNRESARRSRMKKQQHMEDL
	CabZIP44		---------MISNRESARRSRMRKQKHLDEL
	CabZIP45		LMDQRKRKRMISNRESARRSRMRKQKHLDDL
	CabZIP46		VLDDRKKKRMFSNRESARRSRLRKKQQIEVL
	CabZIP47		VTDERKKRRMISNRESARRSRMRKQRHLDNL
	CcbZIP2		LINERKHRRMISNRESARRSRMRKQKHLDEL
	CcbZIP13		MMDQRKRKRMISNRESARRSRMRKQKHLDDL
	CcbZIP17		TVEERKRKRMVSNRESARRSRMRKQQHLEGL
	CcbZIP27		AMDERKRKRMESNRQSARRSRMRKQKQLEDL
	CcbZIP28		LLDDRKKKRMFSNRESARRSRMRKKQQIEVL
	CcbZIP36		LINERKHRRMISNRESARRSRMRKQKHLDEL
	CcbZIP39		-TDPKRAKRILANRQSAARSKERKMRYITEL
	CcbZIP40		-MDEIKRKKRALNREYAKKSRLRKQKQLEDL
	CcbZIP41		QIDERKRKRMLSNRESARRSRMRKQKQLEDL
	CcbZIP44		LMDQRKRKRMISNRESARRSRMRKQKHLDDL
	CcbZIP45		---------MFSNRESARRSRMRKQRHLENL
	CcbZIP47		RAEERKRRRMISNRESARRSRMRKQRHLENL
	CcbZIP51		IIDERKHRRMISNRESARRSRMRKQKHLDEL
	CcbZIP60		LMEQRKKKRKQSNRESARRSRMRRQQHVDDL
	LjbZIP4		LMDQRKRKRMISNRESARRSRMRKQKHLDEL
	LjbZIP9		VIDERKHRRMISNRESARRSRMRKQKHLDEL
	LjbZIP12		IMDERKRRRMISNRESARRSRMRKQRHLENL
	LjbZIP19		VMDQKKRKRMQSNRESARRSRMRKQEHLEGM
	LjbZIP20		LMDQRKRKRKQSNCESARRSRMRKQKHFDDL
	LjbZIP22		AIDDRKRKRMVSNRESARRSRMRKQKQLEDL
	LjbZIP27		SAEERKMRRMQANRESARRSRCKRKKHLEKL
	LjbZIP32		VDNERKRKRILSNRESARRSRMRKQKQLQDL
	LjbZIP33		LINERKHRRMISNRESARRSRMRKQRHLDEL

	AtbZIP62		EREERRIRRILANRESARQTIRRRQAMCEEL
	OsbZIP80	OsZIP-2a	EKEAKRLRRVLANRESARQTILRRQAIRDEL
	ZmbZIP51		EKEAKRLRRVLANRESARQTILRRQAIRDEL
U	GmbZIP12		EPMSKKLKRQLRNRDAAVRSRERKKLYVKNL
	GmbZIP21		EKEVRRIRRILANRESARQTIRRRQALCEEL
	GmbZIP75		DADLKKLRRMESNRLSSRRSwMKKLIYLTNL
	GmbZIP102		QEMERKFRRTISNRFSARRSRLKKLAYMAEL
	GmbZIP128		EKEARRIRRILANRESARQTIRRRQALCEEL
	MtbZIP6.1		EPVSKKQIRQMRNRDAAVKSRERKKVYVKNL
	MtbZIP6.2		EPVSKKQIRQMRNRDAAVKSRERKKVYVKNL
	MtbZIP11		SDEHKTLRRLMQNREAARKSRLRKKAYVQQL
	MtbZIP52		EKEARRIRRVLANRESARQTIRRRQALSEEL
	PvbZIP9.1		EKEARRIRRVLANRESARQTIRRRQALCEEL
	PvbZIP9.2		EKEARRIRRVLANRESARQTIRRRQALCEEL
	PvbZIP46		EPTSKKLKRKLRNRDAAVKSRERKKVYVKNL
	CabZIP15		EKEARRIRRVMANRESARQTIRRRQALSEEL
	CabZIP58		EPVSKKEIRQIRNRDAAVRSRERKKMYVKNL
	CcbZIP57		EPMSKKLKRQLRNRDAAVRSRERKKLYVKDL
