# Supplementary material for: Genome-wide analysis of the basic leucine zipper (bZIP) transcription factor gene family in six legume genomes
Source: BMC Genomics. 2015 Dec 10;16:1053. doi: 10.1186/s12864-015-2258-x (PMC4676100; doi:10.1186/s12864-015-2258-x)
Supplement: Additional file 8: — DNA binding specificity prediction of each group. (DOC 51 kb) [file 12864_2015_2258_MOESM8_ESM.doc]

**Additional file 8.** DNA binding specificity prediction of each group.

| GROUP  (number of legume bZIPs) | Characteristic Features | Putative Binding Site | Known Binding Sites |
| --- | --- | --- | --- |
| A(106) | Conseved motifs M(/K,I,L)I(/M,L)K in the basic region and Q(/L,E)A(/R)Y(/H) in the hinge region | ABREs with a consensus sequence (T/G/C)ACGT(G/T)GC or others containing GCGT or AAGT | CACGTGG/tC, CGCGTG for ABF1[1]；TRAB1/ OsbZIP66 [2] and ZmbZIP72 [3]；GmbZIP1[4] |
| B(7) | Key residues in the basic region RNRE(/D)S(/A)AxxSR and a distinct hinge region KxYVE(/K)E(/N) | G- and C-boxes with equal affinity | Tobacco TGA1b [5, 6] |
| C(33) | Specific hinge region sequence Q(/A,I)A(/T)H(/Q)LT(/A,S)E(/D) | Hybrid ACGT elements like G/C,G/A,C/G boxes | GTGAGTCAT for barley BLZ1 and BLZ2 [7, 8]；Antirrhinum (AmbZIP910) [9]； GATGAPyPuTGPu for Opaque2 [10] |
| D(139) | Conserved residues in positions -21 (L), -20 (A), -19 (Q),-18 (N),-15 (A),-14 (A), -12 (K),-11 (S), and -10 (R).Possess a highly conserved hinge sequence KAYVQQ specific to CBFs | GCC binding C-box sequence | TGACGt/g for tobacco TGA1a [11]； 20 bp ocs-element consensus sequence for OBF3.1 and OBF3.2 [12] |
| E(21) | Basic region has A residue at -19 position and hinge region has a conserved QYISE sequence | Relaxed specificity or may bind to other unknown sequences | AtbZIP34 and AtbZIP61 |
| F(6) | Conserved residue in position -15 (A) specific to CBFs | C-box elements preferentially | unknown |
| G(82) | Conserved residues in positions -18 (N),-15 (S), -14 (A), -11 (S), -10 (R) and has RKQS conserved sequence in the basic region. Have a QAEC(/T,A)E(/D)E hinge sequence specific to GBFs [13] | G-box and/or G-box-like sequences | GCCACGTGGC for GBF1, GBF2 and 3 [14]; AtbZIP16 and AtbZIP68: G-box > Hex > C-box > As-1 [15]； G-box containing sequences for ZmGBF1 [15] |
| G(2) | Conserved residues in positions-18 (N),-15 (S), -14 (A),-11 (S), and -10 (R). | TGACGT G-containing | TGACGT G for snapdragon bZIP910/bZIP911 [9]； Ocs enhancer OCSBF-1 [16]; Wheat histone H3 promoter and the G-box sequence and Adhl promoter for mlip15 [17] |
| G(4) | Conserved Lys substitution at -10 position of the basic region instead of Arg | TGACGT-containing Sequences; some G-box-like sequences | Soybean STF1 [18]； ACACGTGG for HY5 [19] |
| G(2) | Lys(K) instead of Asn(N) at position -18 | Might not be able to bind to DNA as homodimers | unknown |
| H(18) | NRVSAQQAR sequence in their basic region | TGACGT-containing Sequences; some G-box-like sequences | Soybean STF1 [18] ACACGTGG for HY5 [19] |
| I(63) | Conserved Lys(K) substitution at -10 position of the basic region instead of Arg(R) | Sequences other than those containing a palindromic ACGT core | TCCAGCTTGA, TCCAACTTGGA for tobacco RSG [20]; GCTCCGTTG for tomato VSF-1 [21] |
| S(95) | Conserved residues in positions-18 (N),-15 (S), -14 (A),-11 (S), and -10 (R). | TGACGT G-containing | TGACGT G for snapdragon bZIP910/bZIP911 [9]；Ocs enhancer OCSBF-1 [16]; Wheat histone H3 promoter and the G-box sequence and Adhl promoter for mlip15 [17] |
| U(8) | Conserved residue in position -15 (A) specific to CBFs | C-box elements preferentially | Unknown |
| U(6) | Hydrophobic Ile residue at position -10 instead of Arg/Lys | Might not be able to bind DNA or else possess a uniquely different DNA-binding specificity | Corresponds to OsZIP-2a reported earlier [22] |

References：

1. Choi H, Hong J, Ha J, Kang J, Kim SY: **ABFs, a family of ABA-responsive element binding factors**. *J Biol Chem* 2000, **275**(3):1723-1730.

2. Hobo T, Kowyama Y, Hattori T: **A bZIP factor, TRAB1, interacts with VP1 and mediates abscisic acid-induced transcription**. *Proc Natl Acad Sci U S A* 1999, **96**(26):15348-15353.

3. Ying S, Zhang DF, Fu J, Shi YS, Song YC, Wang TY, Li Y: **Cloning and characterization of a maize bZIP transcription factor, ZmbZIP72, confers drought and salt tolerance in transgenic Arabidopsis**. *Planta* 2012, **235**(2):253-266.

4. Gao SQ, Chen M, Xu ZS, Zhao CP, Li L, Xu HJ, Tang YM, Zhao X, Ma YZ: **The soybean GmbZIP1 transcription factor enhances multiple abiotic stress tolerances in transgenic plants**. *Plant Mol Biol* 2011, **75**(6):537-553.

5. Katagiri F, Lam E, Chua NH: **Two tobacco DNA-binding proteins with homology to the nuclear factor CREB**. *Nature* 1989, **340**(6236):727-730.

6. Niu X, Renshaw-Gegg L, Miller L, Guiltinan MJ: **Bipartite determinants of DNA-binding specificity of plant basic leucine zipper proteins**. *Plant Mol Biol* 1999, **41**(1):1-13.

7. Onate L, Vicente-Carbajosa J, Lara P, Diaz I, Carbonero P: **Barley BLZ2, a seed-specific bZIP protein that interacts with BLZ1 in vivo and activates transcription from the GCN4-like motif of B-hordein promoters in barley endosperm**. *J Biol Chem* 1999, **274**(14):9175-9182.

8. Vicente-Carbajosa J, Onate L, Lara P, Diaz I, Carbonero P: **Barley BLZ1: a bZIP transcriptional activator that interacts with endosperm-specific gene promoters**. *Plant J* 1998, **13**(5):629-640.

9. Martinez-Garcia JF, Moyano E, Alcocer MJ, Martin C: **Two bZIP proteins from Antirrhinum flowers preferentially bind a hybrid C-box/G-box motif and help to define a new sub-family of bZIP transcription factors**. *Plant J* 1998, **13**(4):489-505.

10. Lohmer S, Maddaloni M, Motto M, Di Fonzo N, Hartings H, Salamini F, Thompson RD: **The maize regulatory locus Opaque-2 encodes a DNA-binding protein which activates the transcription of the b-32 gene**. *EMBO J* 1991, **10**(3):617-624.

11. Lam E, Lam YK: **Binding site requirements and differential representation of TGF factors in nuclear ASF-1 activity**. *Nucleic Acids Res* 1995, **23**(18):3778-3785.

12. Foley RC, Grossman C, Ellis JG, Llewellyn DJ, Dennis ES, Peacock WJ, Singh KB: **Isolation of a maize bZIP protein subfamily: candidates for the ocs-element transcription factor**. *Plant J* 1993, **3**(5):669-679.

13. Foster R, Izawa T, Chua NH: **Plant bZIP proteins gather at ACGT elements**. *FASEB J* 1994, **8**(2):192-200.

14. Schindler U, Menkens AE, Beckmann H, Ecker JR, Cashmore AR: **Heterodimerization between light-regulated and ubiquitously expressed Arabidopsis GBF bZIP proteins**. *EMBO J* 1992, **11**(4):1261-1273.

15. Shen H, Cao K, Wang X: **AtbZIP16 and AtbZIP68, two new members of GBFs, can interact with other G group bZIPs in Arabidopsis thaliana**. *BMB Rep* 2008, **41**(2):132-138.

16. Singh K, Dennis ES, Ellis JG, Llewellyn DJ, Tokuhisa JG, Wahleithner JA, Peacock WJ: **OCSBF-1, a maize ocs enhancer binding factor: isolation and expression during development**. *Plant Cell* 1990, **2**(9):891-903.

17. Kusano T, Berberich T, Harada M, Suzuki N, Sugawara K: **A maize DNA-binding factor with a bZIP motif is induced by low temperature**. *Mol Gen Genet* 1995, **248**(5):507-517.

18. Cheong YH, Yoo CM, Park JM, Ryu GR, Goekjian VH, Nagao RT, Key JL, Cho MJ, Hong JC: **STF1 is a novel TGACG-binding factor with a zinc-finger motif and a bZIP domain which heterodimerizes with GBF proteins**. *Plant J* 1998, **15**(2):199-209.

19. Chattopadhyay S, Ang LH, Puente P, Deng XW, Wei N: **Arabidopsis bZIP protein HY5 directly interacts with light-responsive promoters in mediating light control of gene expression**. *Plant Cell* 1998, **10**(5):673-683.

20. Fukazawa J, Sakai T, Ishida S, Yamaguchi I, Kamiya Y, Takahashi Y: **Repression of shoot growth, a bZIP transcriptional activator, regulates cell elongation by controlling the level of gibberellins**. *Plant Cell* 2000, **12**(6):901-915.

21. Ringli C, Keller B: **Specific interaction of the tomato bZIP transcription factor VSF-1 with a non-palindromic DNA sequence that controls vascular gene expression**. *Plant Mol Biol* 1998, **37**(6):977-988.

22. Nantel A, Quatrano RS: **Characterization of three rice basic/leucine zipper factors, including two inhibitors of EmBP-1 DNA binding activity**. *J Biol Chem* 1996, **271**(49):31296-31305.
