# Supplementary material for: Genome-wide analysis of the basic leucine zipper (bZIP) transcription factor gene family in six legume genomes
Source: BMC Genomics. 2015 Dec 10;16:1053. doi: 10.1186/s12864-015-2258-x (PMC4676100; doi:10.1186/s12864-015-2258-x)
Supplement: Additional file 9: — Amino acid sequence alignment of the leucine zipper region of 585 legume bZIP proteins. (PDF 4254 kb) [file 12864_2015_2258_MOESM9_ESM.pdf]

Additional file 9. Amino acid sequence alignment of the leucine zipper region of 585 legume bZIP proteins. The legume bZIP proteins are categorized into 44 sub-families (BZ1-BZ44) with similar predicted dimerization properties. The leucine zipper region is divided into heptads (gabcdef) from L0 to L9 to visualize the potential g↔e' pairs. Four colours are used to differentiate between different g↔e' pairs. Attractive basic-acidic pairs (R↔E, K↔E, R↔D and K↔D) are colored orange, attractive acidic-basic pairs (E↔R, E↔K, D↔R, and D↔K) are blue, repulsive basic pairs (K↔K, R↔K, R↔R, Q↔K, R↔Q, and K↔Q) are pink and repulsive acidic pairs (E↔E, E↔D, E↔Q, D↔E, and Q↔E) are green. If single amino acid at the positions e or g is charged, the residue is coloured pink for basic amino acid and green for acidic amino acid. If the a or d position is charged, it is coloured brown. Asparagines at a position are colored red. The prolines and glycines are bold to indicate a potential break in the α-helix. The predicted C-terminal boundary is denoted by the symbol #, other than the natural terminals which are indicated by the symbol *.

Sub-family	bZIP No.	Group	Leucine zipper region									
			L0	L1	L2	L3	L4	L5	L6	L7	L8	L9
			gabcdef	gabcdef	gabcdef	gabcdef	gabcdef	gabcdef	gabcdef	gabcdef	gabcdef	gabcdef
BZ1	CabZIP11	A	YTQELEI	KVSRLEE	ENERLKR	LHGNEWL#	KCLLGGL	QLASACL				
	CabZIP30	A	YTTELEI	KVSRLEE	ENEKLRK	EKELYNM#	LANAPPP	EPKCQLR	RVSSASF			
	CabZIP52	A	YTNELEN	KVSHLEE	ENKKLKR	QKELENM#	LPCEPIE	EPKYQLR	RIASSPF			
	CcbZIP26	A	YTTELEH	KVSRLEE	ENEKLRR	QKVWFFF#	EFLVLFH	SIFSVNS	KC			
	CcbZIP50	A	YHNELVG	KVSRLEE	ENVKLKK	EKVAVSE#	LIHVLFS	PLCSTFL	VFVI			
	GmbZIP107	A	YTNELEN	KVSRLEE	ENERLRR	QKELELM#	LPCEPPP	EPKYQLR	RIASAPF			
	GmbZIP89.2	A	YHNELVS	KVSRLEE	ENVKLKK	EKVLDLI#	HASFSPL	CSAFLVF	VV			
	LjbZIP29	A	YTTELEN	KVSRLEE	ENEKLRK	QKVRFLF#	EFLVLFH	SNFFCLI	LSAECTL	SNISFSL	SSSTE	
	MtbZIP19	A	YTTELEI	KVSRLEE	ENDKLRK	EKELENM#	LANAPPP	EPKCQLR	RVSSASF			
	MtbZIP48	A	YTQELEI	KVSHLEE	ENERLKR	LHEIERV#	LPSMPPP	DPKHQLR	RTSSAPL			
	MtbZIP61	A	YTNELEI	KVSRLEE	ENEMLRK	RKELENM#	LPCAPIA	EPKYQLR	RIASCPF			
	PvbZIP26	A	YTMELEY	KVQQLEQ	ENTHLVN	EEAENRR#	QRRKQLM	ETIIPVE	VMRKPRK	KFGRQNS	F	
	PvbZIP29	A	YTIELEH	KVLRLEE	ENEKLRR	QKEVENI#	LSSVPPQ	KPRYRIH	RSASASF			
BZ2	CabZIP9	A	YHNELVT	KVTLLEQ	ENIKLKK	EKEFEQG#	LPPESSQ	EPKYRLR	RISSALF			
	GmbZIP135	A	YHNELVG	KVSRLEE	ENVKLKK	EKEFEER#	LLPDPLP	ERKYQLR	RHNSAFF			
	GmbZIP32	A	YTTELEH	KVSRLEE	ENEKLRR	QQELEKM#	LSSAPPP	EPRYQIR	RTSSASF			
	GmbZIP48.1	A	YTTELEH	KVSRLEE	ENEKLRR	QQELEKM#	LSSNPPP	EPRYQIR	RTSSASF			
	GmbZIP48.2	A	YTTELEH	KVSRLEE	ENEKLRR	QQELEKM#	LSSNPPP	EPRYQIR	RTSSASF			
	GmbZIP55.1	A	YTNELEN	KVSRLEE	ENERLRK	RKELEQM#	LSCAPPP	EPKYQLR	RIASAPF			
	GmbZIP55.2	A	YTNELEN	KVSRLEE	ENERLRK	RKELEQM#	LSCAPPP	EPKYQLR	RIASAPF			
	GmbZIP89.1	A	YHNELVS	KVSRLEE	ENVKLKK	EKEFEER#	LLPDPLL	EPKYQLR	RHNSAFF			
	GmbZIP89.3	A	YHNELVS	KVSRLEE	ENVKLKK	EKEFEER#	LLPDPLL	EPKYQLR	RHNSAFF			
	GmbZIP89.4	A	YHNELVS	KVSRLEE	ENVKLKK	EKEFEER#	LLPDPLL	EPKYQLR	RHNSAFF			
	LjbZIP14	A	YTNELEN	KVSRLEE	ENERPRK	RKELEQM#	LPCTPPP	EPKYQLR	RIASAPL			
	MtbZIP43	A	YTQELEL	KVSRLEE	ENERLRR	QNEMEKE#	VPTAPPP	EPKNQLR	RTNSASF			
	PvbZIP35	A	YHNELVG	KVSRLEE	ENVKLKK	EKEFEER#	LTPDPLP	EPKYQLR	RHNSAPF			
	PvbZIP5	A	YTHELEI	KVWHLEE	ENERLKR	QNEIEKV#	LPSAPPP	DPKHQLR	RTSSAPL			
	PvbZIP71.1	A	YTNELEH	KVSRLEE	ENERLRK	RQEIEKM#	LPSTPPP	EPKYQLR	RIGSAPF			
	PvbZIP71.2	A	YTNELEH	KVSRLEE	ENERLRK	RQEIEKM#	LPSTPPP	EPKYQLR	RIGSAPF			
	PvbZIP71.3	A	YTNELEH	KVSRLEE	ENERLRK	RQEIEKM#	LPSTPPP	EPKYQLR	RIGSAPF			
BZ3	CabZIP40	A	YTVELEA	EVAKLKE	VNEELQR	KQAEFVE	MQKNKED#	LLRANKI	KYLRRTL	TGPW		
	CcbZIP59	A	YTFELEA	EVAKLKE	LNRELQR	KQAEIME	MQKDKPE#	LDPACRS	RVTKIQC	LRRTLTG	PW	
	GmbZIP11	A	YTFELEA	EVAKLKE	LNRELQR	KQEEIME	MKKNKDL#	DPACRPR	ISKIQCL	RRTLTGP	W	
	GmbZIP50.1	A	YTFELEA	EVAKLKE	LNRELQR	KQEEIME	MQKNKDL#	DPACRPR	VSKIHCL	RRTLTGP	W	
	GmbZIP50.2	A	YTFELEA	EVAKLKE	LNRELQR	KQEEIME	MQKNKDL#	DPACRPR	DDIQENT	KAFSC		
	GmbZIP50.3	A	YTFELEA	EVAKLKE	LNRELQR	KQEEIME	MQKNKDL#	DPACRPR	VSKIHCL	RRTLTGP	W	
	MtbZIP34	A	YTVELEA	EVAKLKE	VNEELQR	KQAEFME	MQKSKED#	LVRTNKI	KYLRRTL	TGPW		
	PvbZIP24.1	A	YTFELEA	EVAKLKE	INRELQR	KQAEIKE	MQKNKQD#	LDPACQS	RVSSRIQ	CLRRTLT	GPW	
	PvbZIP24.2	A	YTFELEA	EVAKLKE	INRELQR	KQAEIKE	MQKNKDL#	DPACQSR	VSSRIQC	LRRTLTG	PW	
BZ4	CabZIP6	A	YTQELEL	KVSRLEE	ENERLRR	QNVCFYA#	FNEYFHD	SGVKLLI	HLDV			
	CcbZIP20	A	YTQELEI	KVSRLEE	ENERLRR	QNVCFNS#	FVSLHHC	FILVC				
	CcbZIP56	A	YTQELEN	KVSQLEE	ENERLRR	LNVC*						
	GmbZIP123	A	YTQELEI	KVSRLEE	ENERLRR	LNEMERA#	LPSVPPP	EPKPKQQ	LRRTSSA	IF		
	GmbZIP124	A	YTQELEI	KVSQLEE	ENERLRR	QNEIERA#	LPSAPPP	DPKHQLR	RTSSAPL			
	GmbZIP15	A	YTQELEI	KVSQLEE	ENERLRR	QNEIERA#	LPSAPSP	DPKHQLR	RTSSAPL			
	GmbZIP36	A	YTQELEI	KVSRLEE	ENERLRR	LNEMERA#	LPSVPPP	EPKPKHQ	LRRTSSA	IF		
	PvbZIP27	A	YTQELEI	KVKQLEV	ENERLRR	LNEIEKA#	LPSEPPP	EPKQQLR	RTSSAIF			
BZ5	CabZIP16	A	YTVELEA	ELNQLRE	ENSQLKQ	ALTELER	RRRQQCS	EETNVRV	QTKAQKA	KEKLRTL#	RRNTSCH	L
	CabZIP4	A	YTVELEA	ELNLLKE	ENDKLKQ	ILAEAKR	KRKQEVS	DYDMFI*				
	CcbZIP10	A	YTVELEA	ELNQLKE	ENAQLKQ	ALAELER	RRKQQCF	EEVNVRV	QTKAQKA	EKKLRAL#	RRNMSSP	L
	CcbZIP61	A	YTVELEA	ELNQLKE	ENAQLKL	AVADLER	RRKQQNL	EEVNGSV	QTNAQKA	KRKLASL#	RRTQSCP	L
	GmbZIP129	A	YTVELEA	ELNQLRE	ENSQLKQ	ALAELER	GRKQQCF	EEVNVSV	KTKAQKA	KEKLRAL#	RRNMSCP	L
	GmbZIP61	A	YTVELEA	ELNQLKE	ENGQLKL	ALADLER	RRKQQHL	DQEVNGR	VQTNAQK	AKKKLRS#	LRKTLSC	PL
	GmbZIP84	A	YTVELEA	ELNLLKE	ENEKLKQ	TLADAER	KRKQEIS	QRKHTTM	AQKRTEK#	LRSMKRP	LSASW	
	GmbZIP91.1	A	YTVELEA	ELNQLKE	ENGQLKL	ALADLER	RRKQQCL	EEVNGRV	QTNAQKA	KKKLRSL#	RKTLSCP	L
	GmbZIP91.2	A	YTVELEA	ELNQLKE	ENGQLKL	ALADLER	RRKQQCL	EEVNGRV	QTNAQKA	KKKLRSL#	RKTLSCP	L
	GmbZIP96	A	YTVELEA	ELNLLKE	ENEKLKQ	TLAEAER	KRKQEIS	QRKHTTM	AQKRTEN#	LRAMRRP	LSASW	
	MtbZIP14	A	YTVELEA	ELNLLKE	ENEKLKQ	VLAEAES	KRKQELL	QRKHSTK	AQKGAEK#	LRAMRRP	ISTTW	
	MtbZIP53	A	YTVELEA	ELNQLRE	ENSQLKQ	ALAELER	RRRQQCS	EETNVRV	QTKAQKA	KEKLRGL#	RRNTSCH	L
	PvbZIP10	A	YTVELEA	ELNQLKE	ENTQLRQ	ALAEMER	RRKQQCF	EELNNIR	VQTNAQK	VKEKMRT#	LRRNMSY	P

	PvbZIP33	A	YTVELEA	ELNHLKD	ENEKLKT	VLAADEL	KRKEA*					
	PvbZIP45	A	YTVELEA	ELNQLKE	ENGELKL	ALADIER	RRKQQNL	EEGNCRV	EKAKKKL	RSLRRTL#	SCPL	
BZ6	CabZIP57	H	YVNDLES	RAKELED	KNSNLDE	RISTLIN	ENTMLRK#	VLMSTRP	KVDESNE	C		
	MtbZIP47	H	YVNDLES	RAKEFED	KNSNLEE	RISTLIN	ENTMLRK#	VLMNTRP	KVDESNE	G		
BZ7	CabZIP28	S	HVENLTS	DLNRLRL	ENRELKN	RLGLTMN	YNLLLST	ENQCLKS	ESMSLIV#	TLLDLYR	TLETMIS	Q
	MtbZIP2	S	HVENLTN	DLNRLRM	ENRELKN	QLGLTMQ	YNLILST	ENERLRL	ESMSLLA#	TLLDLYR	TLETIIS	Q
BZ8	CabZIP58	U	YVKNLET	KSRYFEG	ECRRLEH	LLQCCYA	ENHALRL#	CLQSRGA	FGASMTM	QESAVLL	LGWLHSM	GLIAVM
	CcbZIP57	U	YVKDLEM	KSRYLEG	ECRRLGH	LLQCCYA	ENHALRL#	CLQSRGA	FGASMTT	QESAVLL	LEPLLLG	SLLWFM
	GmbZIP12	U	YVKNLEM	KSRYLEG	ECRRLGH	LLQCCYA	ENNALRL#	CLQLRGT	YGASMTM	QESAVLL	LEPLLLG	SLLWCM
	MtbZIP6.1	U	YVKNLET	KSRYFEG	ECRRLEH	LLQCCYA	ENHALRL#	CLQSRGG	FGAPMTM	QESAVLL	LESLLLG	SLLWFL
	MtbZIP6.2	U	YVKNLET	KSRYFEG	ECRRLEH	LLQCCYA	ENHALRL#	CLQSRGG	FGAPMTM	QESAVLL	LGKNPCC	WVPCYG
	PvbZIP46	U	YVKNLEM	KSRYLEG	ECRRLGH	LLQCCYA	ENNALRL#	CLQSRGA	YGASKTL	QESAVLL	LEPLLLG	SLLWFM
BZ9	MtbZIP41.1	A	YHNELVT	KVTLLEQ	QNMQLKK	EKEFEQG#	LQPESSP	EPKYRLR	RISSAIF			
	MtbZIP41.2	A	YHNELVT	KVTLLEQ	QNMQLKK	EKEFEQG#	LQPESSP	EPKYRLR	RISSAIF			
BZ10	CabZIP39	A	YVSGLED	LVKKLEI	ENKQLFE	EQTERKM	ERLKQLR#	ECIIPIT	EQPRPKP	KLRRTSS	L	
	CcbZIP37	A	YTVELET	LVTHLEE	ENAQLLM	EEVDRRR#	QRYNQLM	ECLIPVE	EKRKPRE	MLRRVNS	AQW	
	GmbZIP38.2	A	YTSELEY	LVHQLEQ	ENARLLK	EEAEMRR#	QRKKHLF	ECIIPIE	VMPKPKK	KLRRVNS	AQSL	
	GmbZIP38.3	A	YTSELEY	LVHQLEQ	ENARLLK	EEAEMRR#	QRKKHVC	TISLSAS	FQLR			
	GmbZIP51	A	YTSELEY	LVHQLEQ	ENVQLLN	EEAEMRR#	QRKKQLF	ECIIPIE	VMPKPKK	KLRRVNS	AQSL	
	GmbZIP64	A	YQVELES	LAVKLEE	ENDKLLK	EKAERKK	KRYKQLM#	EKVLPIA	QKQKPPC	ILRRARS	LQW	
	MtbZIP35	A	YTTELES	LVKHLEI	ENKQLEE	EQAERKK#	LRFKQVC	ISPHFFT	T			
	MtbZIP9.1	A	YQVELES	LAVKLEE	ENDKLMK	EKAERKK	ERFKQLM#	EKVIPVV	EQRRPPR	LLRRVRS	LQW	
	MtbZIP9.2	A	YQVELES	LAVKLEE	ENDKLMK	EKAERKK	ERFKQLK#	IAC				
	MtbZIP9.3	A	YQVELES	LAVKLEE	ENDKLMK	EKAERKK	ERFKQVL#	IYILCYC	LLVCFGP	FGKQYN		
	PvbZIP16	A	YTSELEY	LVHQLEQ	ENARLLN	EEAEMRR#	QRKKQLF	EFIIPVE	EMPKPKK	KLRRVNS	AQSL	
	PvbZIP42	A	YQVELES	LAVKLEE	ENDKLLK	EKAERKK	ERYKQLM#	EKVLPIA	QKQRPPC	TLRRARS	LQW	
BZ11	CabZIP34	A	YTMELEA	EVAKLKE	ENEELQK	KQAEIME	IQKNQVK	EMMNLQR	EVKRKCL#	RRTQTGP	W	
	CcbZIP24	A	YTMELEA	EVAKLKE	ENQELQK	KQEEIME	IQKNQAK	EMMNLQR	EVKRRRL#	RRTQTGP	W	
	GmbZIP29	A	YTMELEA	EVAKLKE	ENQELQK	KQAEIME	IQKNQVK	EMMNLQR	EVKRRRL#	RRTQTGP	W	
	GmbZIP44	A	YTMELEA	EVAKLKE	ENEELQK	KQAEIME	IQKNQVK	EMMNLQR	EVKRRRL#	RRTQTGP	W	
	MtbZIP25.1	A	YTMELEA	EVAKLKE	ENEELQK	KQEEIME	LQKNQVK	EMMNLQR	EVKRKCL#	RRTQTGP	W	
	MtbZIP25.2	A	YTMELEA	EVAKLKE	ENEELQK	KQEEIME	LQKNQVK	EMMNLQR	EVKRKCL#	RRTQTGP	W	
	PvbZIP57.1	A	YTMELEA	EVAKLKE	ENQELQK	KQAEIME	IQKNQVK	EMMNLQR	EVKRRRL#	RRTQTGP	W	
	PvbZIP57.2	A	YTMELEA	EVAKLKE	ENQELQK	KQAEIME	IQKNQVK	EMMNLQR	EVKRRRL#	RRTQTGP	W	
BZ12	CabZIP23	A	YQVELES	LAVKLEE	ENDKLLK	EKAERTK	ERFKQLM#	EKVIPVV	EQRKPPR	LLRRIRS	WQW	
	CcbZIP35	A	YQVELES	LAVRLED	ENDKLIK	EKVAHVF#						
	CcbZIP52	A	YRKGLEA	EVVRLTE	ENSRLRS	QLEELQR	CMWSFDE#	APRKKAP	CRTSSSP	F		
	CcbZIP53	A	YTSELEF	LVHQLEQ	ENARLLN	EEVSLSY#	SCFGGFM	SFSLVKF	RIKRTVL	VGRVVFR		
	GmbZIP101	A	HINRLEK	EKCRLQK	MNSQLKK#	LKLFSAT	TLTPRYQ	LRRTSST	TF			
	GmbZIP106.1	A	YTVELES	LVTHLEE	ENAVLLQ	LAADRKR#	LRLNQLM	ECLIPVE	EKRIPKR	MLRRVNS	SQW	
	GmbZIP106.2	A	YTVELES	LVTHLEE	ENAVLLQ#	LAVCMYL	SIYAFSV	LSCLYNI	LFSLVG			
	GmbZIP26	A	YRKGLEV	EIARLTE	ENSRLKR	QLKELQC	CLSSSDN#	PPTPRMA	ALCRTSS	SPF		
	GmbZIP38.1	A	YTSELEY	LVHQLEQ	ENARLLK	EELFECI#	IPIEVMP	KPKKKLR	RVNSAQS	L		
	GmbZIP38.4	A	YTSELEY	LVHQLEQ	ENARLLK	EEVRF*						
	GmbZIP42	A	YRKGLEV	EISRLTE	ENSRLKR	QLKELQR#	CLCSSHT	PRMAAPC	RTSSSPF			
	GmbZIP54	A	YTVELES	LVTHLEE	ENAILLK	QEADRKR#	QRFNQLM	ECLIPVE	EKRKPKP	MLRRVNS	SQW	
	GmbZIP87	A	HINKLEK	EKCRLQK	INSQLKK#	LSWIFAS	GYKDGRN	NVTSMSV	KPTNNTG	DKKTNKV	FEHVKLH	QGKRVD
	LjbZIP11	A	YTNELKH	KVDHLLE	ENARLKR	QQQELWE	AAAGQQK	KKSNLYR#	SATAPF			
	LjbZIP18	A	YTTELEA	IVQQLEE	ENARLME	EEV*						
	LjbZIP23	A	YQVELES	LAVKLEE	ENDKLLK	EKAERTK	ERFKQLM#	EEVIPVV	EKRRPPR	PLRRINS	MQW	
	LjbZIP26	A	YTLELEA	LVTHLEE	ENAQLLR	EEVCNWD#	VDEIFVE	GMFFLFH	RSSMTGP	VYLVYIK		
	MtbZIP15.1	A	YTIELEA	ELNLLQE	ENKQLKQ	FLAEAER	KRKQELL#	QRKQSAK	VQKGTEK	SSSSTTW		
	MtbZIP15.2	A	YTIELEA	ELNLLQE	ENKQLKQ#	FLVNNTE	IEYTVLV	FFLDDLC	FLMLNIY	IYLNPK		
	PvbZIP51	A	HIHRLEN	EKCRLQK	VNSWLKQ#	LKELDAK	LFSSTTP	TPRYQLR	RSSSTNF			
	PvbZIP54	A	YRRGLEA	EIARLTE	ENSRLRK	QLKELQC	SLSSSEN#	TPDRISA	PCRTSSS	PF		
BZ13	PvbZIP22	A	YLIELKQ	LIELKQK	IEQLQEE	NARLIRQ	QQLLRET	RGNLSRT#	YTAPF			
BZ14	CabZIP12	B	YVEELEE	KVRSMHS	TIADLSS	KITFVMA	ENATLRQ#	QLGGGMM	CPPPPPA	GSGMYPH	PPMPPMP	YPWMPYA
	CcbZIP21	B	YVEELEE	KVRSMNS	IIADLSS	KISYMVA	ENATLRQ#	QVGAGVM	CPPPAAG	MYPHHPP	MAPMPYP	WMPCAP
	GmbZIP125	B	YVEELEE	KVRSLNS	IIADMSS	KMSYVVA	ENATLRQ#	QVGAAGV	MCPPPPA	PAPGMYP	HHPPMAP	MPYPWMP

	GmbZIP18	B	YVEELEE	KVRSLNS	IIADMSS	KMSYMVA	EIATLRQ#	QVGAAAG	VMCPPPP	PPAPGMY	PHHPPMA	PMPYPWM
	LjbZIP5	B	YVEELEE	KVRSMHS	TITDLSS	KISFVMA	ENATLRQ#	QLGAGVM	CAPPPPP	GASGMYP	HPPMPPM	GYPWMP
	MtbZIP49	B	YVEELEE	KVRSMHS	TITDLSS	KITYVMA	ENATLRQ#	QLSGGVM	CPPPPPG	AAMYPPH	PGMAPMP	YAWMPC
	PvbZIP6	B	YVEELEE	KVRSMNS	IIADLSS	KISYMVA	ENATLRQ#	QVGAGVM	CAPPPPA	PGIYPHP	PMAPMPY	PWMPCA
BZ15	CabZIP33	C	HLAELES	QVEKLKL	ENATLYK	QFSDASQ	QFHDADT	NNRVLKS	DVEALRA	KVKLAED#	MVTRSSF	NTTLNN
	CabZIP18	C	HLTDLET	QVSQLRG	ENGSLLK	RLTDITH	KYNDAAV	DNRVLKA	DVQTLRA	KVKMAEE#	TVKRFTG	LNPMFN
	CabZIP21	C	HLTELET	QVSQLRG	ENSTLVK	RLTDVNQ	KYTESAV	DNRVLKA	DVETLRA	KVKMAEE#	TVKRITG	LNPMFH
	CabZIP26	C	HLTDLEV	QVEQLRL	ENASLFK	QLTDASQ	QFREANT	NNRVLKS	DVEALRA	KVKLAED#	MVSRGTL	PTFNNQL
	CcbZIP11	C	HLTELET	QVSQLRG	ENSSLLK	RLTDVSQ	KYGDAAV	DNRVLKA	DVETLRA	KVKMAEE#	TLKRITG	LNPMFH
	CcbZIP5	C	HLTDLET	QVSQLRG	ENSSLLK	RLTDVSQ	KYNDSAV	DNRVLKA	DVETLRA	KVKMAEE#	TVKRITG	LNPMLH
	CcbZIP55	C	HLADLEW	QVERLRL	ENANLFK	QLTDASQ	QFREADT	NNRVLKS	DVEALRA	KVKLAED#	MVTRGSL	PFSNQLH
	CcbZIP7	C	HLADLEL	QVEKLKV	ENATLYK	QFTDASQ	QFREADT	NNRVLKS	DVEALRA	KVKLAED#	MVTRSSF	TTLNTQL
	GmbZIP115.1	C	HLADLEW	QVERLRL	ENANLFK	QLTDASQ	QFREADT	NNRVLKS	DVEALRA	KVKLAED#	MITRGTL	TPTNNQI
	GmbZIP115.2	C	HLADLEW	QVERLRL	ENANLFK	QLTDASQ	QFREADT	NNRVLKS	DVEALRA	KVKLAED#	MITRGTL	TPTNNQI
	GmbZIP131	C	HLTELET	QVSQLRG	ENSSLLK	RFTDVSQ	KYNNAAV	DNRVLKA	DVEALRA	KVKMAEE#	TVKRITG	LNPMLH
	GmbZIP134	C	HLTELGT	QVSQLRG	ENSSLLK	RFIDVSP	KYNNVAI	DNRVLKV	DVEALRA#	KLLMIPA	SQIKSRH	YGEDKQ
	GmbZIP137.1	C	HLTDLET	QVSQLRG	ENSTLLK	RLTDVSQ	KYSDSAV	DNRVLKA	DVETLRT	KVKMAEE#	TVKRITG	LNPLLH
	GmbZIP137.2	C	HLTDLET	QVSQLRG	ENSTLLK	RLTDVSQ	KYSDSAV	DNRVLKA	DVETLRT	KVKLYFI#	LKPTGSF	
	GmbZIP23	C	HLTELET	QVSQLRG	ENSSLLK	RFTDVSQ	KYSNAAV	DNRVLKA	DVEALRA	KVKMAEE#	TVKRITG	LSPMLH
	GmbZIP31.1	C	QLSELEL	QVEKLKV	ENATLYK	QFTDASQ	HFREADT	NNRVLKS	DVEALRA	KVKLAED#	MVTRSSF	TTLNYQL
	GmbZIP31.2	C	QLSELEL	QVEKLKV	ENATLYK	QFTDASQ	HFREADT	NNRVLKS	DVEALRA	KVRKGVV#	TKNKGSL	VFN
	GmbZIP31.3	C	QLSELEL	QVEKLKV	ENATLYK	QFTDASQ	HFREADT	NNRVLKS	DVEALRA	KVKLAED#	MVTRSSF	TTLNYQL
	GmbZIP46.1	C	QLSDLEL	QVEKLKV	ENATLYK	QFTDASQ	HFREADT	NNRVLKS	DVEALRA	KVKLAED#	MVTRSSF	TTLNNQL
	GmbZIP46.2	C	QLSDLEL	QVEKLKV	ENATLYK	QFTDASQ	HFREADT	NNRVLKS	DVEALRA	KVKLAED#	MVTRSSF	TTLNNQL
	GmbZIP63	C	HLTDLET	QVSQLRG	ENSTLLK	RLTDVSQ	KYSDSAV	DNRVLKA	DVETLRA	KVKMAEE#	TVKRITG	LNPMPH
	LjbZIP21	C	HLTDLET	QVSQLKG	ENSSLLK	RFSDVSQ	KFNDSAV	DNRILKA	DVEALRA	KVKMAEE#	TVKRITG	LNPMFH
	LjbZIP3	C	HLAELET	QVEKLKL	ENATLYK	QFTDASQ	QFREADT	NNRVLKS	GVEALRA	KVKLAED#	MVTRSSF	TNQILQN
	MtbZIP1.1	C	HLADLEV	QVEQLRL	ENASLFK	QLTDASQ	QFRDANT	NNRVLKS	DVEALRA	KVKLAED#	MVSRGTL	PTFNNQL
	MtbZIP1.2	C	HLADLEV	QVEQLRL	ENASLFK	QLTDASQ	QFRDANT	NNRVLKS	DVEALRA	KVYIYHF#	TSSSPCI	TIRLQKR
	MtbZIP27	C	HLSELEA	QVEKLKL	ENATLYK	QFTDTSQ	QFHEADT	NNRVLKS	DVEALRA	KVKLAED#	MVTRSSF	TTSLNNQ
	MtbZIP55	C	HLTELET	QVSELRG	ENSSLLK	RLTDVSQ	KFNNSAV	DNRILKA	DVEALRA	KVKMAEE#	TVKRFTG	SNPVFN
	MtbZIP56	C	KNTELET	QVSTLRG	ENSSLLK	HFSDVDQ	KYNNSAV	ENRILNA	DFETIKA#	KVSP		
	MtbZIP7	C	HLTELET	QVSQLRG	ENSTLVK	RLTDVNQ	KYTDSAV	DNRVLKA	DVEALRA	KVKMAEE#	TVKRVTG	LNPLFH
	PvbZIP38	C	HLSDLET	QVSQLRG	ENSSLLK	RLTDVSQ	RYNSAAV	DNRVLKA	DVETLRA	KVKMAEE#	TVKRISG	LSPMFH
	PvbZIP43	C	HLTDLET	QVSQLRG	ENSTLLK	RLTDVSQ	KYSESAV	DNRVLKA	DVETLRA	KVKMAEE#	TVKRITG	LNPMVH
	PvbZIP59	C	QLADLES	QVEKLKV	ENATLYK	QFNDASQ	HFREADT	NNRVLKS	DVEALRA	KVKLAED#	MVTRGSF	TTLNNQL
BZ16	GmbZIP73.1	F	HTAYLEE	EVKKLHM	VNQQLVR	KLQGQAL	LEAELAR	LRSILAR	LKGKIDS#	ELGAWPF	QKQYCNT	SYIFKEG
	GmbZIP73.2	F	HTAYLEE	EVKKLHM	VNQQLVR	KLQGQAL	LEAELAR	LRSILAR	LKGKIDS#	ELGAWPF	QKQYCNT	SYIFKEG
	GmbZIP73.3	F	HTAYLEE	EVKKLHM	VNQQLVR	KLQGQAL	LEAELAR	LRSILAR	LKGKIDS#	ELGAWPF	QKQYCNT	SYIFKEG
BZ17	CabZIP42	F	RAASLEE	EVMKLRA	LNQHLMR	KLQGQAA	LEAEVAR	LKCLLVD	IRGRIEG#	EIGSFPY	QKPTNAN	PAIPNLP
	PvbZIP64.1	F	RAASLED	EVVKLRA	LNQHLMK	KLQSQAG	LEAEIAR	LKCLLVD	IRGRIEG#	EIGSFPY	QKSPNAN	PPAVNLP
	PvbZIP64.2	F	RAASLED	EVVKLRA	LNQHLMK	KLQSQAG	LEAEIAR	LKCLLVD	IRGRIEG#	EIGSFPY	QKSPNAN	PPAVNLP
BZ18	CabZIP49	G	ECDELAQ	RAEVLNE	ENASLRA	ELNRIKS	EYEKILS	ENAALKE#	RLGEIPR	TEDLGPG	QNDQHVE	ATQQSG
	CabZIP53	G	ECDELAQ	RAEVLKE	ENASLRL	EVSRIRS	EYEQLLS	ENTALKE#	KLGEQPG	NDQHVRN	DTQQSGQ	TEAVQGG
	CcbZIP19	G	ECDELAQ	RAEALKE	ENASLRS	EVSRIRS	EYEQLLS	ENAALKE#	RLGEQPA	KDERRSG	RNDPHVG	SDTQQSG
	CcbZIP46	G	ECDELAQ	RAEGLKD	ENATLRS	EVSRIRS	EYEQLLS	ENASLKE#	RLGEIPG	VATPGNE	DLRSGQN	DQHVSKD
	GmbZIP1	G	ECDELAQ	RAEALKE	ENASLRS	EVNRIRS	DYEQLVS	ENSALKE#	RLGELPA	NDDHHRS	CRNDQHV	GNDTQQS
	GmbZIP111	G	ECDELAQ	RAEALKE	ENASLRS	EVSRIRS	EYEQLRS	ENAALKD#	RLGEIPG	VTTPGKE	DLRSGQN	DQHVSND
	GmbZIP57.1	G	ECDELAQ	RAEALKE	ENATLRS	EVSQIRS	EYEQLRS	ENAALKE#	RLGDIPG	VATPGKE	DLRSGQN	DQHVSND
	GmbZIP57.2	G	ECDELAQ	RAEALKE	ENATLRS	EVSQIRS	EYEQLRS	ENAALKE#	RLGDIPG	VATPGKE	DLRSGQN	DQHVSND
	GmbZIP57.3	G	ECDELAQ	RAEALKE	ENATLRS	EVSQIRS	EYEQLRS	ENAALKE#	RLGDIPG	VATPGKE	DLRSGQN	DQHVSND
	GmbZIP59	G	ECDELAQ	RAEALKE	ENASLRS	EVNRIRS	DYEQLLS	ENAALKE#	RLGELPP	NDDHHHR	SGRNDQH	VGNDTQQ
	LjbZIP10	G	ECDELAQ	RAEALKE	ENASLRS	EVSRIRS	DYEQLLT	ENAALKE#	RLGELPG	NDDLRSG	KSDQHTH	DDAQQSG
	LjbZIP31	G	ECDELAQ	RADVLKE	ENASLRS	EVTQIRS	DYEQLLS	ENAVLKV#	ILK			
	MtbZIP38.1	G	ECDELAQ	RADVLKE	ENASLRL	EVNRIRS	EYDQLLS	ENAALKE#	RLGEQPG	NDQHMCN	DTQQSGH	TEVVQDG
	MtbZIP38.2	G	ECDELAQ	RADVLKE	ENASLRL	EVNRIRS	EYDQLLS	ENAALKE#	RLGEQPG	NDQHMCN	DTQQSGH	TEVVQDG
	MtbZIP62.1	G	ECDELAQ	RAEVLNQ	ENASLRA	ELSRIKS	EYEKIRS	ENASLKE#	RLGEIPR	NEDLGEG	QNDQHVG	EGTQ*
	MtbZIP62.2	G	ECDELAQ	RAEVLNQ	ENASLRA	ELSRIKS	EYEKIRS	ENASLKE#	RLGEIPR	NEDLGEG	QNDQHVG	EGTQ*
	PvbZIP15	G	ECDELAQ	RAEALKE	ENASLRS	EVSRIRS	DYEQLLS	ENTALKE#	RLGELPA	NDQHVGN	EAQQNGQ	TEGVQGG
	PvbZIP23	G	ECDELAQ	RADVLKE	ENATLRA	EVSRIRS	EFEQLRS	ENASLKE#	RLGEIPG	VATPGNE	DVRSGQN	DQHVSND
BZ19	CabZIP50	G	EAEELAR	KVDTLIA	ENVSLKS	EMNQLAE	NSEKLKM	ENATLKE	KLKNTQL#	GQTEEII	LNSIDKR	ATPVSTE
	CcbZIP12	G	ETEELAR	KVESLNA	ENVTLKS	EINRLTD	SSEKMRV	ENAALRE	KLRNAHQ#	LGPTQEI	TLNIIDS	QRATPIS
	CcbZIP29	G	EAEELAR	KVEMLTA	ENVTLKL	EITQLTE	SSEQLRM	ENSGLRE	KVRNTQL#	GPREEMI	LTSIDNK	RSTPVST
	CcbZIP43	G	ECEELQK	RVETLGS	ENRILRE	ELQRLSE	ECKKLTS	ENNSIKE	ELERVCG#	PEAVANL	E	

	GmbZIP109	G	ETEELAR	KVEMLTA	ENVSLKS	EITRLTE	GSEQMRM	ENSALRE	KLINTQL#	GPREEIT	LSSIDSK	RAAPVST
	GmbZIP132.1	G	ETEELAR	KVESLNA	ENATLKS	EINRLTE	SSEKMRV	ENATLRG	KLKNAQL#	RQTQEIT	LNIIDSQ	RATPVST
	GmbZIP132.2	G	ETEELAR	KVESLNA	ENATLKS	EINRLTE	SSEKMRV	ENATLRG	KLKNAQL#	RQTQEIT	LNIIDSQ	RATPVST
	GmbZIP24.1	G	ETEELAR	KVESLNA	ENATLKS	EINRLTE	SSEKMRV	ENATLRG	KLKNAQL#	GQTQEIT	LKIIDSQ	RATPVST
	GmbZIP24.2	G	ETEELAR	KVESLNA	ENATLKS	EINRLTE	SSEKMRV	ENATLRG	KLKNAQL#	GQTQEIT	LKIIDSQ	RATPVST
	GmbZIP24.3	G	ETEELAR	KVESLNA	ENATLKS	EINRLTE	SSEKMRV	ENATLRG	KLKNAQL#	GQTQEIT	LKIIDSQ	RATPVST
	GmbZIP24.4	G	ETEELAR	KVESLNA	ENATLKS	EINRLTE	SSEKMRV	ENATLRG	KLKNAQL#	GQTQEIT	LKIIDSQ	RATPVST
	GmbZIP24.5	G	ETEELAR	KVESLNA	ENATLKS	EINRLTE	SSEKMRV	ENATLRG	KLKNAQL#	GQTQEIT	LKIIDSQ	RATPVST
	GmbZIP24.6	G	ETEELAR	KVESLNA	ENATLKS	EINRLTE	SSEKMRV	ENATLRG	KLKNAQL#	GQTQEIT	LKIIDSQ	RATPVST
	GmbZIP24.7	G	ETEELAR	KVESLNA	ENATLKS	EINRLTE	SSEKMRV	ENATLRG	KLKNAQL#	GQTQEIT	LKIIDSQ	RATPVST
	GmbZIP4.1	G	ECEELQK	RVESLRS	ENRILRE	ELQRVSE	ECKKLTS	ENDSIKE	ELERMCG#	SEAIANL	E	
	GmbZIP4.2	G	ECEELQK	RVESLRS	ENRILRE	ELQRVSE	ECKKLTS	ENDSIKE	ELERMCG#	SEAIANL	E	
	GmbZIP4.3	G	ECEELQK	RVESLRS	ENRILRE	ELQRVSE	ECKKLTS	ENDSIKE	ELERMCG#	SEAIANL	E	
	GmbZIP4.4	G	ECEELQK	RVESLRS	ENRILRE	ELQRVSE	ECKKLTS	ENDSIKE	ELERMCG#	SEAIANL	E	
	GmbZIP4.5	G	ECEELQK	RVESLRS	ENRILRE	ELQRVSE	ECKKLTS	ENDSIKE	ELERMCG#	SEAIANL	E	
	GmbZIP4.6	G	ECEELQK	RVESLRS	ENRILRE	ELQRVSE	ECKKLTS	ENDSIKE	ELERMCG#	SEAIANL	E	
	GmbZIP49.1	G	ETEELAR	KVDMLTA	ENVSLKS	EIIQLTE	GSEQMRM	ENSALRE	KLRNTQL#	GQREEII	LSSIESK	RAAPVST
	GmbZIP49.2	G	ETEELAR	KVDMLTA	ENVSLKS	EIIQLTE	GSEQMRM	ENSALRE	KLRNTQL#	GQREEII	LSSIESK	RAAPVST
	GmbZIP69.1	G	ECEELQK	RVESLGS	ENQTLRE	ELQRVSE	ECKKLTS	ENDSIKE	ELERLCG#	PEAVANL	E	
	GmbZIP69.2	G	ECEELQK	RVESLGS	ENQTLRE	ELQRVSE	ECKKLTS	ENDSIKE	ELERLCG#	PEAVANL	E	
	GmbZIP69.3	G	ECEELQK	RVESLGS	ENQTLRE	ELQRVSE	ECKKLTS	ENDSIKE	ELERLCG#	PEAVANL	E	
	GmbZIP69.4	G	ECEELQK	RVESLGS	ENQTLRE	ELQRVSE	ECKKLTS	ENDSIKE	ELERLCG#	PEAVANL	E	
	LjbZIP15	G	ETEELAR	KVEALTA	ENVSLKS	DLNELAE	SSENLRV	ENATLRE	KLNNTQL#	GQTEEII	LNSIDSK	RATPIST
	LjbZIP7	G	EAEELAR	KVESLNA	ENVTLKS	EINRLAE	SSEKVKV	ENATLKE	LFS*			
	MtbZIP59	G	EAEELAR	RVDALTA	ENLALKS	EMNELAE	NSAKLKI	ENATLRE	KLENTQL#	GQTEEII	LNGMDKR	ATPVSTE
	PvbZIP21	G	ECEDLQK	RVETLGS	ENRILRE	ELQRLSE	ECEKLTS	ENNSIKE	ELERMCG#	PEAVANL	G	
	PvbZIP39.1	G	ETEELAR	KVESLNA	ENETLKS	EINRLTE	SSEKMRV	ENATLRE	KLKSAQL#	GQTQEIT	LNIIDSQ	RATPVST
	PvbZIP39.2	G	ETEELAR	KVESLNA	ENETLKS	EINRLTE	SSEKMRV	ENATLRE	KLKSAQL#	GQTQEIT	LNIIDSQ	RATPVST
	PvbZIP39.3	G	ETEELAR	KVESLNA	ENETLKS	EINRLTE	SSEKMRV	ENATLRE	KLKSAQL#	GQTQEIT	LNIIDSQ	RATPVST
	PvbZIP39.4	G	ETEELAR	KVESLNA	ENETLKS	EINRLTE	SSEKMRV	ENATLRE	KLKSAQL#	GQTQEIT	LNIIDSQ	RATPVST
	PvbZIP63.1	G	ETEELAR	KVEMLTA	ENVSLKS	EITQLTE	GSEQMRM	ENSALRE	KLRNTQL#	GQREEII	LDSIDSK	RSTPVST
	PvbZIP63.2	G	ETEELAR	KVEMLTA	ENVSLKS	EITQLTE	GSEQMRM	ENSALRE	KLRNTQL#	GQREEII	LDSIDSK	RSTPVST
	PvbZIP67	G	ERERLNA	SIRNLDV	ENAGLRK	ELRDLID	EYDKLTG	NNDSLMD	ELNEMFG#	QETVMDV	FNMQSAD	SDADDNQ
BZ20	MtbZIP13	G	ECEELCQ	KIDTLKD	ENSVLAQ	TLAELSM	KYLELTN	ENDSIKE	ELVKEYG#	QESIADL	LHMKPT	
	MtbZIP5	G	ECEELYN	KIDTLKD	ENSVLAQ	TLAKLSE	ECLELAN	ENDSIEE	ELVKEYG#	PESIADL	LLIKPA	
	MtbZIP12	G	EYEEQCQ	KINTLKD	ENSVLTH	TLTELSE	KCLELTD	ENDSIEE	ELVRMYG#	PESIADL	LHMKPT	
	GmbZIP133.1	G	ECEELHK	QMEMLKD	ENSVLTQ	RLKSLSE	ECLEICN	ENDAIEE	ELIKMYG#	PESIADL	LLFKPT	
	GmbZIP133.2	G	ECEELHK	QMEMLKD	ENSVLTQ	RLKSLSE	ECLEICN	ENDAIEE	ELIKMYG#	PESIADL	LLFKPT	
BZ21	CabZIP19	G	EAEELAR	KVESLNA	ESASLKS	EINRLAE	SSEKLRM	ENAALRE	KFKIAQL#	GQPKDII	LTSIDSQ	RTTPVST
	GmbZIP80	G	EREKLID	SLESLDD	EKAALRE	ELHDRTI	ECEKIEK	ENNALLA	ELIEEYG	EETIMNL	LNAQSTD#SVAGGGQ	
	MtbZIP57.1	G	EAEELAR	KVESLNA	ESASLRS	EINRLAE	NSERLRM	ENAALKE	KFKIAKL#	GQPKEII	LTNIDSQ	RTTPVST
	MtbZIP57.2	G	EAEELAR	KVESLNA	ESASLRS	EINRLAE	NSERLRM	ENAALKE	KFKIAKL#	GQPKEII	LTNIDSQ	RTTPVST
	MtbZIP57.3	G	EAEELAR	KVESLNA	ESASLRS	EINRLAE	NSERLRM	ENAALKE	KFKIAKL#	GQPKEII	LTNIDSQ	RTTPVST
	MtbZIP57.4	G	EAEELAR	KVESLNA	ESASLRS	EINRLAE	NSERLRM	ENAALKE	KFKIAKL#	GQPKEII	LTNIDSQ	RTTPVST
	MtbZIP57.5	G	EAEELAR	KVESLNA	ESASLRS	EINRLAE	NSERLRM	ENAALKE	KFKIAKL#	GQPKEII	LTNIDSQ	RTTPVST
	MtbZIP57.6	G	EAEELAR	KVESLNA	ESASLRS	EINRLAE	NSERLRM	ENAALKE	KFKIAKL#	GQPKEII	LTNIDSQ	RTTPVST
BZ22	CabZIP59	H	YLSDLES	KVNDLEK	KNSELKE	KLSTLQN	ENQMLRQ#	ILKNTTA	SRRGNNS	GTNNAD		
	CcbZIP25	H	YLIDLET	RVKDLEK	KNTELKE	RLSTLQN	ENQMLRQ#	ILKNTTA	SRRGSNS	GTNNAE		
	GmbZIP120.1	H	YLIDLET	RVKDLEK	KNSELKE	RLSTLQN	ENQMLRQ#	ILKNTTA	SRRGSNS	GTNNAE		
	GmbZIP120.2	H	YLIDLET	RVKDLEK	KNSELKE	RLSTLQN	ENQMLRQ#	ILKNTTA	SRRGSNS	GTNNAE		
	GmbZIP58	H	YLIDLET	RVKDLEK	KNSELKE	RLSTLQN	ENQMLRQ#	ILKNTTA	SRRGSNN	GTNNAE		
	LjbZIP24	H	YLTDLET	KVKDLET	NNSELKE	RLSTLQN	ENQMLRQ#	ILKNTTA	SRRGSNG	GTNNAE		
	LjbZIP25	H	YLTDLET	KVKDLET	NNSELKE	RLSTLQN	ENQMLRQ#	ILKNTTA	SRRGSNG	GTNNAE		
	MtbZIP20	H	YLSDLET	RVNDLEK	KNSELKE	KLSTLQN	ENQMLRQ#	ILKNTTA	SRRGSNG	GSNTAE		
	PvbZIP36.1	H	YLIDLET	RVKDLEK	KNSELKE	RLSTLQN	ENQMLRQ#	ILKNTTA	SRRGSNS	GTNNAE		
	PvbZIP36.2	H	YLIDLET	RVKDLEK	KNSELKE	RLSTLQN	ENQMLRQ#	ILKNTTA	SRRGSNS	GTNNAE		
BZ23	CcbZIP31	H	YVNDLES	RAKELQD	KNAILEE	RISTLIN	ENTMLRK#	VLMNARP	KVDDSNE	QKQDQLS	KS	
	GmbZIP110	H	YVNDLES	RAKELQD	KNAILEE	RISTLIN	ENTMLRK#	VLMNARP	KTDDSIE	QKQDQLS	KS	
	GmbZIP17.1	H	YVNDLES	RAKEMQD	KNAILEE	RISTLIN	ENTMLRK#	VLMNARP	KNDDSIE	QKQDQLS	KS	
	GmbZIP17.2	H	YVNDLES	RAKEMQD	KNAILEE	RISTLIN	ENTMLRK#	VLMNARP	KNDDSIE	QKQDQLS	KS	
	PvbZIP49	H	YVNELES	SAVELEE	KNSNLEE	QISTLIN	ENTMLRK#	ILMNTRA	KVDDN			
	PvbZIP62	H	YVNDLEA	RGKELQD	KNAILEE	RISTLIN	ENTMLRK#	VLMNARP	KVDDNNE	QKQDQLS	KS	
BZ24	CcbZIP39	S	YITELEH	KVQTLQT	ETTTLST	QFTELER	DNTELKN	ENNEYKL	RLQAIEQ	QSQLKDA	LNETLDA	EVRRLR#
	CcbZIP58	I	YITELEH	KVQTLQT	ETTTLST	QFTELER	DNTELKN	ENNEYKL	RLQAIEQ	QSQLKDA	LNETLDA	EVRRLRR#

	GmbZIP33.1	I	YVLQLER	KFQSLQT	EATALCA	RLSLFQR	DTTGLTT	ENTELKL	RLQAMEQ	QANLCDA	LNEALKK	EVDGLKI#
	GmbZIP33.2	I	YVLQLER	KFQSLQT	EATALCA	RLSLFQR	DTTGLTT	ENTELKL	RLQAMEQ	QANLCDA	LKKEVDG	LKIATGE#
	LjbZIP28	I	YVSEMEK	KLQDLQM	QSANLSQ	RLLILKK	DTVDLTT	LQEQLKT#	NLQNMGG	QSQIQFG	MHGFIHY	SGLITSK
BZ25	CabZIP24	I	YQDELEK	NVKALQI	HEDNVTA	EFLKEKE	NVLNLAA	ENRDIKE	CIQIEIQ	KGEMIKA	QIEMLME	ELRMVKI#
	GmbZIP102	U	YMAELES	KAKSFEN	TISLLRE	QIAAEQN	EQKLLQI	EHHTLKF	QMAACEK	QRIVLEA	EFKKNKA	EADRLCE#
	PvbZIP4	C	HLADLEW	QVERLRQ	ENSHLFN	QLNDASQ	QFRDADT	DTNNRVL	KSDVEAL	RAKVKLA	EEMVRRG#	TITPFNN
BZ26	CabZIP17	S	QLEDLSD	EVNKLRI	ENNKLLE	DIKIKEE	ACIENEA	TNGVLRA	QTMELTD	RLRFLNS	MLKFAEE#	VSGLSV
	CabZIP22	S	QIEDLTE	ESGRLRS	DNDRLSR	SIKATEE	AYVEMEA	ANSVIRA	QTMELTE	RLRFLNS	VIEFAED#	VGGSIPV
	CcbZIP17	S	HLEGLSV	EVDRLKK	EKEQMSG	NISMTMR	MLQNVEA	ENAILRA	QMAELTN	SLNSLNE#	IIDFINS	TNSLMLE
	CcbZIP41	S	QLEDLTD	EISGLRG	ANKKLAE	NIKAKEE	ACVETEA	ANSILRA	QTMELAD	RLRFLNS	ILEIAEE#	VGGLSV
	MtbZIP3	S	HMDDLIA	EVERLRN	ENSEILT	RMNMTTQ	HYLKIEA	ENCVLRA	QMCELNQ	RLQSLND#	IINLINI	TTTTNGV
	MtbZIP54	S	LLEDYQD	EANRLRN	ENRRLSE	NIRVREE	GFNANEA	ANGVLRA	QTQELTD	QLKFLKS	IIEKAER#	EKIPKIP
	MtbZIP8	S	QVEDLTG	EAGKLKI	ENDRLAR	SIKATEE	AYLKMEA	ANDVIRA	QTRELEA	QFRFLNS	VIDAAAA	EEANSF*
BZ27	CcbZIP27	S	QLEDLTD	EASRLQV	ANERMVQ	SIKAKEE	AYVEMEA	ANDILRA	QTTELAD	RLRFLNS	ILEIAEE#	VSGLSV
	CcbZIP40	S	QLEDLAH	EVNKLQS	ANKKLVE	IIKAKEE	AYAENKV	ANNILRT	QTMELAD	RLRFLNS	IIQVQDL#KP	
	GmbZIP130	S	QLEDLTD	EVSRLQG	ANKKLAE	NIEAKEE	ACVETEA	ANSILRA	QTMELAD	RLRFLNS	ILEIAEE#	VEGLSV
	GmbZIP16	S	RLEDLTN	EVNMLQS	ANKKLVE	SIKAKEE	AYAETEV	DNNILRA	QTVELTD	RLRFLHS	IIQVAEK#	AKGLSV
	GmbZIP22.1	S	QLEDLTD	EVSRLQS	ANKKLAE	NIEAKEE	ACVETEA	ANSILRA	QTMELAD	RLRFLNS	ILEIAEE#	VEGLSV
	GmbZIP22.2	S	QLEDLTD	EVSRLQS	ANKKLAE	NIEVEGL#	SVEIPEI	PDPLLKP	WQIPHPI	QPIMATA	NMFLR	
	GmbZIP6	S	QLEDLTD	EVSRLEG	ENARLAP#	SIKVKEE	AYVEMEA	ANDILRA	QTMELAD	RLRFLNS	IIEIADE#	VGGESF
	LjbZIP22	S	QLEDLTA	EMSRLQV	SNESLAQ	GIKVKEV	SLIEMEA	ANDILRA	QTMELAD	RLRFLNS	ILEIAEE#	VSGFSV
	LjbZIP32	S	QLQDLTT	EITKLQV	GNRKLVE	DIAAKEK	AFAEVDA	ANAVLRT	QTMELAD	RLRFLNS#	ILEIAQV	SGYSVEI
	PvbZIP11	S	QLEDLTD	EVSRLQG	ANKKLVE	NIKAKEE	ACAETEA	GNSILRV	QTMELTE	RLRFLNS	ILEIAEE#	VGGLSV
	PvbZIP44	S	QLETLTE	EASRLQS	ENAALAQ	SIKAKKE	AYVEMEA	GNDIIRA	QTMELAD	RLRFLNS	ILEIAEA#	AGDLSV
BZ28	CabZIP47	S	HLDNLRN	QLNKCRI	ENRELKN	RLQFILY	HSNRIRT	ENEWLRS	ERTVLSQ	RINNYTQ#	ILVFQQL	QPFSPAW
	LjbZIP12	S	HLENLRN	QVNLFRV	ENREMKS	GLQFLLH	QLNRVRT	ENDWLRS	ERTVLLQ	KLHHISQ#	NLVFQQV	QPFSSAW
	LjbZIP27	S	HLEKLTS	QVNRMRI	ENREMKN	LVAFTVQ	EHLLLSL	HNDELRS	ESMTLLA	ELSDLYA#	ILGTTLL	H
	MtbZIP26	S	HLETVAN	RLNGLRI	ENQELKN	QLAFTMH	HHLLLSL	ENDQLLS	ESVALLA	TLSNLCA#	ILSNSIS	S
	MtbZIP36	S	HVENLRN	QLNKCRM	ENREMKN	RLQFILF	HLNRIRT	ENEWLRS	ERTVLNQ	RINNFTQ#	ILVCQQF	QPFSTAW
BZ29	CabZIP15	U	LSEELSR	KAATLVL	ENENLKR	KKELALK	EYKSLET	TNQLLKA#	QIAKSTN	TEAEKTP	VEQKLSV	AEVAPVH
	GmbZIP128	U	LCEELTR	KAATLVA	ENENLKR	EKELALK	EYESLET	TNKNLKT#	QIAKSIN	TEVEKTP	VEPVSSV	AEITPSS
	GmbZIP21	U	LCEELTR	KAASLVV	ENENLKR	EKQLALN	EYQSLET	TNKHLKA#	QIAKSIN	TEVEKTP	VEPGSSV	AEVTPLS
	MtbZIP52	U	LSEELSR	KAATLAM	ENENLKR	KKELALK	EYQSLET	TNKLLKT#	QIAKSIN	TEVEKTP	VVQELSM	SEVSPAP
	PvbZIP9.1	U	LCEELTN	KAATLVV	ENESLKK	EKDLALK	EYQSLEI	TNKYLKA#	QIAKSIN	TEAEKTP	VEPESSV	AEVTPSS
	PvbZIP9.2	U	LCEELTN	KAATLVV	ENESLKK	EKDLALK	EYQSLEI	TNKYLKA#	QIAKSIN	TEAEKTP	VEPESSV	AEVTPSS
BZ30	GmbZIP30	S	HMENLTS	QLNQLRI	QNRLIKN	QLASTMH	QHLLLSL	HNDHLKS	ESVALMA	TLSDLCG#	ILGTMLA	HY
	PvbZIP58	S	HLENLTS	QMNRLRI	QNRFLKN	RVASTMH	QHLLLSL	HNDHLKS	EAISLMA	TLSDLCG#	LLF	
BZ31	CabZIP10	S	HLDELWS	QVVWLRN	ENHQLLD	KLNSFFE	SHDRVVQ	ENVQLRE	EASELRQ#	MVCDIKL	NSSCLPL	SPLEDVP
	CabZIP44	S	HLDELWS	QVVWLRN	ENQQLIE	KLNHVSE	NHDQVVQ	ENAKLKE	ETLELRQ#	MLRDMQI	QSPFIPS	FSPLEDH
	CabZIP7	S	HLDELWS	QVVRLRT	ENHNLID	KLNHVSE	SHDRVVQ	ENAKLKE	ETFDLRQ#	MVSHMQI	GNSFGCN	MTDFNEV
	CcbZIP2	S	HLDELWS	QVVWLRN	ENHQLMD	KLNHVSE	SHDRVVQ	ENVQLIE	QASELRQ#	MICDMQL	HSPSYTP	PSLSPID
	CcbZIP36	S	HLDELWS	QVVWLRN	ENHQLID	KLNHVSE	SHDQVVQ	ENAQLKE	QASELRQ#	MIRDMQI	HSPCPSF	TPLEDDA
	CcbZIP51	S	HLDELWS	QVVRLRS	ENHNLID	KLNHVSE	SHDRVVQ	ENARLKE	EASDLRQ#	MLADMQI	GTSFACT	MEDLEDL
	GmbZIP10	S	HLDELWS	QVVRLRT	ENHNLID	KLNHVSE	SHDRVLQ	ENARLKE	EASDLRQ#	MLADMQI	GTSFACT	MEDLEDL
	GmbZIP121	S	HLDELWS	QVVWLRN	ENHQLMD	KLNHVSA	SQDKVVQ	ENAQLRE	EASELRQ#	MICDMQL	HSPYHPP	PLSPIDD
	GmbZIP122	S	HLDELWS	QVVWLRN	ENHQLMD	KLNHVSE	SHDQVMQ	ENAQLKE	QALELRQ#	MIRDMQI	HSPCPSS	FITPLED
	GmbZIP2	S	HLDELWS	QVVRLRT	ENHNLID	KLNHVSE	SHDRVLQ	ENARLKE	EASALRQ#	MLADMQI	GTAFACT	MEDLEDL
	GmbZIP56	S	HLDELWS	QVVWLRN	ENHQLMD	KLNHVSE	SHDKVAQ	ENVQLRE	EASELRQ#	MICDMQL	HSPYHPP	PLSPIDD
	GmbZIP90	S	HLDELWS	QVVWLRN	ENHQLID	KLNHVSE	THDQVLQ	ENSQLRE	EASELRQ#	MIRDMQI	HSPCGGP	NSFITPL
	LjbZIP33	S	HLDELWS	QVVWLRN	ENHQLLD	KLSHASE	SHDQVVQ	ENAQLKE	EALELRQ#	MLRDMQI	HSPCPSF	APLEDAY
	LjbZIP9	S	HLDELWS	QVVRLRT	ENHNLVD	RLNHVSE	SHDRALQ	ENARLKE	ETSDLRR#	MVADMQI	GNSFACT	MRDFEEI
	MtbZIP42	S	HLDELWS	QVLWLRN	ENHQLIE	KLNHVSE	NHDQVVQ	ENAQLKE	EALELRQ#	MIKDMQI	HSPLIPS	FSPLDDT
	MtbZIP45	S	QLDELWS	QVVWLRN	ENHQLLD	KLNNFCE	THDKVVQ	ENVQLRE	QASELRQ#	MVCDMQL	HSSCLPL	SPLEDVP
	MtbZIP46	S	HLDELWS	QVVRLRT	ENHNLVD	KLNHVSE	SHDKVVQ	ENARLKE	ETFDLRQ#	MVADMQI	GNSFPCN	MEDLCEI
	MtbZIP58	S	HLDELWS	QVMKLRT	ENHNLVD	KLNHVSE	SHDTVVQ	ENARLKE	ETFDLRQ#	MVADMQI	GNSFPCN	MEDLCEI
	PvbZIP14	S	HLDELWS	QVVRLRT	ENHNLID	KLNHMSD	SHDRVLQ	ENTRLKE	EASDLRQ#	MLADMQI	GTSFACT	MEELEDL
	PvbZIP25	S	HLDELWS	QVMWLRN	ENHQLIE	KLNHVSE	SHDQVLQ	ENAQLKE	EASELRQ#	MMRDMQI	HSPCPSF	TPFQDDV
	PvbZIP48	S	HLDELWS	QVVWLRN	ENHQLMD	KLNHVSE	SHDRVAQ	ENVQLRE	EASELRQ#	MICDMQL	HSPYHPP	PLSPIED
BZ32	GmbZIP45	S	HIENLTS	QLNRLRI	QNRLLKN	QLASTMH	QNLLLSL	HNDHLKS	ESVALMA	TLSDLCG	ILGTMLS	H*
	GmbZIP99	S	HLENLMN	QLNRLRM	ENQKYRN	RLFLTMH	QNLLLSL	ENERLRS	ESMTLMA	RLSDLYQ	ILGTMIS	Q*
	PvbZIP3	S	HLENLTN	QQNRFRM	ENRELKN	RLFLTMH	QNLLLSV	ENERLRS	ESLTLMA	TLSNLYQ	ILGISQ*	

BZ33	CabZIP46	S	QIEVLQY	HVDHLQS	LNHQLSQ	KVIYLLE	CNQQIHQ	QNAQLKD	KVSSLQV#	ALSDSMV	TTGNADI	PHHNPND
	CcbZIP28	S	QIEVLQY	HVDHLQT	LNHQLSQ	KIIYLLE	CNQQINQ	HNAQLKE	KVSTLQV#	ALSELLV	PAGSAEQ	HHHIPNG
	GmbZIP35	S	QIEVLQY	HVDNLQT	LNHQLSQ	KIIYLLE	CNQQIHQ	QNAQLKE	KVSTLQV#	ALSDLLA	TSGSAEQ	PHLIPNG
	PvbZIP19	S	QIELLQY	HVDHLQT	LNHQLSE	KIIYLLE	CNQQIHQ	QNAQLKE	KVSSLQV#	ALSDLLV	PEGGAEQ	PHHIPNG
	PvbZIP61	S	QIESLQC	HLDHLQT	LNRQLSE	KIIYLLE	CNQQILQ	QHNAQLK	DKVSYLQ#	VAVSELL	GPPGHVD	QSNHFPH
BZ34	CabZIP32	S	HLDDLAV	QVGQLRN	ENHQIVT	SVNLTTQ	RYLAVES	ENSVLRA	QLNELNN	RLDSLNE#	IINFLNV	SNGVFEP
	CabZIP45	S	HLDDLVS	QVAKLRK	ENQEILT	SVNITTQ	RCLSVEA	ENSVLRA	QMTELSN	RLESLNE#	IVGVFNA	SNGVFGT
	CcbZIP44	S	HLDDLAS	LVTQLRN	ENHQILA	SLNLTTH	KCLTLES	HNSVLRA	QVNELSH	WLESLNQ#	IIDFLNA	TNGAFGP
	GmbZIP25	S	HLDDLAS	QVTQLRN	ENHQILT	SVNLTTQ	KYLAVEA	ENSVLRA#	QVNELSH	WLESLNE	IIHFLNA	TDGGPPP
	GmbZIP41	S	HLDDLAS	QLTQLRS	QNQQLLT	SVNLTSH	KYLAVEA	ENSVLRA	QVNELSH	RLDSLNQ#	IIHLLNF	FEPDAST
	LjbZIP4	S	HLDELAA	QVAQLRS	ENQQMLT	SLNLTTQ	RFMIIDA	ENSVLRA	QMGELSH	RLESLNE#	IIDFVNA	TNGVFAA
	MtbZIP29	S	HLDDLAV	QLSQLRN	ENQQILT	SVNLTTQ	RFLAVES	ENSVLRA	QLNELNS	RFESLNE#	IINFMNV	ANGVFEP
	MtbZIP31	S	HLDDLVS	QVSKLRK	ENQEILT	SVNITTQ	KYLSVEA	ENSVLRA	QMGELSN	RLESLNE#	IVGALNS	SNGVFGA
	PvbZIP53	S	HMDDLAS	LVTQLRN	ENHQILT	SVNLTTQ	KYLAVES	ENSVLRA	QVSELSH	RLESLNE#	ITNFLNA	TNGVFGP
BZ35	CabZIP38	S	HMEDLNN	QIEYLKK	ENNQISR	NVGVTTQ	MYLNVEG	ENAILRV	QMAELSN	RLQSLND#	IINYIES	SNSLFQ
	CcbZIP13	S	HLDDLVS	QVAQLRK	ENQQILT	SVNITTQ	QYLSVEA	ENSVLRA	QVGELSH	RLDSLNE#	IIEVLNA	STADPML
	CcbZIP45	S	HLENLRN	QVNLFRV	ENRELNN	GLHFLLH	HCNRLRT	ENEWLRS	QRTLLLQ	KLANINQ#	LLLFQQL	QSFSSAW
	CcbZIP47	S	HLENLRN	QLSKCRV	ENRELSN	RLQFVVH	HCNRVRT	ENEWLRS	ERTLLHQ	KVSNLTQ#	ILIFQQL	QPSFSSS
	CcbZIP60	S	HVDDLIA	QVERLKR	ENSIIMA	KVNVTTQ	HYLKVEA	QNLILRA	QKTELTQ	TLQSLND#	ITYLINS	TTTDHYN
	GmbZIP114	S	HLENLRN	QLNKCRV	ENRELSN	RLQFVLH	HLNRLRT	ENEWLHS	ERTLLRQ	KVANLTQ#	ILIFQQF	QTFSPAW
	GmbZIP28	S	HLDQLTK	QLSQLAK	NNGEILA	TIDITTQ	HYLNVEA	ENSILRA	QMGELSQ	RLQSLND#	IVHDIII	NTTTYE
	GmbZIP37	S	HLENLRN	QLNKCRV	ENRELGN	RLQFFLH	HLNRLRT	ENEWLRS	ERTLLRQ	KVANLTQ#	ILIFQQF	QTTFSPA
	GmbZIP39	S	HLEGLSA	QLDQLKK	ENAQINT	NISITTQ	MYLNVEA	ENAILRA	QMGELSN	RLNSLNE#	MISFINS	TNNNCL
	GmbZIP43	S	HLDELTK	QVSQLAK	GNGEILG	TIDITTQ	HYLNVEA	ENSILRA	QMEELSQ	RLQSLND#	IVDGIIN	LTNATTA
	GmbZIP5	S	HLENLRN	QVNLFRV	ENRELNN	GLQFLLH	HGNRLRT	ENEWLRS	ERALLRQ	KLANINQ#	LLLFQQL	QAFSSAW
	GmbZIP52	S	HLEGLSA	QLDQLKK	ENTQMNT	NIGISTQ	LYLNVEA	ENAILRA	QMEELSK	RLNSLNE#	MISLINS	TTTTNN
	GmbZIP60	S	LLEDLSD	VASRLQG	ENVRLAQ	SIKAKEE	AYVEIEA	ANDILRA	QTMELAD	RLRFLNS	ILEIADE#VGGGGE	
	GmbZIP68	S	HLENLRN	QMNLFRV	ENRKLNN	GLQFLLH	HCNRLRT	ENEWLLS	ERPMLRQ	KLANINQ#	ILLFRHL	QPFSSAW
	GmbZIP71	S	HLDDLVS	QVAQLRK	ENQQILT	SVNITTQ	QYLSVEA	ENSVLRA	QVGELSH	RLESLNE#	IVDVLNA	TTTVAGF
	GmbZIP79	S	HLDDLVS	QVAQLRK	ENQQILT	SVNITTQ	QYLSVEA	ENSVLRA	QVGELSH	RLESLNE#	IVDVLNA	TTVAGFG
	LjbZIP19	S	HLEGMSA	QVEQLKK	ENNQIST	NIGVTTQ	MYLNVEA	ENAILRV	QMAELSN	RLQSLNE#	IIHYIES	SNNYLF
	LjbZIP20	S	HFDDLNV	QVERLTK	ENSEILN	RVNLITQ	HYVNVEA	ENCILRA	QMGELSQ	RLQSLNA#	IISLINT	TTTTGI
	MtbZIP28	S	HVDDMMS	QMSQLTK	DNSEILN	SINITTQ	HYLNVEA	ENSILRA	QIGELSQ	RFQSLTH#	QSKCHYQ	WVLFERL
	MtbZIP37	S	HLENLRN	QVNRFRV	ENRELNN	GLQFLLY	QCNRVRT	ENEWLRL	ERTMLGQ	KLSNISQ#	NMVFQPF	SSAWPCN
	MtbZIP64	S	HMEDLSN	QIEQLKK	ENIQIST	NVGVTTQ	MYLNVES	ENAILRV	QMAELSH	RLQSLND#	IIHYIES	SNSLFQ
	PvbZIP13	S	HLENLRN	QTNLFRV	ENRELNS	GLQFLLR	HYNRVRT	ENEWLRS	ERTLLRQ	KLADINQ#	ILLFRQL	QPFSTAW
	PvbZIP17	S	HLEGLSG	QLDELKK	ENNEMNR	NIGMTTQ	VYLKVEA	ENAILRA	QMAELSN	RLNSLNE#	IISFINS	TNYLVW
	PvbZIP2	S	HMDDLTA	QLQRLQT	ENNLLFT	QLNITTQ	YHLKLQA	QNSILMA#	QKTELTQ	TLHSLNH	IINLINK	TTPTNDN
	PvbZIP20	S	HLENLRN	QLSKCRV	ENRELNN	RLQFVLH	HRNRLRT	ENEWLRS	QRTFLLQ	KVANLTQ#	TLIFQQF	QQAISPA
	PvbZIP56	S	HLEDLRK	QVSEFTK	ENREILA	TIDMTTQ	HYLKIEA	ENCVLRA	QMGELDQ	RLQSLND#	MIVDIMN	TTTFYER
	PvbZIP66	S	HLDDLVT	QVAQLRK	ENQQILT	SVNITTQ	QFLSVEA	ENSVLRA	QVGELSH	RLESLNE#	IVEVLNA	GFGNTTF
BZ36	GmbZIP98	S	HLDDLIA	QVDHLKK	QKSLTLM	KVDITTK	HYLEVKA	ENSILWA#	QKTELTQ	SLQSLND	IIDLINT	TNGVYHT
	GmbZIP116	S	HLDDLIA	QVDLLKK	QKSLTLK	KVNITTQ	HCLKVEA	ENSILGA#	QKTELTQ	SLQSLND	IINLINT	TTTSDHN
BZ37	CabZIP29	I	YISELEH	KVQTLQT	ETTTLST	QFTKLQM	DNTELKS	ENNEYKL	RLQSLEQ	QSQLKDA	LNETLDA	EVRRLRR#
	CcbZIP49	I	YISELEL	KVQTLQT	ETTTLST	QFTKLQM	DNSELKS	ENNEYKL	RIQALEQ	QSQLKDA	LNETLDA	EVRRLRR#
	GmbZIP104.1	I	YISELEL	KVQTLQT	ETTTLST	QFTKLQM	NNSELKS	ENNEYKL	RIQALEQ	QSQLKDA	LNETLDA	EVRRLRC#
	GmbZIP104.2	I	YISELEL	KVQTLQT	ETTTLST	QFTKLQM	NNSELKS	ENNEYKL	RIQALEQ	QSQLKDG#	MDMHFSS	TLCISSN
	GmbZIP117	I	YISELEL	KVQTLQT	ETTTLST	QFTKLQM	DNSELKS	ENNEYKL	RIQALEQ	QSQLKDA	LNETLDA	EVRRLRR#
	GmbZIP27	I	YIAELEH	KVQTLQT	ETTTLST	QFTKLQR	DNSDLKS	ENNECKL	RLQAMEQ	QSLLKDA	LNETLDA	EVRRLRR#
	PvbZIP1.1	I	YISELEL	KVQTLQT	ETTTLST	QFTKLQM	DNSELKS	ENNEYKL	RIQAFEQ	QSQLKDA	LNETLDA	EVRRLRR#
	PvbZIP1.2	I	YISELEL	KVQTLQT	ETTTLST	QFTKLQM	DNSELKS	ENNEYKL	RIQAFEQ	QSQLKDA	LNETLDA	EVRRLRR#
	PvbZIP1.3	I	YISELEL	KVQTLQT	ETTTLST	QFTKLQM	DNSELKS	ENNEYKL	RIQAFEQ	QSQLKDA	LNETLDA	EVRRLRR#
BZ38	CabZIP1	I	YIAELER	KVQTLQT	EATSLSA	QLTLLQR	DTNGLNS	ENSELKL	RLQTMEQ	QVHLQDG	KAMTL*	
	CabZIP31	I	YIQELER	KVQTLQT	EATTLSA	QLTLYQR	DTTGLST	ENTELKL	RLQAMEQ	QAHLRDA	LNDALMK	EVERLKI#
	CabZIP36	I	YVVELER	KIHTLQT	EATTLSA	QLNLFQR	DTSGLST	ENTELKL	RLQAMEQ	QAKLCDA	LNEALKK	EVDRLKM#
	CabZIP8	I	YIQELER	KVQTLQT	EATTLSA	QLTLYQR	DTTGLST	ENTELKL	RLQAMEQ	QAHLRDA	LNEALKK	EVERLKV#
	CcbZIP18	I	YISELEG	KVQTLQT	EATSLSA	QLTLLQR	DSNGLTT	ENSELKL	RLQTMEQ	QVHLQNA	LNDALRD	EIQHLKV#
	CcbZIP3	I	YIQELER	KVQTLQT	EATTLSA	QLTLYQR	DTTGLST	ENTELKL	RLQAMEQ	QAQLRDA	LNEALKK	EVERLKV#
	CcbZIP30	I	YVSELER	KFQSLQT	EATALSA	QLSLFQR	DTSGLTT	ENTELKL	RLQAMEQ	QAKLCDA	LNEALKK	EVDRLRF#
	CcbZIP32	I	YIQELER	KVQTLQT	EATTLSA	QLTLYQR	DTTGLSS	ENTELKL	RLQAMEQ	QAQLRDA	LNDALMK	EVERLKI#
	CcbZIP33	I	YTSELER	KVQTLQT	EATNLSA	QLTMLQR	DTTDLTA	ENKELKL	RLEALEQ	EAQLRED	LNEALKE	ELQRLRA#

	GmbZIP103	I	YIQELER	KVQTLQT	EATTLSA	QLTLYQR	DTTGLST	ENTELKL	RLQAMEQ	QAQLRDA	LNEALKK	EVERLKV#
	GmbZIP105.1	I	YIAELER	KVQTLQT	EATSLSA	QLTLLQR	DTNGLNS	ENNELKL	RLQTMEQ	QVHLQDA	LNDALKE	EIQHLKI#
	GmbZIP105.2	I	YIAELER	KVQTLQT	EATSLSA	QLTLLQR	DTNGLNS	ENNELKL	RLQTMEQ	QVHLQDA	LNDALKE	EIQHLKI#
	GmbZIP119	I	YIQELER	KVQTLQT	EATTLSA	QLTLYQR	DTTGLSS	ENTELKL	RLQAMEQ	QAQLRDA	LNDALMK	EVERLKI#
	GmbZIP14	I	YIQELER	KVQTLQT	EATTLSA	QLTLYQR	DTSGLST	ENTELKL	RLQAMEQ	QAQLRDA	LNEALKK	EVERLKV#
	GmbZIP72	I	YISELER	KVQTLQT	EATSLSA	QLTLLQR	DTTGMTA	ENSELKL	RLQTMEQ	QVHLQDA	LNDALKE	EIQHLKA#
	GmbZIP76	I	YIQELEH	KVQTLQT	EATTLSA	QLTLYQR	DTTGLSS	ENTELKL	RLQAMEQ	QAQLRDA	LNDALMK	EVERLKI#
	GmbZIP8	I	YTSELER	KVQTLQT	EATNLSA	QLTMLQR	DTTDLTT	ENKELKL	RLEALEQ	EAQLRED	LNEALKE	ELQRLRA#
	GmbZIP81	I	YISELER	KVQTLQT	EATSLSA	QLTLLQR	DTHGMTA	ENSELKL	RLQTMEQ	QVHLQDA	LNDALKE	EIQHLKA#
	GmbZIP97	I	YIAELER	KVQTLQT	EATSLSA	QLTLLQR	DTNGLNS	ENSELKL	RLQTMEQ	QVHLQDA	LNDALKE	EIQHLKI#
	LjbZIP1	I	YISELEH	KVQTLQT	ETTTLST	QFTKLQM	DHIELKN	ENKEYKL	RLQSLEQ	QSQLKDA	LNETLDA	EVRRLRR#
	LjbZIP13	I	YIAELER	KVQTLQT	EATSLSA	QLTLLQR	DTTGLNS	ENSELKL	RLQTMEQ	QVHLQDG	KLMLL*	
	LjbZIP30	I	YIQELER	KVQTLQT	EATTLSA	QLTLYQR	DTTGLSN	ENTDLKL	RLHAMEQ	QAQLRDA	LNEALKK	EVERLKV#
	MtbZIP18	I	YIAELER	KVQTLQT	EATSLSA	QLTLLQR	DTSGLNS	ENSELKL	RLQTMEQ	QVHLQDA	LNDALKE	EITHLKV#
	MtbZIP22	I	YIQELER	KVQTLQT	EATTLSA	QLTLYQR	DTNGLST	ENTELKL	RLQSMEQ	QAHLRDA	LNDALMK	EVDRLKI#
	MtbZIP24	I	YVVELER	KIHTLQT	EATTLSA	QLNLFQR	DTTGLSS	ENTELKL	RLQTMEQ	QAKLCDA	LNEALKK	EVDRLKL#
	MtbZIP4.1	I	YISELEQ	KVQTLQT	ETTTLST	QFTKLQM	DHQEAKS	ENKEYKL	RLQSLEQ	QSQLKDA	LNETLNA	EVRRLRR#
	MtbZIP4.2	I	YISELEQ	KVQTLQT	ETTTLST	QFTKLQM	DHQEAKS	ENKEYKL	RLQSLEQ	QSQLKDA	LNETLNA	EVRRLRR#
	MtbZIP40	I	YIHELER	KVQTLQT	EATTLSA	QLTLYQR	DTTGLST	ENTELKL	RLQAMEQ	QAHLRDA	LNDALKK	EVERLKV#
	PvbZIP28	I	YTSELER	KVQTLQT	EATNLSA	QLTMLQR	DTTDLTS	ENKELKL	RLEALEQ	EAQLRED	LNEALKE	ELQRLRA#
	PvbZIP34	I	YIAELER	KVQTLQT	EATSLSA	QLSLLQR	DTNGLNS	ENSELKL	RLQTMEQ	QVHLQDA	LNDALKE	EIQHLKI#
	PvbZIP37	I	YIQELER	KVQTLQT	EATTLSA	QLTLYQR	DTTGLSS	ENTELKL	RLQAMEQ	QAQLRDA	LNDALMK	EVERLKI#
	PvbZIP52	I	YIQELER	KVQTLQT	EATTLSA	QLTLYQR	DTTGLST	ENTELKL	RLQAMEQ	QAQLRDA	LNEALKK	EVERLKL#
	PvbZIP60.1	I	YVSELER	KFQSLQT	EATTLSA	QLSLFQR	DTTGLSS	ENTELKL	RLQAMEQ	QAKLCDA	LNEALKK	EVDRLRV#
	PvbZIP60.2	I	YVSELER	KFQSLQT	EATTLSA	QLSLFQR	DTTGLSS	ENTELKL	RLQAMEQ	QAKLCDA	LNEALKK	EVDRLRV#
	PvbZIP60.3	I	YVSELER	KFQSLQT	EATTLSA	QLSLFQR	DTTGLSS	ENTELKL	RLQAMEQ	QAKLCDA	LNEALKK	EVDRLRV#
	PvbZIP68	I	YISELER	KVQTLQT	EATSLSA	QLTLLQR	DTNGLNA	ENGELKL	RLQTMEQ	QVHLQDA	LNDALKE	EIQHLKL#
BZ39	CabZIP20	I	YISELEH	KVQTLQT	EATTLSA	QLTLLQR	DSVGLTN	QNSELKF	RLQSMEQ	QAKLRDA	LNEALTA	EVQRLKI#
	CabZIP55	I	YTSELER	KVQTLQT	EATNLSA	QLTILQR	DTTDLSA	QNKELKM	KLEAFEQ	QAQLRED	LNEALKK	ELQRLRA#
	CcbZIP14	I	YISELEH	KVQTLQT	EATTLSA	QLTLLQR	DSAGLTN	QNSELKF	RLQSMEQ	QAKLRDA	LNEALTA	EVQRLKL#
	GmbZIP113	I	YTSELEK	KVQTLQT	EATNLSA	QLTMLQR	DTTDLTA	QNKELKL	RLQAFEQ	EAQLRED	LNEALKK	ELQRLRV#
	GmbZIP65	I	YEKDLRE	KVPQLAI	QSANLDA	KLRFLAE	DTTNRDV	RLKQLRL	TIDAMRQ	EAQIKDA	LSASVKE	ELRSLRR#
	GmbZIP70	I	YISELEH	KVQTLQT	EATTLSA	QLTLLQR	DSAGLTN	QNSELKF	RLQSMEQ	QAKLRDA	LNEALTA	EVQRLKI#
	GmbZIP78.1	I	YISELEH	KVQTLQT	EATTLSA	QLTLLQR	DSAGLTN	QNSELKF	RLQSMEQ	QAKLRDA	LNEALTA	EVQRLKL#
	GmbZIP78.2	I	YISELEH	KVQTLQT	EATTLSA	QLTLLQR	DSAGLTN	QNSELKF	RLQSMEQ	QAKLRDG#	IPEIMQR	GRPNSLL
	LjbZIP17	I	YISELEH	KVQTLQT	EATTLSA	QLTLLQR	DSVGLTN	QNSELKF	RLQSMEQ	QAKLRD*		
	MtbZIP32.1	I	YISELEH	KVQTLQT	EATTLSA	QLTLLQR	DSVGLTN	QNSELKF	RLQSMEQ	QAKLRDA	LNEALTA	EVQRLKI#
	MtbZIP32.2	I	YISELEH	KVQTLQT	EATTLSA	QLTLLQR	DSVGLTN	QNSELKF	RLQSMEQ	QAKLRDG	ISFNLFN	L*
	MtbZIP44	I	YTSELER	KVQTLQT	EATNLSA	QLTTLQR	DTTDLTA	QNKELKM	KLDAFEQ	QAQLRED	LNEALKK	ELQRLRA#
	PvbZIP65	I	YISELEH	KVQTLQT	EATTLSA	QLTLLQR	DSAGLTN	QNSELKF	RLQSMEQ	QAKLRDA	LNEALTA	EVQRLKI#
BZ40	CabZIP13	D	YVQQLES	SRLKLTQ	LEQELQR#	ARQQGIF	ISSTGEQ	THSMSGN	GAMAFDV	EYARWLE	EHNRQTN	ELRAA
	CabZIP14	D	YIQQLES	SRIRLNQ	MEQELTH#	ARNQGMF	FGGGAIM	GGEQGIP	ISMNSIS	SDAAMFD	VE	
	CabZIP25	D	YVQQLEN	SRVRLAQ	IEQELQQ#	VRQQGVF	VASGVTG	DHGHSVV	GNGNSGT	FAFDMDY	ARWVD	
	CabZIP27	D	YVQQLES	SRLKFTQ	LEQELQR#	ARQQGIF	ISSSGDQ	AHSMSGN	GAMAFDV	EYARWLE	EQNRQIN	ELRGA
	CabZIP3	D	YVQQLES	SRLKLTQ	LEQDLQR#	ARSQGMF	MDWSGGV	GSNISSG	AMFDMEY	ARWLEED	NRLLTEL	RNGLEAA
	CabZIP43	D	YVQQLET	SRLRLAN	LEQDLQR#	ARSQGLF	MGCGGNT	SPGAAIF	DMEYARW	VEEDERQ	LVEVRNG	
	CabZIP51	D	YVQQLES	SRLKLTQ	LEQELQR#	ARQQGVF	ISSSGEQ	THSLSGN	GAMQFDA	EYARWLE	EQNRQIN	ELRAA
	CabZIP54	D	YVQQLET	SRLKLMQ	LEIEIGR#	ARKQGMY	TGNALDT	SFMGSSE	TINPGVV	AFEIEYG	NWVEEQH	RW
	CabZIP56	D	YVQQLES	SRVKLLQ	LEQELQR#	AHQQGIF	IATPGDQ	VHLPVGN	GALAFDM	DYAHWVD	EHQHLLN	DLQSG
	CcbZIP15	D	YVQQLES	SRLKLTH	LEQDLQR#	ARSQGLF	MGCGGAG	GNISSGA	AMFDMEY	ARWLEDD	QRHMAEL	RTGLQAP
	CcbZIP22	D	YVQQLES	SRLKLTQ	LEQELQR#	ARQQGIF	ISSSGDQ	AHTLSGN	GAMQFDA	EYARWLE	EQNRQIN	ELRAA
	CcbZIP23	D	YVQQLES	SRLKLTQ	LEQELQR#	ARQQGIF	ISSSGDQ	AHTLSGN	GISWAMQ	FDAEYAR	WLEEQNR	QTNEL
	CcbZIP4	D	YVQQLES	SRIKLNQ	LEQELQR#	ARSQGIL	MGGNALL	GGEQGIP	MSMNGIS	SEAAMFD	VEYARWL	EEHHRLV
	CcbZIP48	D	YVQQLES	SRLKLTQ	IEQELQR#	ARSQGLF	VDCGGVG	SNVSSGA	AMFDMEY	ARWLEEE	QRLMGEL	RNGLQAP
	CcbZIP54	D	YVQQLEN	SRVKLVQ	LEQELQR#	ARQQGIF	IANQGDQ	GHLAVGN	GALAFDI	DYAHWVD	EHQRMLN	DLRTAVN
	CcbZIP6	D	YVQQLEN	SRVRLAQ	LEQELQR#	ARQQVVS	LVIPYIH	VCIVCTC	ALAFDMD	YSRWVDE	HQ	
	CcbZIP8	D	YVQQLES	SRLKLTQ	LEQELQR#	ARQQGIF	ISSTGDQ	AQSMSGN	GAMAFDV	EYARWLE	EHNRQTN	ELRAA
	CcbZIP9	D	YVQQLES	SRIRLNQ	LEQELQR#	ARSQGMF	LGGGALM	GGEQGLP	VAMNTTS	SEAAMFD	VEYARWL	EEHHRIV
	GmbZIP108.1	D	YVQQLES	SRLKLTQ	LEQELQR#	ARQHGIF	ISSSGDQ	AHTLSGN	GAMQFDA	EYARWLE	EQNRQIN	ELKAA
	GmbZIP108.2	D	YVQQLES	SRLKLTQ	LEQELQR#	ARQHGIF	ISSSGDQ	AHTLSGN	GAMQFDA	EYARWLE	EQNRQIN	ELKAA
	GmbZIP126.1	D	YVQQLES	SRLKLTQ	LEQELQR#	ARQQGIF	ISSTGDQ	AQSMSGN	GAMAFDV	EYARWLE	EHNRQTN	ELRAA
	GmbZIP126.2	D	YVQQLES	SRLKLTQ	LEQELQR#	ARQQGIF	ISSTGDQ	AQSMSGN	GAMAFDV	EYARWLE	EHNRQTN	ELRAA
	GmbZIP126.3	D	YVQQLES	SRLKLTQ	LEQELQR#	ARQQGIF	ISSTGDQ	AQSMSGN	GAMAFDV	EYARWLE	EHNRQTN	ELRAA
	GmbZIP127	D	YVQQLES	SRIRLNQ	LEQELQR#	ARTQGMF	LGGGALL	GGEQGLP	VTMNTIS	TEAAMFD	VEYARWQ	EEHHRIV
	GmbZIP13.1	D	YVQQLES	SRIKLNQ	LEQELQR#	ARAQGIL	MGGGNAL	LGGEQGF	HMAMSGI	SSEAAIF	DV	
	GmbZIP13.2	D	YVQQLES	SRIKLNQ	LEQELQR#	ARAQGIL	MGGGNAL	LGGEQGF	HMAMSGI	SSEAAIF	DV	

GmbZIP136.1	D	YVQQLEN	SRVRLAQ	LEQELQR#	ARQQGAF	IATGIPG	DRGHSSV	ANGALAF	DMDYARW	VDEHQRL	IIDIRSA
GmbZIP136.2	D	YVQQLEN	SRVRLAQ	LEQELQR#	ARQQGAF	IATGIPG	DRGHSSV	ANGALAF	DMDYARW	VDEHQRL	IIDIRSA
GmbZIP138.1	D	YVQQLES	SRLKLTQ	LEQELQR#	ARQQGII	ISNSGDQ	AHSMSGN	GAMAFDV	EYARWLE	EQNRQVN	ELRAA
GmbZIP138.2	D	YVQQLES	SRLKLTQ	LEQELQR#	ARQQGII	ISNSGDQ	AHSMSGN	GAMAFDV	EYARWLE	EQNRQVN	ELRAA
GmbZIP138.3	D	YVQQLES	SRLKLTQ	LEQELQR#	ARQQGII	ISNSGDQ	AHSMSGN	GAMAFDV	EYARWLE	EQNRQVN	ELRAA
GmbZIP19.1	D	YVQQLES	SRLKLTQ	LEQELQR#	SRQQGIF	ISSTGDQ	AQSMSGN	GAMAFDV	EYARWLE	EHNRQTN	ELRAAIN
GmbZIP19.2	D	YVQQLES	SRLKLTQ	LEQELQR#	SRQQGIF	ISSTGDQ	AQSMSGN	GAMAFDV	EYARWLE	EHNRQTN	ELRAAIN
GmbZIP20	D	YVQQLES	SRIRLNQ	LEQELQR#	ARTQGMF	LGGGALL	GGEQGLP	VTMNTIS	TEAAMFD	VEYARWQ	EENHRIV
GmbZIP3.1	D	YVQQLES	SRVKLVQ	LEQELQR#	ARQQGIF	IATPGDQ	GHLAVGN	GALAFDI	DYAHWVD	EHQRLLN	DLRTAVN
GmbZIP3.2	D	YVQQLES	SRVKLVQ	LEQELQR#	ARQQGIF	IATPGDQ	GHLAVGN	GALAFDI	DYAHWVD	EHQRLLN	DLRTAVN
GmbZIP34.1	D	YVKQLES	SRLKLMQ	LELEIGK#	ARKQGLY	MGTALDA	GYIGSTS	ETINPGI	VAFEIEY	GQWVEEQ	ER
GmbZIP34.2	D	YVKQLES	SRLKLMQ	LELEIGK#	ARKQGLY	MGTALDA	GYIGSTS	ETINPGI	VAFEIEY	GQWVEEQ	ER
GmbZIP62	D	YVQQLES	SRIKLNQ	LEQELQR#	SRAQGIL	MGGNTLL	GGEQGFP	MAMSGIS	SEAAMFD	VEYARWL	EEHHRIV
GmbZIP66	D	YVQQLET	SRVRLAQ	LEQELQR#	ARQQGAF	IATGNQG	DRSHSAV	GNGALAF	DMDYARW	FDEHQRL	INDIR
GmbZIP67.1	D	YVQQLES	SRLKLTQ	LEQELQR#	ARQQGIF	ISNSGDQ	AHSMSGN	GAMAFDV	EYARWLE	EQNRQIN	ELRAG
GmbZIP67.2	D	YVQQLES	SRLKLTQ	LEQELQR#	ARQQGIF	ISNSGDQ	AHSMSGN	GAMAFDV	EYARWLE	EQNRQIN	ELRAG
GmbZIP67.3	D	YVQQLES	SRLKLTQ	LEQELQR#	ARQQGIF	ISNSGDQ	AHSMSGN	GAMAFDV	EYARWLE	EQNRQIN	ELRAG
GmbZIP74.1	D	YVQQLES	SRLKLTH	LEQDLQR#	ARSQDEF	MGCGGAG	GSISSGA	AMFDMEY	AKWLEDD	QRHIVEL	RSGLQTP
GmbZIP74.2	D	YVQQLES	SRLKLTH	LEQDLQR#	ARSQDEF	MGCGGAG	GSISSGA	AMFDMEY	AKWLEDD	QRHIVEL	RSGLQTP
GmbZIP82.1	D	YVQQLES	SRLKLTH	LEQDLQR#	ARSQGVF	MGCGGAG	GSLSSGA	AMFDMEY	ARWLEDD	QRHMMEL	RSGLQVP
GmbZIP82.2	D	YVQQLES	SRLKLTH	LEQDLQR#	ARSQGVF	MGCGGAG	GSLSSGA	AMFDMEY	ARWLEDD	QRHMMEL	RSGLQVP
GmbZIP82.3	D	YVQQLES	SRLKLTH	LEQDLQR#	ARSQGVF	MGCGGAG	GSLSSGA	AMFDMEY	ARWLEDD	QRHMMEL	RSGLQVP
GmbZIP82.4	D	YVQQLES	SRLKLTH	LEQDLQR#	ARSQGVF	MGCGGAG	GSLSSGA	AMFDMEY	ARWLEDD	QRHMMEL	RSGLQVP
GmbZIP82.5	D	YVQQLES	SRLKLTH	LEQDLQR#	ARSQGVF	MGCGGAG	GSLSSGA	AMFDMEY	ARWLEDD	QRHMMEL	RSGLQVP
GmbZIP82.6	D	YVQQLES	SRLKLTH	LEQDLQR#	ARSQGVF	MGCGGAG	GSLSSGA	AMFDMEY	ARWLEDD	QRHMMEL	RSGLQVP
GmbZIP85.1	D	YVQQLES	SRLKLNQ	IEQELQR#	ARPQGLF	VDCGGVG	STVSSAG	AAMFDME	YARWLEE	DHRLMGE	LRNGLQA
GmbZIP85.2	D	YVQQLES	SRLKLNQ	IEQELQR#	ARPQGLF	VDCGGVG	STVSSGA	AMFDMEY	ARWLEED	HRLMGEL	RNGLQAP
GmbZIP85.3	D	YVQQLES	SRLKLNQ	IEQELQR#	ARPQGLF	VDCGGVG	STVSSAG	AAMFDME	YARWLEE	DHRLMGE	LRNGLQA
GmbZIP85.4	D	YVQQLES	SRLKLNQ	IEQELQR#	ARPQGLF	VDCGGVG	STVSSAG	AAMFDME	YARWLEE	DHRLMGE	LRNGLQA
GmbZIP85.5	D	YVQQLES	SRLKLNQ	IEQELQR#	ARPQGLF	VDCGGVG	STVSSGA	AMFDMEY	ARWLEED	HRLMGEL	RNGLQAP
GmbZIP85.6	D	YVQQLES	SRLKLNQ	IEQELQR#	ARPQGLF	VDCGGVG	STVSSGA	AMFDMEY	ARWLEED	HRLMGEL	RNGLQAP
GmbZIP9.1	D	YVQQLES	SRVKLVH	LEQELQR#	ARQQGIF	IATPGDQ	GHLAVGN	GALAFDI	DYAHWVD	EHQRLLN	DLRTAIN
GmbZIP9.2	D	YVQQLES	SRVKLVH	LEQELQR#	ARQQGIF	IATPGDQ	GHLAVGN	GALAFDI	DYAHWVD	EHQRLLN	DLRTAIN
GmbZIP92	D	YVQQLES	SRLKLTQ	LEQELQR#	ARQQGIF	ISSSGDQ	AHTLSGN	GAMQFDA	EYARWLE	EQNRQIN	ELRAA
GmbZIP95.1	D	YVQQLES	SRLKLTQ	IEQELQR#	ARSQGLF	VDYGGVG	STVSSAG	AAMFDME	YARWLEE	DHRLMGE	LRNGLQA
GmbZIP95.2	D	YVQQLES	SRLKLTQ	IEQELQR#	ARSQGLF	VDYGGVG	STVSSGA	AMFDMEY	ARWLEED	HRLMGEL	RNGLQAP
GmbZIP95.3	D	YVQQLES	SRLKLTQ	IEQELQR#	ARSQGLF	VDYGGVG	STVSSGA	AMFDMEY	ARWLEED	HRLMGEL	RNGLQAP
LjbZIP6	D	YVQQLES	SRLKLTQ	LEQELQR#	ARQQGIF	ISSTGEQ	AHSMSGN	GAIAFDV	EYARWLE	EHNRHTN	ELRAA
LjbZIP8	D	YVQQLEN	SRLKLVQ	IEQELQR#	ARQQGVF	MASPGDQ	SHLAVGG	ANGASAF	DMEYAHW	VDEHQRL	LTDLR
MtbZIP10	D	YVQQLEN	SRLRLAQ	IEHELQQ#	VRQQGTF	VAPGVTA	DHGHSIV	GNSNAGS	VAFDMDY	AR	
MtbZIP11	U	YVQQLEN	SRLRLAQ	IEHELQQ#	VRQQGTF	VAPGVTA	DHGHSIV	GNSNAGE	VMQP*		
MtbZIP16.1	D	YVQQLES	SRLKLTQ	LEQDLQR#	ARSQGMF	MDWSGGV	GGNISSG	GAMFDME	YGRWLEE	DNRLLTE	LRNGLQA
MtbZIP16.2	D	YVQQLES	SRLKLTQ	LEQDLQR#	ARSQGMF	MDWSGGV	GGNISSG	GAMFDME	YGRWLEE	DNRLLTE	LRNGLQA
MtbZIP21	D	YVQQLEN	SRLRLAQ	IEHELQQ#	VRQQGTF	VGIGVTA	DHGHSIV	GNVMQYF	KPSGSVA	FD	
MtbZIP30.1	D	YVQQLES	SRLRLSS	LEQDLQR#	ARSQGLF	LGCGGGN	ISPGAAM	FDMEYAR	WLEEDQR	HMAELRA	GLQA
MtbZIP30.2	D	YVQQLES	SRLRLSS	LEQDLQR#	ARSQGLF	LGCGGGN	ISPGAAM	FDMEYAR	WLEEDQR	HMAELRA	GLQA
MtbZIP30.3	D	YVQQLES	SRLRLSS	LEQDLQR#	ARSQGLF	LGCGGGN	ISPGAAM	FDMEYAR	WLEEDQR	HMAELRA	GLQA
MtbZIP39.1	D	YVQQLES	SRVKLMQ	LEQELQR#	GMFIANP	GDQGHSS	VGNGALA	FDMEYTH	WVDEHQR		
MtbZIP39.2	D	YVQQLES	SRVKLMQ	LEQELQR#	VREQGMF	IANPGDQ	GHSSVGN	GALAFDM	EYTHWVD	EHQRMLN	DLRSA
MtbZIP39.3	D	YVQQLES	SRVKLMQ	LEQELQR#	GMFIANP	GDQGHSS	VGNGALA	FDMEYTH	WVDEHQR		
MtbZIP39.4	D	YVQQLES	SRVKLMQ	LEQELQR#	VREQGMF	IANPGDQ	GHSSVGN	GALAFDM	EYTHWVD	EHQRMLN	DLRSA
MtbZIP50.1	D	YVQQLES	SRLKLTQ	LEQELQR#	ARQQGIF	ISSTGEQ	THSMSGN	GAMAFDA	EYARWLE	EHNRQTN	ELRAA
MtbZIP50.2	D	YVQQLES	SRLKLTQ	LEQELQR#	ARQQGIF	ISSTGEQ	THSMSGN	GAMAFDA	EYARWLE	EHNRQTN	ELRAA
MtbZIP50.3	D	YVQQLES	SRLKLTQ	LEQELQR#	ARQQGIF	ISSTGEQ	THSMSGN	GAMAFDA	EYARWLE	EHNRQTN	ELRAA
MtbZIP51.1	D	YIQQLES	SRIKLNQ	MEQELHH#	ARNQGMF	FGGGAML	GGEQGLP	SMNTISS	EAAMFDV	EYARWLE	EHHRLVC
MtbZIP51.2	D	YIQQLES	SRIKLNQ	MEQELHH#	ARNQGMF	FGGGAML	GGEQGLP	SMNTISS	EAAMFDV	EYARWLE	EHHRLVC
MtbZIP51.3	D	YIQQLES	SRIKLNQ	MEQELHH#	ARNQGMF	FGGGAML	GGEQGLP	SMNTISS	EAAMFDV	EYARWLE	EHHRLVC
MtbZIP60.1	D	YVQQLES	SRLKLTQ	LEQELQR#	ARQQGVF	ISSSGEQ	THSLSGN	GAMQFDA	EYARWLE	EQNRQIN	ELRAA
MtbZIP60.2	D	YVQQLES	SRLKLTQ	LEQELQR#	ARQQGVF	ISSSGEQ	THSLSGN	GAMQFDA	EYARWLE	EQNRQIN	ELRAA
MtbZIP60.3	D	YVQQLES	SRLKLTQ	LEQELQR#	ARQQGVF	ISSSGEQ	THSLSGN	GAMQFDA	EYARWLE	EQNRQIN	ELRAA
MtbZIP60.4	D	YVQQLES	SRLKLTQ	LEQELQR#	ARQQGVF	ISSSGEQ	THSLSGN	GAMQFDA	EYARWLE	EQNRQIN	ELRAA
MtbZIP60.5	D	YVQQLES	SRLKLTQ	LEQELQR#	ARQQGVF	ISSSGEQ	THSLSGN	GAMQFDA	EYARWLE	EQNRQIN	ELRAA
MtbZIP65	D	YVQQLET	SRIKLNQ	LEQELQR#	ARAQGVL	MGGGAVL	GGDQGIP	ISLNSIS	SEAAMFD	VEYGRWL	EEHHRLV
PvbZIP18	D	YVQQLEN	SRVKLVQ	LEQELQQ#	ARQQGIC	IAAEGDQ	GRLAVGN	GALAFDM	DYAHWVD	EHQRLLN	DLRLAIN
PvbZIP32	D	YVQQLES	SRLKLTQ	IEQELQR#	ARSQGLF	VDCGGVG	GNVSSAG	AAMFDME	YGRWLEE	DHRLMGE	LRNGLQV
PvbZIP40	D	YVQQLES	SRLKLTQ	LEQELQR#	ARQQGVF	ISSSGDQ	AHSMSGN	GAMAFDV	EYARWVE	EQNRQIN	ELRAA
PvbZIP41	D	YVQQLEN	SRVRLAQ	LEQDLQR#	ARQQGVH	IASGVTG	DRGHSVV	GNGALAF	DMDYARW	VDEHQRL	INDLRSA
PvbZIP47.1	D	YVQQLES	SRIKLNQ	LEQELQR#	ARAQGIL	MGGNALL	GGEQGFP	MAMNGIS	SEAAMFD	VEYARWL	EEHQRIV
PvbZIP47.2	D	YVQQLES	SRIKLNQ	LEQELQR#	ARAQGIL	MGGNALL	GGEQGFP	MAMNGIS	SEAAMFD	VEYARWL	EEHQRIV

	PvbZIP55	D	YVKQLES	SRMKLMQ	LELEIGK#	ARKQGLY	MGTALDA	SYIGSTS	ETINPVI	VAFEIEY	GQWVEEQ	HR
	PvbZIP69.1	D	YVQQLES	SRLKLTH	LEQDLQR#	ARSQGVF	MGCGGAG	GSISSGA	AMFDMEY	ARWLEDD	HRHMAEL	RTGLQAP
	PvbZIP69.2	D	YVQQLES	SRLKLTH	LEQDLQR#	ARSQGVF	MGCGGAG	GSISSGA	AMFDMEY	ARWLEDD	HRHMAEL	RTGLQAP
	PvbZIP7	D	YVQQLES	SRLKLTQ	LEQELQR#	ARQQGIF	ISSTGDQ	AQSMSGN	GAMAFDV	EYARWLE	EHNRQTN	ELRAA
	PvbZIP72	D	YVQQLES	SRLKLTQ	LEQELQR#	ARQQGIF	ISSSGDQ	AHTLSGN	GAMQFDA	EYARWLE	EQNRQIN	ELRAA
	PvbZIP8.1	D	YVQQLES	SRVRLNQ	LEQDLQR#	ARTQGMF	LGGGALL	GGEQSLP	VAMNTIS	SEAAMFD	VEYARWV	EEHHRIV
	PvbZIP8.2	D	YVQQLES	SRVRLNQ	LEQDLQR#	ARTQGMF	LGGGALL	GGEQSLP	VAMNTIS	SEAAMFD	VEYARWV	EEHHRIV
BZ41	CabZIP35	D	YVQQLES	SRLKLMQ	LEQELDR#	TRQQGMY	MGGGLDS	NNMCFAG	PVNSGIT	AFEMEYG	HWVDEQN	RQISEMR
	CabZIP37	D	YVQQLES	CRLKLVQ	LEQELDR#	TRQQQEG	LYVAGGL	CSNNLGF	EGSVNSE	ITTFKME	YGNWVEE	QNRQI
	CcbZIP1	D	YVQQLES	SRLKLMQ	LEQELDR#	ARQQGMY	IGGGLDS	NHLGFAG	SVNSGIT	TFETEYG	HWVNEQN	RQ
	CcbZIP38	D	YVQQLET	SRVKLMQ	LELEIEK#	ARKQGLY	IRSAVDV	SYMGSSG	TINPGIT	LFELEYG	HWIEEQD	RR
	GmbZIP100.1	D	YVQQLET	SRVKLMQ	LELEIEK#	ARKQGMY	IRRALDV	SYMGSSA	TVNPARI	TLFELEY	GQWIEEQ	DR
	GmbZIP100.2	D	YVQQLET	SRVKLMQ	LELEIEK#	ARKQGMY	IRRALDV	SYMGSSA	TVNPARI	TLFELEY	GQWIEEQ	DR
	GmbZIP100.3	D	YVQQLET	SRVKLMQ	LELEIEK#	ARKQGMY	IRRALDV	SYMGSSA	TVNPARI	TLFELEY	GQWIEEQ	DR
	GmbZIP100.4	D	YVQQLET	SRVKLMQ	LELEIEK#	ARKQGMY	IRRALDV	SYMGSSA	TVNPARI	TLFELEY	GQWIEEQ	DR
	GmbZIP118.1	D	YVQQLES	SRLKLMQ	LEQELDR#	ARQQGIY	IGGGLDS	NHLGFAG	SVNSGIT	TFEMEYG	HWVNEQN	RQITE
	GmbZIP118.2	D	YVQQLES	SRLKLMQ	LEQELDR#	ARQQGIY	IGGGLDS	NHLGFAG	SVNSGIT	TFEMEYG	HWVNEQN	RQITE
	GmbZIP118.3	D	YVQQLES	SRLKLMQ	LEQELDR#	ARQQGIY	IGGGLDS	NHLGFAG	SVNSGIT	TFEMEYG	HWVNEQN	RQITE
	GmbZIP118.4	D	YVQQLES	SRLKLMQ	LEQELDR#	ARQQGIY	IGGGLDS	NHLGFAG	SVNSGIT	TFEMEYG	HWVNEQN	RQITE
	GmbZIP118.5	D	YVQQLES	SRLKLMQ	LEQELDR#	ARQQGIY	IGGGLDS	NHLGFAG	SVNSGIT	TFEMEYG	HWVNEQN	RQITE
	GmbZIP40.1	D	YVQQLES	CRLKLLQ	LEQEVDH#	AKQQGLY	IGNGLGS	NNLGFAG	SVNSGIT	LFKMEYG	NWVEEQN	RQ
	GmbZIP40.2	D	YVQQLES	CRLKLLQ	LEQEVDH#	AKQQGLY	IGNGLGS	NNLGFAG	SVNSGIT	LFKMEYG	NWVEEQN	RQ
	GmbZIP40.3	D	YVQQLES	CRLKLLQ	LEQEVDH#	AKQQGLY	IGNGLGS	NNLGFAG	SVNSGIT	LFKMEYG	NWVEEQN	RQ
	GmbZIP40.4	D	YVQQLES	CRLKLLQ	LEQEVDH#	AKQQGLY	IGNGLGS	NNLGFAG	SVNSGIT	LFKMEYG	NWVEEQN	RQ
	GmbZIP53.1	D	YVQQLES	CRLKLVQ	LEQEVDH#	AKQQGLY	IGDGLGS	NNLGFAG	SVNSGIT	LFKMEYG	NWLEEQN	RQ
	GmbZIP53.2	D	YVQQLES	CRLKLVQ	LEQEVDH#	AKQQGLY	IGDGLGS	NNLGFAG	SVNSGIT	LFKMEYG	NWLEEQN	RQ
	GmbZIP53.3	D	YVQQLES	CRLKLVQ	LEQEVDH#	AKQQGLY	IGDGLGS	NNLGFAG	SVNSGIT	LFKMEYG	NWLEEQN	RQ
	GmbZIP53.4	D	YVQQLES	CRLKLVQ	LEQEVDH#	AKQQGLY	IGDGLGS	NNLGFAG	SVNSGIT	LFKMEYG	NWLEEQN	RQ
	GmbZIP53.5	D	YVQQLES	CRLKLVQ	LEQEVDH#	AKQQGLY	IGDGLGS	NNLGFAG	SVNSGIT	LFKMEYG	NWLEEQN	RQ
	GmbZIP53.6	D	YVQQLES	CRLKLVQ	LEQEVDH#	AKQQGLY	IGDGLGS	NNLGFAG	SVNSGIT	LFKMEYG	NWLEEQN	RQ
	GmbZIP77.1	D	YVQQLES	SRLKLMQ	LEQELDR#	ARQQGMY	IGGGLDS	NHLGFAG	SVNSGIT	TFEMEYG	HWVNEQN	RQ
	GmbZIP77.2	D	YVQQLES	SRLKLMQ	LEQELDR#	ARQQGMY	IGGGLDS	NHLGFAG	SVNSVAG	ITTFEME	YGHWVNE	QN
	GmbZIP77.3	D	YVQQLES	SRLKLMQ	LEQELDR#	ARQQGMY	IGGGLDS	NHLGFAG	SVNSVAG	ITTFEME	YGHWVNE	QN
	GmbZIP77.4	D	YVQQLES	SRLKLMQ	LEQELDR#	ARQQGMY	IGGGLDS	NHLGFAG	SVNSGIT	TFEMEYG	HWVNEQN	RQ
	GmbZIP77.5	D	YVQQLES	SRLKLMQ	LEQELDR#	ARQQGMY	IGGGLDS	NHLGFAG	SVNSGIT	TFEMEYG	HWVNEQN	RQ
	GmbZIP88.1	D	YVQQLET	SRVKLMQ	LELEIEK#	ARKQQGM	YIRSALD	VSYMGSS	GTINPGI	TLFELEY	AQWIEEQ	DRQNQEL
	GmbZIP88.2	D	YVQQLET	SRVKLMQ	LELEIEK#	ARKQQGM	YIRSALD	VSYMGSS	GTINPGI	TLFELEY	AQWIEEQ	DRQNQEL
	GmbZIP88.3	D	YVQQLET	SRVKLMQ	LELEIEK#	ARKQQGM	YIRSALD	VSYMGSS	GTINPGI	TLFELEY	AQWIEEQ	DRQNQEL
	GmbZIP88.4	D	YVQQLET	SRVKLMQ	LELEIEK#	ARKQQGM	YIRSALD	VSYMGSS	GTINPGI	TLFELEY	AQWIEEQ	DRQNQEL
	GmbZIP88.5	D	YVQQLET	SRVKLMQ	LELEIEK#	ARKQQGM	YIRSALD	VSYMGSS	GTINPGI	TLFELEY	AQWIEEQ	DRQNQEL
	LjbZIP2	D	YVQQLES	SRLKLMQ	LEQELDR#	ARHQGMY	IGGGGLD	SNNMGFA	GCANSGI	TTFEMEY	GNWVEEQ	SRRITEM
	MtbZIP23	D	YVQQLES	SRLKLVQ	LEQELER#	VRQQGMY	MGGGLDS	NNMCFAG	PVNPGIA	AFEMEYG	HWVDEQN	RQ
	PvbZIP12.1	D	YVQQLES	SRLKLMQ	LEQELDR#	ARHQGMY	IGGGLDS	NHMGFSG	SVNSGIT	TFEMEYG	HWVNEQN	RQ
	PvbZIP12.2	D	YVQQLES	SRLKLMQ	LEQELDR#	ARHQGMY	IGGGLDS	NHMGFSG	SVNSGIT	TFEMEYG	HWVNEQN	RQ
	PvbZIP50	D	YVQQLET	SRVKLMQ	LELEIEK#	AKKQSMY	IRSAPDL	SYMGSSG	TINPGIA	LFELEYG	QWIEELC	RQ
BZ42	CabZIP2	E	YISELER	SVTSLQA	EVSVLSP#	RVAFLDH	QRLLLNV	DNSALKQ	RIAALAQ	DKIFKDA	HQDALKR	EIERLR
	CabZIP41	E	YISELER	SVTSLQA	EVSVLSP#	RVAFLDH	QRLLLNV	DNSALKQ	RIAALAQ	DKIFKDA	HQEALKR	EIERLR
	CabZIP5	E	YISELER	SVTSLQT	EVSVLSP#	RVAFLDH	QRLILNV	DNSALKQ	RIAALAQ	DKLFKDD	DHESLYF	HKNNST
	CcbZIP16	E	YISELER	SVTSLQA	EVSVLSP#	RVAFLDH	QRLLLNV	DNSALKQ	RIAALAQ	DKIFKDA	HQEALKR	EIERLR
	CcbZIP34	E	YISELER	SVTSLQA	EVSVLSP#	RVAFLDH	QRLLLNV	DNSALKQ	RIAALAQ	DKIFKDA	HQEALKR	EIERLR
	CcbZIP42	E	YISELER	SVTTLQT	EVSALSP#	RVAFLDH	QRLILNV	DNSALKQ	RIAALAQ	DKIFKDA	HQEALKK	EIERLR
	GmbZIP47.1	E	YISELER	SVTSLQA	EVSVLSP#	RVAFLDH	QRLLLNV	DNSALKQ	RIAALAQ	DKIFKDA	HQEALKR	EIERLR
	GmbZIP47.2	E	YISELER	SVTSLQA	EVSVLSP#	RVAFLDH	QRLLLNV	DNSALKQ	RIAALAQ	DKIFKDA	HQEALKR	EIERLR
	GmbZIP83	E	YISELER	SVTSLQA	EVSVLSP#	RVAFLDH	QRLLLNV	DNSALKQ	RIAALAQ	DKIFKDA	HQEALKR	EIERLR
	GmbZIP86	E	YISELER	SVTSLQA	EVSVLSP#	RVAFLDH	QRLLLNV	DNSALKQ	RIAALAQ	DKIFKDA	HQEALKR	EIERLR
	GmbZIP93	E	YISELER	SVTTLQT	EVSALSP#	RVAFLDH	QRLILNV	DNSALKQ	RIAALAQ	DKIFKDA	HQEALKK	EIERLR
	GmbZIP94.2	E	YISELER	SVTSLQA	EVSVLSP#	RVAFLDH	QRLLLNV	DNSALKQ	RIAALAQ	DKIFKDA	HQEALKR	EIERLR
	LjbZIP16	E	YISELER	SVTSLQA	EVSVLSP#	RVAYLDH	QRLLLNV	DNSALKQ	RIAALAQ	DKIFKDA	HQDALKR	EIERLR
	MtbZIP17.1	E	YISELER	SVTSLQT	EVSVLSP#	RVAFLDH	QRLLLNV	DNSALKQ	RIAALAQ	DKLFKDA	HQEALKR	EIERLR
	MtbZIP17.2	E	YISELER	SVTSLQT	EVSVLSP#	RVAFLDH	QRLLLNV	DNSALKQ	RIAALAQ	DKLFKDA	HQEALKR	EIERLR
	MtbZIP33	E	YISELER	SVTSLQA	EVSVLSP#	RVAYLDH	QRLLLNV	DNSAIKQ	RIAALAQ	DKIFKDA	HQEALKR	EIERLR
	PvbZIP30	E	YISELER	SVTTLQT	EVSALSP#	RVAFLDH	QRLILNV	DNSALKQ	RIAALAQ	DKIFKDA	HQEALKK	EIERLR
	PvbZIP31	E	YISELER	SATSLQA	EVSVLSP#	RVAFLDH	QRLLLNV	DNSALKQ	RIAALAQ	DKIFKDA	HQDALKR	EIERLR
	PvbZIP70.1	E	YISELER	SVTSLQA	EVSVLSP#	RVSFLDH	QRLLLNV	DNSGLKQ	RIAALAQ	DKIFKDA	HQEALKR	EIEKLR
	PvbZIP70.2	E	YISELER	SVTSLQA	EVSVLSP#	RVSFLDH	QRLLLNV	DNSGLKQ	RIAALAQ	DKIFKDA	HQEALKR	EIEKLR
	GmbZIP94.1	E	YISELER	SVTSLQV	WDVT*							

BZ43	CabZIP48	G	ECEELQK	RVEVLGG#	ENRTLRE	ELQKLSE	ECEKLTS	ENNSIKE	ELERLCG	PEVVANL	E	
	GmbZIP112.1	G	ECEELQK	RVESLGG#	ENQTLRE	ELQRLSE	ECEKLTS	ENNSIKE	ELERLCG	PEAVANL	D	
	GmbZIP112.2	G	ECEELQK	RVESLGG#	ENQTLRE	ELQRLSE	ECEKLTS	ENNSIKE	ELERLCG	PEAVANL	D	
	GmbZIP112.3	G	ECEELQK	RVESLGG#	ENQTLRE	ELQRLSE	ECEKLTS	ENNSIKE	ELERLCG	PEAVANL	D	
	GmbZIP112.4	G	ECEELQK	RVESLGG#	ENQTLRE	ELQRLSE	ECEKLTS	ENNSIKE	ELERLCG	PEAVANL	D	
	GmbZIP112.5	G	ECEELQK	RVESLGG#	ENQTLRE	ELQRLSE	ECEKLTS	ENNSIKE	ELERLCG	PEAVANL	D	
	GmbZIP7.1	G	ECEELQK	RVESLGG#	ENQTLRD	ELQRLSE	ECEKLTS	ENNSIKE	ELERLCG	PEAVANL	G	
	GmbZIP7.2	G	ECEELQK	RVESLGG#	ENQTLRD	ELQRLSE	ECEKLTS	ENNSIKE	ELERLCG	PEAVANL	G	
	GmbZIP7.3	G	ECEELQK	RVESLGG#	ENQTLRD	ELQRLSE	ECEKLTS	ENNSIKE	ELERLCG	PEAVANL	G	
	GmbZIP7.4	G	ECEELQK	RVESLGG#	ENQTLRD	ELQRLSE	ECEKLTS	ENNSIKE	ELERLCG	PEAVANL	G	
	MtbZIP63.1	G	ECEELQK	RVEALGG#	ENRTLRE	ELQKLSE	ECEKLTS	ENDSIKE	DLERLCG	PEVVANL	E	
	MtbZIP63.2	G	ECEELQK	RVEALGG#	ENRTLRE	ELQKLSE	ECEKLTS	ENDSIKE	DLERLCG	PEVVANL	E	
	MtbZIP63.3	G	ECEELQK	RVEALGG#	ENRTLRE	ELQKLSE	ECEKLTS	ENDSIKE	DLERLCG	PEVVANL	E	
	MtbZIP63.4	G	ECEELQK	RVEALGG#	ENRTLRE	ELQKLSE	ECEKLTS	ENDSIKE	DLERLCG	PEVVANL	E	
BZ44	GmbZIP75	U	YLTNLEN	QVKNLED	QLAQLRP#	QIASHES	QKQFLML	EKQTLLQ	RMEILEK	EINFRES	DLEMKKE	QVKYLRE
