# Supplementary material for: Genome-wide analysis of the basic leucine zipper (bZIP) transcription factor gene family in six legume genomes
Source: BMC Genomics. 2015 Dec 10;16:1053. doi: 10.1186/s12864-015-2258-x (PMC4676100; doi:10.1186/s12864-015-2258-x)

Additional file 10. Chromosomal distributions of legume bZIP genes. Graphical (scaled) representation of physical locations for each legume bZIP gene on chromosomes. The duplicated bZIP pairs identified by syntenic relationship (using software *Mcscan*) were linked by lines. Tandem-duplicated genes on a particular chromosome are indicated in red font.

Genomic distribution of *GmbZIP* genes on *Glycine max* chromosomes.

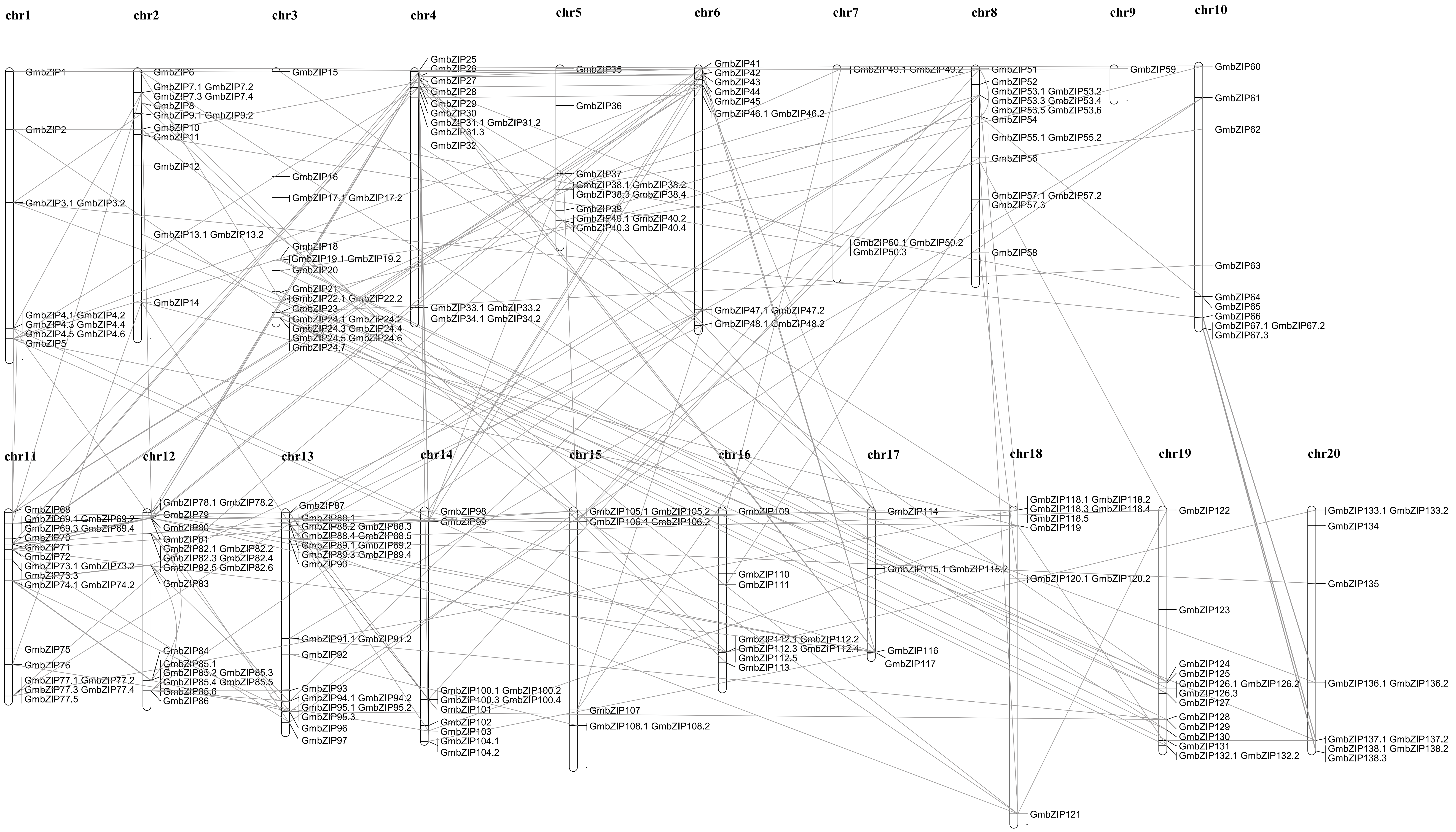

Genomic distribution of *MtbZIP* genes on ***Medicago truncatula*** chromosomes.

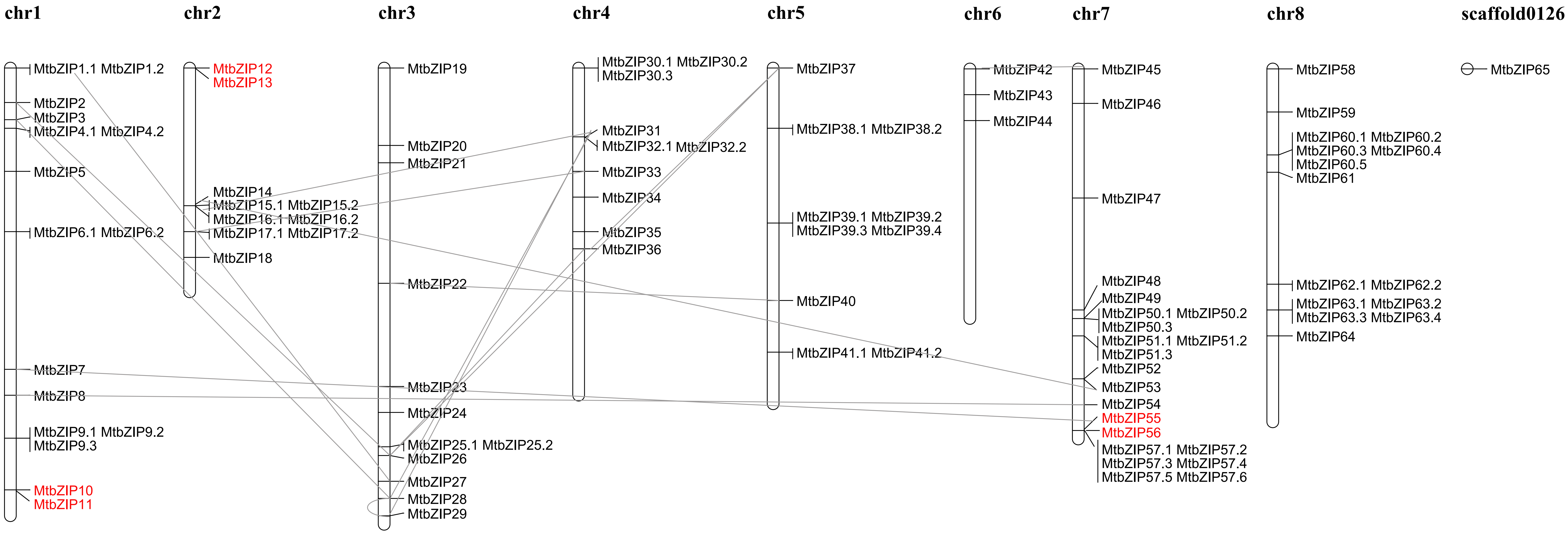

Genomic distribution of *PvbZIP* genes on ***Phaseolus vulgaris*** chromosomes.

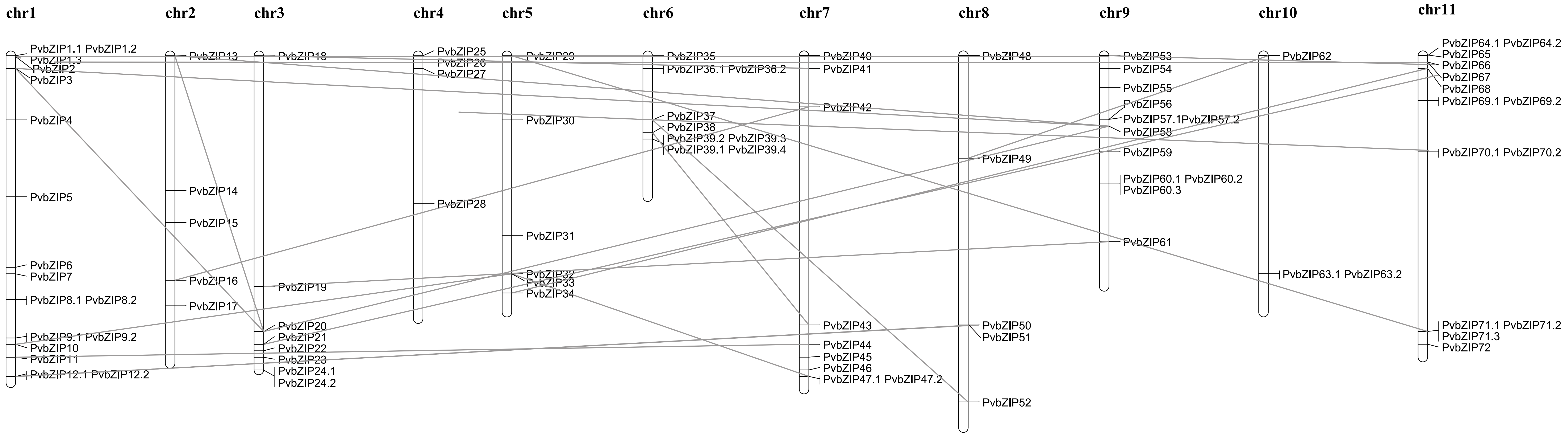

Genomic distribution of *CabZIP* genes on ***Cicer arietinum*** chromosomes.

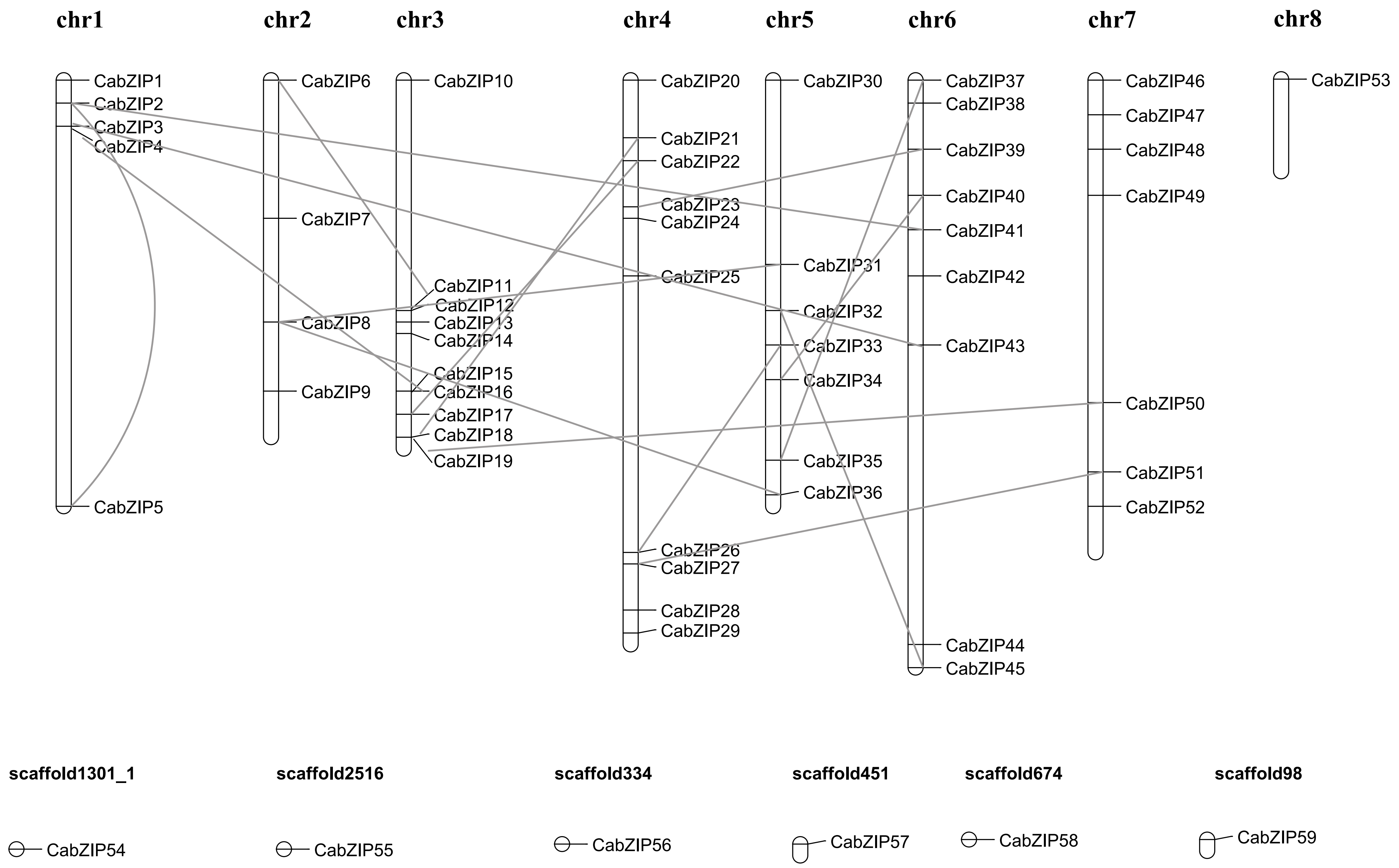

Genomic distribution of *CcbZIP* genes on ***Cajanus cajan*** chromosomes.

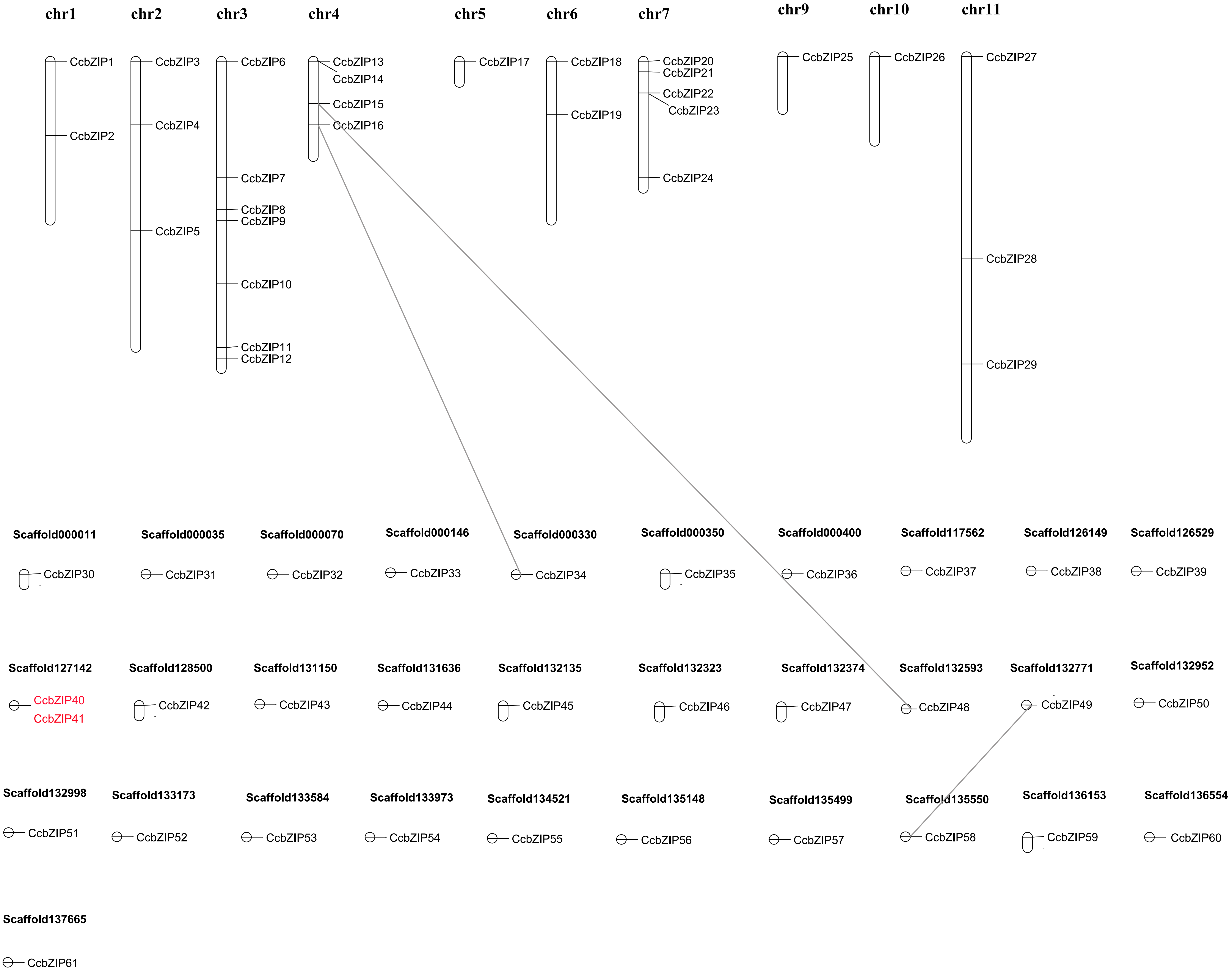

Genomic distribution of *LjbZIP* genes on ***Lotus japonicus*** chromosomes.

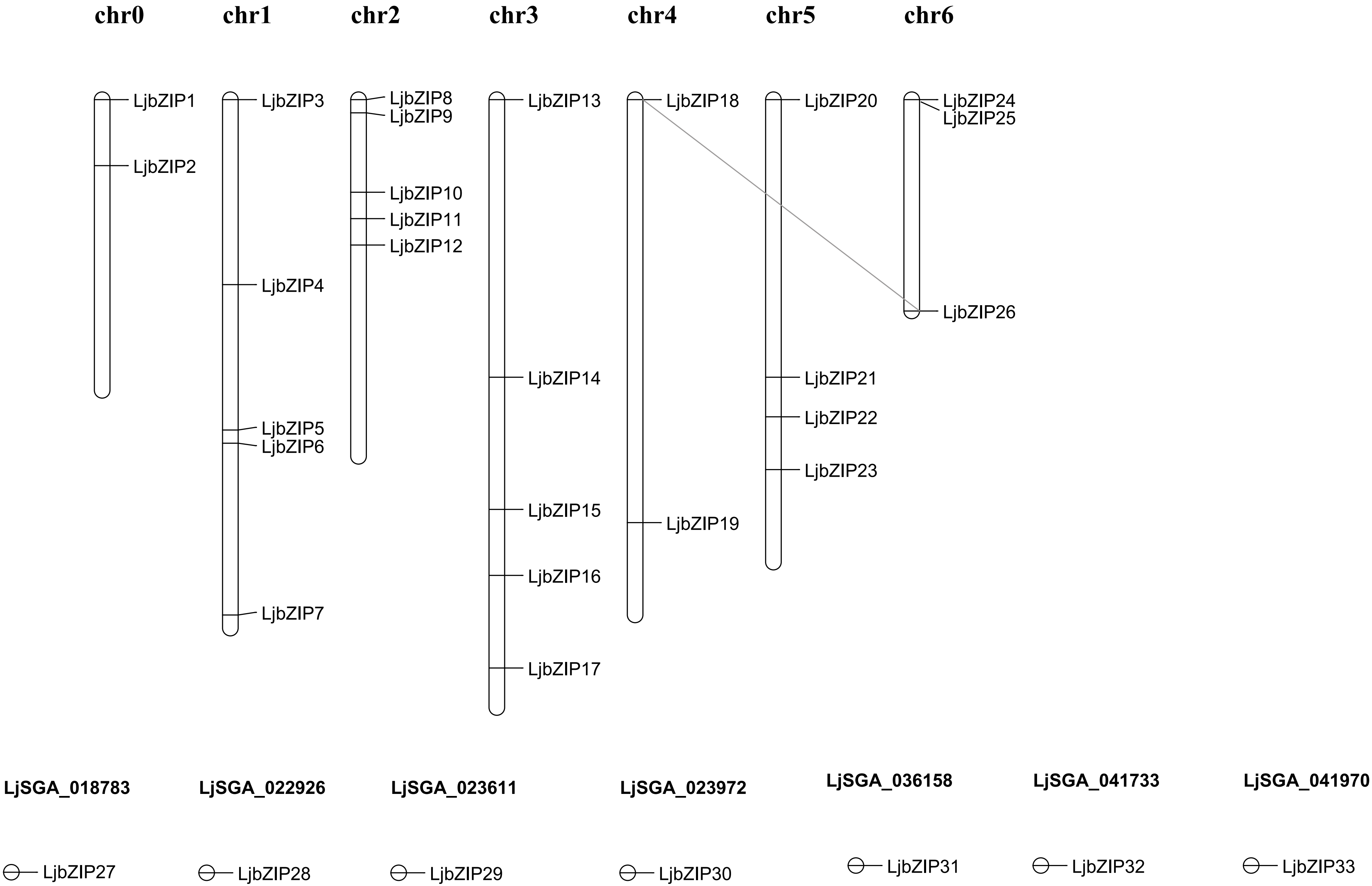

Supplement: Additional file 10: — Chromosomal distributions of legume bZIP genes. (PDF 2418 kb) [file 12864_2015_2258_MOESM10_ESM.pdf]
