# Supplementary material for: Genome-wide analysis of the basic leucine zipper (bZIP) transcription factor gene family in six legume genomes
Source: BMC Genomics. 2015 Dec 10;16:1053. doi: 10.1186/s12864-015-2258-x (PMC4676100; doi:10.1186/s12864-015-2258-x)

Additional file 3. The phylogenetic analysis of bZIP proteins based on the bZIP protein sequences from Arabidopsis (71 bZIP proteins) and six legumes (585 bZIP proteins) corresponding to figure 1. A total of 241 Gm, 99 Mt, 92 Pv, 59 Ca, 61 Cc, 33 Lj and 71 Arabidopsis bZIP protein sequences were aligned by Clustal X and the phylogenetic tree was constructed using PhyML by the maximum likelihood method. Bootstrap values are based on 100 replicates. The colored triangles symbolize the species which the bZIP proteins in each clade belong to.The legume bZIP proteins were grouped into 11 distinct clades (A–I, S, U), which are indicated by colored branches.

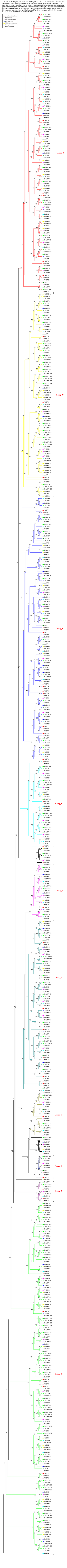

Supplement: Additional file 3: — The phylogenetic analysis of bZIP proteins based on the bZIP protein sequences from Arabidopsis (71 bZIP proteins) and six legumes (585 bZIP proteins) corresponding to Fig. 1 . (PDF 1003 kb) [file 12864_2015_2258_MOESM3_ESM.pdf]
